# Genetically determined telomere length and risk for haematologic diseases: results from large prospective cohorts and Mendelian Randomization analysis

**DOI:** 10.1038/s41408-024-01035-5

**Published:** 2024-03-18

**Authors:** Yang Li, Jia Chen, Ting Sun, Yunfei Chen, Rongfeng Fu, Xiaofan Liu, Feng Xue, Wei Liu, Mankai Ju, Xinyue Dai, Huan Dong, Huiyuan Li, Wentian Wang, Ying Chi, Lei Zhang

**Affiliations:** 1 State Key Laboratory of Experimental Hematology, National Clinical Research Center for Blood Diseases, Haihe Laboratory of Cell Ecosystem, Institute of Hematology & Blood Diseases Hospital, Chinese Academy of Medical Sciences & Peking Union Medical College, Tianjin Key Laboratory of Gene Therapy for Blood Diseases, CAMS Key Laboratory of Gene Therapy for Blood Diseases, Tianjin, 300020 China; 2 Tianjin Institutes of Health Science, Tianjin, 301600 China; 3 School of Population Medicine and Public Health, Chinese Academy of Medical Sciences & Peking Union Medical College, Beijing, 100730 China

**Keywords:** Haematological cancer, Risk factors

## Abstract

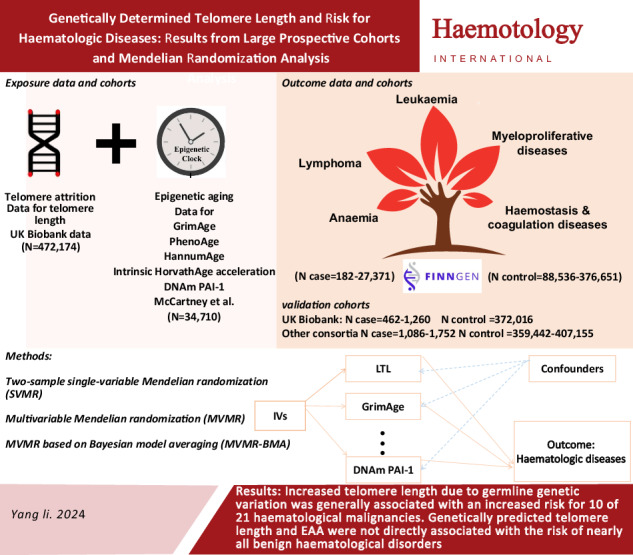

## To the Editor:

Telomere attrition and epigenetic modifications stand out as prominent molecular characteristics of aging-related biological processes and important risk factors for the development of haematologic diseases [1]. The relationship between telomere length and the risk of haematologic diseases has been extensively studied. However, the results of these studies have been conflicting [2]. The predictions of epigenetic clocks often deviate from chronological age, leading to a phenomenon known as epigenetic age acceleration (EAA) [3]. Empirical observations indicate that EAA is linked to an elevated risk of several health conditions [4]. However, this phenomenon has yet to be systematically evaluated for haematologic diseases. The objective of our study was to conduct a Mendelian randomization (MR) investigation, utilizing germline genetic variants as instrumental variables for both telomere length and EAA, to explore whether telomere length and EAA are associated with an increased risk of various haematologic diseases, including anaemia, lymphoma, leukaemia, myeloproliferative diseases, haemostasis and coagulation diseases, and other haematological disorders.

**Fig. 1:**
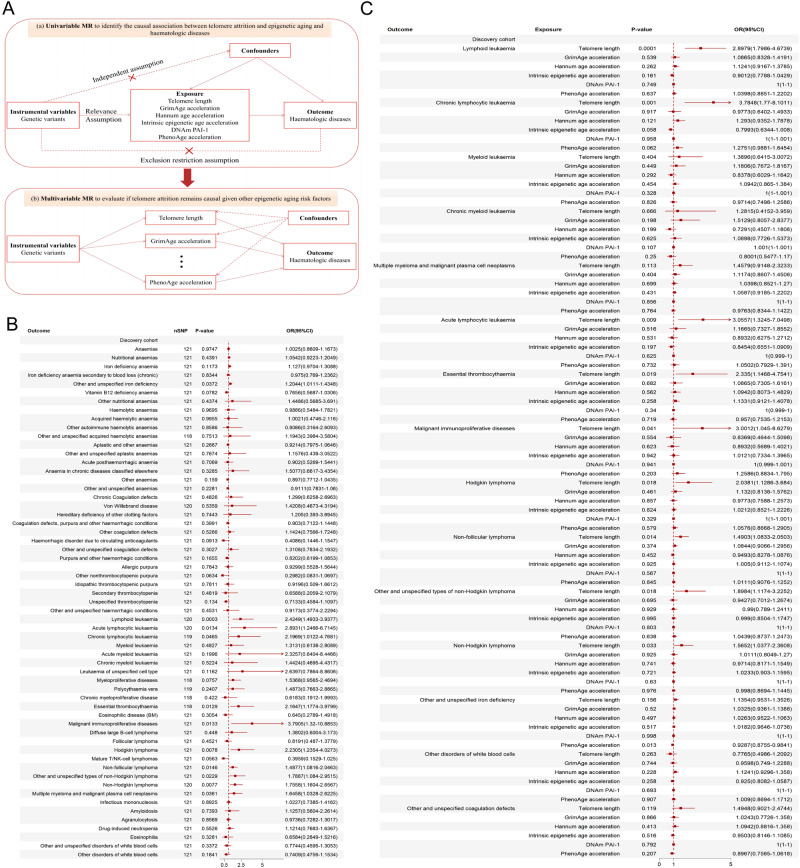
Study design and Mendelian randomization results. **A** Study design (a) The causal diagram illustrating the standard Mendelian randomization (MR) analysis with instrumental variables (IV) and the three necessary assumptions. (b) An illustrative diagram demonstrating the IV assumptions utilized in the multivariable MR model. **B** Two-sample single-variable Mendelian randomization results of telomere length on risk of multiple haematologic diseases in the discovery cohort. **C** Multivariable Mendelian randomization results of telomere length and five epigenetic age acceleration on risk of multiple haematologic diseases in the discovery cohort. MR, Mendelian randomization; IV, instrumental variable; DNAm PAI-1, DNA methylation-estimated plasminogen activator inhibitor-1; nSNP, number of single nucleotide polymorphism; OR, odds ratio; 95% CI, The 95% confidence intervals.

We initially conducted a two-sample single-variable MR (SVMR) study. This was then followed by verification using a validation dataset and different MR methods with different model assumptions. A series of multivariable MR (MVMR) analyses were then conducted to adjust for statistically significant risk factors. Furthermore, MVMR analysis based on Bayesian model averaging (MVMR-BMA) was performed to rank the aforementioned aging factors based on genetic evidence and assess whether telomere attrition, even in the presence of epigenetic aging, remains the true causal factor for haematologic diseases. Figure 1A presents an overview of the study design.

For telomere length analysis, data sources were derived from the UK Biobank, a comprehensive population-based cohort study comprising 472,174 participants [5]. To conduct SVMR analyses, we followed a rigorous selection process to derive a final set of 121 instrumental variables (Supplementary Tables 1 and 2). For epigenetic age acceleration measures, we acquired summary genetic association estimates from a recent GWAS meta-analysis of biological aging [6]. In certain cases, several SNPs were eliminated to address potential pleiotropic outliers. Specifically, we identified four independent SNPs for GrimAge, seven for HannumAge, 22 for Intrinsic HorvathAge, four for DNAm PAI-1 and 10 for PhenoAge (Supplementary Tables 3–8). Summary-level genetic association data for multiple haematologic disease outcomes were acquired from several sources (Table 1). In the discovery cohort, we obtained an extensive set of 59 GWASs from FinnGen [7]. Supplementary Fig. 1 demonstrates which specific haematologic diseases constitute each of the 59 GWAS summary statistic. In the validation cohort, GWAS data were sourced from both the UK Biobank cohort and several international consortia. In the MVMR analysis, we incorporated all the risk factors identified from the SVMR analysis, with a particular focus on assessing the significance of telomere length. To satisfy the instrumental SNP independence requirement in the MVMR-BMA, LD clumping was applied to the combination of SNPs of all aging risk factors. The detailed process of statistical analysis was provided in the Supplementary Method.

**Table 1:** Characteristics of exposures and outcome.

Variable	Source	Cases	Controls	Sample size
**Exopsure**				
Telomere length	UK Biobank data	472,174	/	472,174
DNAm GrimAge acceleration	PMID: 34187551	34,467	/	34,467
DNAm Hannum age acceleration	PMID: 34187551	34,449	/	34,449
Intrinsic epigenetic age acceleration	PMID: 34187551	34,461	/	34,461
DNAm PAI-1	PMID: 34187551	34,448	/	34,448
DNAm PhenoAge acceleration	PMID: 34187551	34,463	/	34,463
**Outcome of discovery cohort**				
Anaemias	FinnGen data D3_ANAEMIA	27,371	88,536	115,907
Nutritional anaemias	FinnGen data D3_NUTRIANAEMIA	7677	211,115	218,792
Iron deficiency anaemia	FinnGen data D3_ANAEMIA_IRONDEF	13,689	360,528	374,217
Iron deficiency anaemia secondary to blood loss (chronic)	FinnGen data D3_ANAEMIA_IRONDEF_BLOODLOSS	4852	360,528	365,380
Other and unspecified iron deficiency	FinnGen data D3_ANAEMIA_IRONDEF_NAS	10,208	360,528	370,736
Vitamin B12 deficiency anaemia	FinnGen data D3_ANAEMIA_B12_DEF	3351	360,528	363,879
Other nutritional anaemia	FinnGen data D3_NUTRIANAEMIAOTHER	283	360,528	360,811
Haemolytic anaemias	FinnGen data D3_HAEMOLYTICANAEMIA	838	376,439	377,277
Acquired haemolytic anaemia	FinnGen data D3_ACQHAEMOLYTICANAEMIA	606	376,439	377,045
Other autoimmune haemolytic anaemias	FinnGen data D3_AIHA_OTHER	280	376,439	376,719
Other and unspecified acquired haemolytic anaemias	FinnGen data D3_ACQHAEMOLYTICANAEMIANAS	241	376,439	376,680
Aplastic and other anaemias	FinnGen data D3_APLASTICANDOTHANAEMIA	6554	212,238	218,792
Other and unspecified aplastic anaemias	FinnGen data D3_OTHERAPLASTICANAEMIA	288	362,319	362,607
Acute posthaemorrhagic anaemia	FinnGen data D3_ACUTEPOSTBLEEDANAEMIA	976	362,319	363,295
Anaemia in chronic diseases classified elsewhere	FinnGen data D3_ANAEMIAINCHRONICDISEASE	585	362,319	362,904
Other anaemias	FinnGen data D3_OTHERANAEMIA	6005	212,238	218,243
Other and unspecified anaemias	FinnGen data D3_ANAEMIANAS	13,600	362,319	375,919
Chronic Coagulation defects	FinnGen data D3_COAGDEF	626	376,651	377,277
Von Willebrand disease	FinnGen data D3_VONVILLEBRAND	336	371,504	371,840
Hereditary deficiency of other clotting factors	FinnGen data D3_HEREDOTHCLOFACTORS	216	371,504	371,720
Coagulation defects, purpura and other haemorrhagic conditions	FinnGen data D3_COAGDEF_PURPUR_HAEMORRHAGIC	5773	371,504	377,277
Other coagulation defects	FinnGen data D3_COAGOTHER	1904	371,504	373,408
Haemorrhagic disorder due to circulating anticoagulants	FinnGen data D3_HAEMORRHAGCIRGUANTICO	267	371,504	371,771
Other and unspecified coagulation defects	FinnGen data D3_COAGDEFNAS	1217	371,504	372,721
Purpura and other haemorrhagic conditions	FinnGen data D3_PURPURA_AND3_OTHER_HAEMORRHAGIC	3900	371,504	375,404
Allergic purpura	FinnGen data D3_ALLERGPURPURA	856	371,504	372,360
Other nonthrombocytopenic purpura	FinnGen data D3_OTHNONTHROMBOCYTOPENPURPURA	214	371,504	371,718
Idiopathic thrombocytopenic purpura	FinnGen data D3_ITP	810	371,504	372,314
Secondary thrombocytopenia	FinnGen data D3_SCNDTHROMBOCYTOPENIA	298	371,504	371,802
Unspecified thrombocytopenia	FinnGen data D3_THROMBOCYTOPENIANAS	1869	371,504	373,373
Other and unspecified haemorrhagic conditions	FinnGen data D3_HAEMORRHAGICNAS	404	371,504	371,908
Lymphoid leukaemia	FinnGen data CD2_LYMPHOID_LEUKAEMIA_EXALLC	1493	299,952	301,445
Acute lymphocytic leukaemia	FinnGen data C3_ALL_EXALLC	184	287,136	287,320
Chronic lymphocytic leukaemia	FinnGen data C3_CLL_EXALLC	624	287,133	287,757
Myeloid leukaemia	FinnGen data CD2_MYELOID_LEUKAEMIA_EXALLC	674	299,952	300,626
Acute myeloid leukaemia	FinnGen data C3_AML_EXALLC	231	287,136	287,367
Chronic myeloid leukaemia [CML]BCR/ABL+	FinnGen data CML	232	375,158	375,390
Leukaemia of unspecified cell type	FinnGen data CD2_LEUKAEMIA_NAS_EXALLC	220	299,952	300,172
Myeloproliferative diseases	FinnGen data MYELOPROF_NONCML	1887	375,158	377,045
Polycythaemia vera	FinnGen data POLYCYTVERA	942	286,553	287,495
Chronic myeloproliferative disease	FinnGen data CHRONMYELOPRO	328	375,158	375,486
Essential thrombocythaemia	FinnGen data THROMBOCYTAEMIA	967	286,488	287,455
Eosinophilic disease (BM)	FinnGen data ESOSINOPHIL_DISEASE	398	212,144	212,542
Malignant immunoproliferative diseases	FinnGen data CD2_IMMUNOPROLIFERATIVE_EXALLC	223	299,952	300,175
Diffuse large B-cell lymphoma	FinnGen data C3_DLBCL_EXALLC	1010	287,137	288,147
Follicular lymphoma	FinnGen data CD2_FOLLICULAR_LYMPHOMA_EXALLC	1081	299,952	301,033
Hodgkin lymphoma	FinnGen data CD2_HODGKIN_LYMPHOMA_EXALLC	780	299,952	300,732
Mature T/NK-cell lymphomas	FinnGen data CD2_TNK_LYMPHOMA_EXALLC	335	299,952	300,287
Non-follicular lymphoma	FinnGen data CD2_NONFOLLICULAR_LYMPHOMA_EXALLC	2602	299,952	302,554
Other and unspecified types of non-Hodgkin lymphoma	FinnGen data CD2_NONHODGKIN_NAS_EXALLC	1088	299,952	301,040
Non-Hodgkin lymphoma	FinnGen data C3_NONHODGKIN_EXALLC	928	287,137	288,065
Multiple myeloma and malignant plasma cell neoplasms	FinnGen data CD2_MULTIPLE_MYELOMA_PLASMA_CELL_EXALLC	1249	299,952	301,201
Infectious mononucleosis	FinnGen data AB1_EBV	2353	367,472	369,825
Amyloidosis	FinnGen data E4_AMYLOIDOSIS	413	324,150	324,563
Agranulocytosis	FinnGen data D3_AGRANULOCYTOSIS	3234	370,400	373,634
Drug-induced neutropenia	FinnGen data DRUGADVERS_NEUTROPENIA	1978	375,299	377,277
Eosinophilia	FinnGen data D3_EOSINOPHILIA	182	215,755	215,937
Other and unspecified disorders of white blood cells	FinnGen data D3_WHITEBLOODCELLNAS	1077	370,400	371,477
Other disorders of white blood cells	FinnGen data D3_OTHERWHITECELL	1483	370,400	371,883
**Outcome of validation cohort**				
Leukaemia	UK Biobank data	1260	372,016	373,276
Lymphoid leukaemia	UK Biobank data	760	372,016	372,776
Myeloid leukaemia	UK Biobank data	462	372,016	372,478
Multiple myeloma	UK Biobank data	601	372,016	372,617
Myeloproliferative neoplasms	PMID: 33057200	1086	407,155	408,241
Lymphomas	UK Biobank data	1752	359,442	361,194

The SVMR results between genetically determined telomere length and haematologic diseases are presented in Fig. 1B. Genetically increased telomere length was associated with higher ORs (95% CIs) of disease in 10 of the 21 haematological malignancies (*P* < 0.05) (Supplementary Fig. 3). Associations (IVW ORs; [95% CIs] per 1-SD change in genetically increased telomere length; P-value) were observed: lymphoid leukaemia (2.4249; [1.4933–3.9377]; 0.0003), acute lymphocytic leukaemia (2.8931; [1.2466–6.7145]; 0.0134), chronic lymphocytic leukaemia (2.1969; [1.0122–4.7681]; 0.0465), essential thrombocythaemia (2.1647; [1.1774–3.9799]; 0.0129), malignant immunoproliferative diseases (3.7905; [1.3200–10.8853]; 0.0133), Hodgkin lymphoma (2.2305; [1.2354–4.0273]; 0.0078), non-Hodgkin lymphoma (1.7558; [1.1604–2.6567]; 0.0077), non-follicular lymphoma (1.4877; [1.0816–2.0463]; 0.0146), other and unspecified types of non-Hodgkin lymphoma (1.7887; [1.0840–2.9515]; 0.0229) and multiple myeloma and malignant plasma cell neoplasms (1.6458; [1.0328–2.6225]; 0.0361) (Fig. 1B). These significant results were successfully replicated in an independent validation cohort. (Supplementary Fig. 2a).

Utilizing a meta-analysis of IVW SVMR, we found no evidence of genetically predicted DNA methylation GrimAge acceleration associated with the risk of the mentioned haematologic diseases. Causal estimation showed that genetically determined Hannum age acceleration was associated with a lower risk of developing chronic myeloid leukaemia (OR = 0.5553 per year increase in Hannum age acceleration, 95% CI 0.3182–0.9690, *P* value = 0.0384). Our findings showed no evidence of causality between genetically predicted Intrinsic EAA and the aforementioned haematologic disorders. Genetically predicted higher levels of DNAm PAI-1 exhibited marginally significant causal associations with an increased risk of chronic myeloid leukaemia. We also found that higher PhenoAge acceleration was associated with increased risks of myeloid leukaemia (OR = 1.3018 per year increase in PhenoAge acceleration, 95% CI 1.0596–1.5994, *P* value = 0.0120), chronic lymphocytic leukaemia (OR = 1.2280, 95% CI 1.0118–1.4905, *P* value = 0.0376), and lymphoid leukaemia (OR = 1.1539, 95% CI 1.0267–1.2968, *P* value = 0.0163) (Supplementary Fig. 2b–f). The causal analysis of genetically determined 5 EAA in an independent validation cohort yielded similar results (Supplementary Fig. 4). The consistency of these above SVMR results was further supported by other MR methods, and no heterogeneity and horizontal pleiotropy were detected (Supplementary Tables 9 and 10).

Subsequently, our focus shifted to statistically significant haematological disorders identified through SVMR analysis. We conducted MVMR analysis to adjust and compare the impact of telomere length and the role of epigenetic age acceleration in the risk of these haematological disorders (Supplementary Tables 11 and 12). After adjusting for EAA using the MVMR-IVW method, telomere length was found to be associated with several haematological malignancy outcomes. In the discovery cohort, these outcomes included lymphoid leukaemia (IVW OR 2.8979, 95% CI 1.7986–4.6739, *P* <0.0001), chronic lymphocytic leukaemia (3.7848, 1.7700–8.1011, 0.0010), acute lymphocytic leukaemia (3.0557, 1.3245–7.0498, 0.0090), essential thrombocythaemia (2.3350, 1.1468–4.7541, 0.0190), malignant immunoproliferative diseases (3.0012, 1.0450–8.6379, 0.0410), Hodgkin lymphoma (2.0381, 1.1286–3.6840, 0.0180), non–follicular lymphoma (1.4903, 1.0833–2.0503, 0.0140), other and unspecified types of non-Hodgkin lymphoma (1.8984, 1.1174–3.2252, 0.0180) and non-Hodgkin lymphoma (1.5652, 1.0377–2.3608, 0.0330) (Fig. 1C). However, the significant association observed between telomere length and multiple myeloma and malignant plasma cell neoplasms in the SVMR model was attenuated in the MVMR model and was no longer significant (Fig. 1C). The effects of DNA methylation Hannum age acceleration and DNAm PAI-1 levels that were previously observed in the SVMR for chronic myeloid leukaemia were no longer significant in the MVMR after adjusting for other EAA and telomere length (Fig. 1C). However, in the validation cohort, after adjusting for telomere length and other EAA using MVMR-LASSO regression and MVMR-Egger, genetically predicted Hannum age acceleration remained significantly and positively associated with leukaemia, lymphoid leukaemia, myeloid leukaemia, multiple myeloma (Supplementary Table 13). Significant associations were observed between genetically predicted PhenoAge acceleration and myeloid leukaemia, other disorders of white blood cells, and other and unspecified coagulation defects in the SVMR were attenuated in the MVMR model, and the results were no longer significant (Fig. 1C). Similar results of MVMR analysis were also obtained in the validation cohort (Supplementary Fig. 5).

To prioritize aging-related risk factors for haematological diseases based on our univariable outcomes, we employed a novel multivariable approach, MVMR-BMA. During the model diagnostics, we successfully detected influential and outlying instrumental SNPs (Supplementary Fig. 6). Subsequently, we performed an analysis after eliminating influential and outlying SNPs. Supplementary Table 14 presents the top 10 models ranked by their model PP, along with the MIP and the model-averaged causal effect estimates of the six aging-related factors. Remarkably, the results revealed that telomere length had the strongest association with the risk of haematologic diseases when compared with the five EAA. Notably, analogous results were obtained when all 144 instrumental variables (IVs) were integrated into the analysis (Supplementary Table 15).

Our findings consistently align with results from prospective observational studies, which typically indicate an increased risk of lymphoma, non-Hodgkin lymphoma, and follicular lymphoma in individuals with longer telomeres [8–10]. However, the outcomes of an association cohort study, utilizing data from the UK Biobank, contradict our research findings. This study reveals a significantly higher prevalence of lymphoid and myeloid leukaemia in participants with shorter leukocyte telomere length [11]. These contradictory findings may be attributed to reverse causation in the retrospective studies, stemming from the absence of temporal information. Previous GWAS studies have revealed connections between longer telomeres and specific variations in multiple telomere-related genes such as TERT, TERC, and POT1 [12]. A recently published investigation highlighted that individuals with overly extended telomeres and an inherited ability to elongate telomeres due to POT1 dysfunction are more susceptible to developing lymphoid and myeloid clonal hematopoiesis [13].

The crucial role of epigenetic regulation in the development of haematologic cancers has been underscored by many studies. Our MR estimates for the association between EAA and various forms of leukaemia, namely lymphoid leukaemia, myeloid leukaemia, chronic lymphocytic leukaemia and chronic myeloid leukaemia were broadly consistent with the outcomes reported in the previous studies. For example, Maegawa et al. demonstrate that methylation changes arise as a function of age in normal hematopoiesis and are accelerated in MDS and at the transition from MDS to AML [14]. Nannini et al. discovered significant connections between EAA and the time to relapse among patients with chronic lymphocytic leukaemia [15]. Our study findings suggest that longer telomeres are linked to a higher risk of most haematologic malignancies, but genetically predicted telomere length and EAA do not significantly influence the risk of nearly all benign haematological disorders. This indicates the potential clinical relevance of telomere length, holding promising prospects for clinical implementation.

### Supplementary information

Supplemental Material

Supplementary tables

Supplementary Figure 6

## Data Availability

All data used in the current study are publicly available GWAS summary data.

## Code availability

For original data and code, please contact zhanglei1@ihcams.ac.cn.
