# Supplementary material for: Genetically determined telomere length and risk for haematologic diseases: results from large prospective cohorts and Mendelian Randomization analysis
Source: Blood Cancer J. 2024 Mar 18;14(1):48. doi: 10.1038/s41408-024-01035-5 (PMC10948832; doi:10.1038/s41408-024-01035-5)
Supplement: Supplementary file 1 — Supplemental Material [file 41408_2024_1035_MOESM1_ESM.docx]

**SUPPLEMENTAL MATERIAL**

**Genetically Determined Telomere Length and Risk for Haematologic Diseases: Results from Large Prospective Cohorts and Mendelian Randomization Analysis**

Yang Li^1,2*^, Jia Chen^1,2*^, Ting Sun^1,2*^, Yunfei Chen^1,2^, Rongfeng Fu^1,2^, Xiaofan Liu^1,2^, Feng Xue^1,2^, Wei Liu^1,2^, Mankai Ju^1,2^, Xinyue Dai^1,2^, Huan Dong^1,2^, Huiyuan Li^1,2^, Wentian Wang^1,2^, Ying Chi^1,2^, Lei Zhang^1,2,3^

1.State Key Laboratory of Experimental Hematology, National Clinical Research Center for Blood Diseases, Haihe Laboratory of Cell Ecosystem, Institute of Hematology & Blood Diseases Hospital, Chinese Academy of Medical Sciences & Peking Union Medical College, Tianjin Key Laboratory of Gene Therapy for Blood Diseases, CAMS Key Laboratory of Gene Therapy for Blood Diseases, Tianjin 300020, China;

2. Tianjin Institutes of Health Science, Tianjin 301600, China;

3. School of Population Medicine and Public Health, Chinese Academy of Medical Sciences & Peking Union Medical College, Beijing 100730, China.

*Yang Li, Jia Chen and Ting Sun contributed equally to this study and should be regarded as co-first authors.

Correspondence:

Lei Zhang, State Key Laboratory of Experimental Hematology, National Clinical Research Center for Blood Diseases, Haihe Laboratory of Cell Ecosystem, Institute of Hematology & Blood Diseases Hospital, Chinese Academy of Medical Sciences & Peking Union Medical College, Tianjin Key Laboratory of Gene Therapy for Blood Diseases, CAMS Key Laboratory of Gene Therapy for Blood Diseases, Tianjin 300020, China; Tianjin Institutes of Health Science, Tianjin 301600, China. Email: zhanglei1@ihcams.ac.cn.

**Supplementary Materials**

**Supplementary Table 1.** Index instrumental SNPs for telomere length and the effects, standard errors on haematologic diseases in the discovery analysis.

**Supplementary Table 2.** Index instrumental SNPs for telomere length and the effects, standard errors on haematologic diseases in the validation analysis.

**Supplementary Table 3.** Index instrumental SNPs for DNA methylation GrimAge acceleration and the effects, standard errors on haematologic diseases in the discovery analysis.

**Supplementary Table 4.** Index instrumental SNPs for five epigenetic age acceleration and the effects, standard errors on haematologic diseases in the validation analysis.

**Supplementary Table 5.** Index instrumental SNPs for DNA methylation Hannum age acceleration and the effects, standard errors on haematologic diseases in the discovery analysis.

**Supplementary Table 6.** Index instrumental SNPs for Intrinsic epigenetic age acceleration and the effects, standard errors on haematologic diseases in the discovery analysis.

**Supplementary Table 7.** Index instrumental SNPs for DNA methylation-estimated plasminogen activator inhibitor-1 levels and the effects, standard errors on haematologic diseases in the discovery analysis.

**Supplementary Table 8.** Index instrumental SNPs for DNA methylation PhenoAge acceleration and the effects, standard errors on haematologic diseases in the discovery analysis.

**Supplementary Table 9.** Two-sample single-variable estimates of genetically predicted telomere length on each haematologic disease outcome using various Mendelian randomization methods.

**Supplementary Table 10.** Two-sample single-variable estimates of genetically predicted five epigenetic age acceleration on each haematologic disease outcome using various Mendelian randomization methods.

**Supplementary Table 11.** Index instrumental SNPs for exposures and outcomes in the multivariable Mendelian randomization analysis in the discovery analysis.

**Supplementary Table 12.** Index instrumental SNPs for exposures and outcomes in the multivariable Mendelian randomization analysis in the validation analysis.

**Supplementary Table 13.** Multivariable estimates of genetically predicted telomere length and five epigenetic age acceleration on each haematologic disease outcome using various Mendelian randomization methods.

**Supplementary Table 14.** The ranking of models for haematologic diseases after model diagnostics in the multivariable Mendelian randomization analysis.

**Supplementary Table 15.** The ranking of models for haematologic diseases using all instrumental variables in the multivariable Mendelian randomization analysis.

**Supplementary Figure 1.** A fishbone diagram to demonstrate which specific haematologic diseases constitute each of the 59 GWAS summary statistics in this study.

**Supplementary Figure 2.** Two-sample single-variable Mendelian randomization results of telomere length and five epigenetic age acceleration on risk of multiple haematologic diseases. (a) The association between genetically increased telomere length and odds of haematologic diseases in the validation cohort. (b) The association between genetically predicted DNA methylation GrimAge acceleration and odds of haematologic diseases in the discovery cohort. (c) The association between genetically predicted DNA methylation Hannum age acceleration and odds of haematologic diseases in the discovery cohort. (d) The association between genetically predicted Intrinsic epigenetic age acceleration and odds of haematologic diseases in the discovery cohort. (e) The association between genetically predicted DNAm PAI-1 levels and odds of haematologic diseases in the discovery cohort. (f) The association between genetically predicted DNA methylation PhenoAge acceleration and odds of haematologic diseases in the discovery cohort.

**Supplementary Figure 3.** Scatter plot of associations of genetic risk of telomere length on risk of 10 haematological malignancies.

**Supplementary Figure 4.** Two-sample single-variable Mendelian randomization results of telomere length and five epigenetic age acceleration on risk of multiple haematologic diseases in the validation cohort.

**Supplementary Figure 5.** Multivariable Mendelian randomization results of telomere length and five epigenetic age acceleration on risk of multiple haematologic diseases in the validation cohort.

**Supplementary Figure 6.** Model diagnostics in multivariable MR based on BMA.The predicted associations with haematologic diseases based on the model including telomere length (x-axis) are plotted against the observed associations with haematologic diseases (y-axis). These are the top models when keeping outliers and influential genetic variants in the analysis. (a) Cook's distance for the influential points; (b) the q-statistic for outliers. Any genetic variant with q value larger than threshold or Cook's distance larger than the median of the relevant F-distribution is marked by a label indicating the gene region.

**Supplementary Method**

1. Genome-wide association studies data sources and instrument selection in MR

2. Statistical analysis

**Supplementary Table 1-13 displayed in Supplementary Table 1-13.xlsx**

**Supplementary Table 14.** The ranking of models for haematologic diseases after model diagnostics in the multivariable Mendelian randomization analysis.

|  | Model | PP | casual effect | Risk factor | MIP | Model-averaged causal effect | Empirical p-value | FDR |
| --- | --- | --- | --- | --- | --- | --- | --- | --- |
| **Discovery cohort** | |  |  |  |  |  |  |  |
| Lymphoid leukaemia (SNP=143) | |  |  |  |  |  |  |  |
| 1 | telomere length | 0.853 | 0.613 | GrimAge acceleration | 7.02E-02 | 0.01 | 0.96770323 | 0.9987001 |
| 2 | GrimAge acceleration,telomere length | 0.048 | 0.143,0.630 | Hannum age acceleration | 4.41E-02 | 0.004 | 0.98150185 | 0.9987001 |
| 3 | Hannum age acceleration,telomere length | 0.028 | 0.089,0.615 | Intrinsic EAA | 1.90E-02 | -0.001 | 0.99710029 | 0.9987001 |
| 4 | GrimAge acceleration | 0.018 | 0.126 | DNAm PAI-1 | 4.88E-05 | 0 | 0.99470053 | 0.9987001 |
| 5 | Hannum age acceleration | 0.013 | 0.088 | PhenoAge acceleration | 1.93E-02 | 0 | 0.99870013 | 0.9987001 |
| 6 | PhenoAge acceleration,telomere length | 0.012 | 0.028,0.626 | telomere length | 9.57E-01 | 0.588 | 0.00249975 | 0.0149985 |
| 7 | Intrinsic EAA,telomere length | 0.011 | -0.028,0.624 |  |  |  |  |  |
| 8 | PhenoAge acceleration | 0.005 | 0.007 |  |  |  |  |  |
| 9 | Intrinsic EAA | 0.005 | -0.014 |  |  |  |  |  |
| 10 | GrimAge acceleration,Hannum age acceleration,telomere length | 0.001 | 0.104,0.051,0.627 |  |  |  |  |  |
| Chronic lymphocytic leukaemia (SNP=143) | |  |  |  |  |  |  |  |
| 1 | telomere length | 0.315 | 0.479 | GrimAge acceleration | 0.165847639 | 0.032 | 0.77322268 | 0.9622038 |
| 2 | Hannum age acceleration | 0.241 | 0.226 | Hannum age acceleration | 0.323329473 | 0.073 | 0.08549145 | 0.470153 |
| 3 | PhenoAge acceleration | 0.131 | 0.164 | Intrinsic EAA | 0.053455224 | -0.001 | 0.96220378 | 0.9622038 |
| 4 | GrimAge acceleration | 0.116 | 0.208 | DNAm PAI-1 | 0.000132246 | 0 | 0.94990501 | 0.9622038 |
| 5 | Hannum age acceleration,telomere length | 0.051 | 0.227,0.484 | PhenoAge acceleration | 0.192431568 | 0.032 | 0.18278172 | 0.470153 |
| 6 | PhenoAge acceleration,telomere length | 0.037 | 0.181,0.544 | telomere length | 0.443802551 | 0.216 | 0.23507649 | 0.470153 |
| 7 | Intrinsic EAA | 0.029 | 0.016 |  |  |  |  |  |
| 8 | GrimAge acceleration,telomere length | 0.026 | 0.221,0.501 |  |  |  |  |  |
| 9 | GrimAge acceleration,Hannum age acceleration | 0.01 | 0.062,0.204 |  |  |  |  |  |
| 10 | Hannum age acceleration,PhenoAge acceleration | 0.008 | 0.168,0.095 |  |  |  |  |  |
| Myeloid leukaemia (SNP=144) | |  |  |  |  |  |  |  |
| 1 | telomere length | 0.42 | 0.244 | GrimAge acceleration | 0.208431885 | 0.014 | 0.5234477 | 0.5936406 |
| 2 | GrimAge acceleration | 0.177 | 0.061 | Hannum age acceleration | 0.164411245 | -0.012 | 0.3907609 | 0.5936406 |
| 3 | Hannum age acceleration | 0.138 | -0.068 | Intrinsic EAA | 0.116602216 | 0.005 | 0.4969503 | 0.5936406 |
| 4 | PhenoAge acceleration | 0.098 | -0.019 | DNAm PAI-1 | 0.000587584 | 0 | 0.1846815 | 0.5717428 |
| 5 | Intrinsic EAA | 0.098 | 0.039 | PhenoAge acceleration | 0.116695689 | -0.002 | 0.5936406 | 0.5936406 |
| 6 | GrimAge acceleration,telomere length | 0.015 | 0.069,0.251 | telomere length | 0.4638648 | 0.113 | 0.1905809 | 0.5717428 |
| 7 | Hannum age acceleration,telomere length | 0.011 | -0.069,0.246 |  |  |  |  |  |
| 8 | PhenoAge acceleration,telomere length | 0.008 | -0.012,0.24 |  |  |  |  |  |
| 9 | Intrinsic EAA,telomere length | 0.008 | 0.033,0.234 |  |  |  |  |  |
| 10 | GrimAge acceleration,Hannum age acceleration | 0.007 | 0.148, -0.121 |  |  |  |  |  |
| Chronic myeloid leukaemia (SNP=144) | |  |  |  |  |  |  |  |
| 1 | telomere length | 0.23 | 0.142 | GrimAge acceleration | 0.201249879 | 0.026 | 0.70242976 | 0.7147285 |
| 2 | PhenoAge acceleration | 0.208 | -0.197 | Hannum age acceleration | 0.263863346 | -0.057 | 0.15308469 | 0.3061694 |
| 3 | Hannum age acceleration | 0.203 | -0.21 | Intrinsic EAA | 0.116575415 | -0.008 | 0.71472853 | 0.7147285 |
| 4 | GrimAge acceleration | 0.141 | 0.092 | DNAm PAI-1 | 0.001476633 | 0 | 0.07179282 | 0.3061694 |
| 5 | Intrinsic EAA | 0.088 | -0.078 | PhenoAge acceleration | 0.264217474 | -0.052 | 0.11978802 | 0.3061694 |
| 6 | GrimAge acceleration,Hannum age acceleration | 0.02 | 0.284, -0.304 | telomere length | 0.290417999 | 0.041 | 0.71342866 | 0.7147285 |
| 7 | Hannum age acceleration,telomere length | 0.018 | -0.21,0.146 |  |  |  |  |  |
| 8 | PhenoAge acceleration,telomere length | 0.017 | -0.194,0.098 |  |  |  |  |  |
| 9 | GrimAge acceleration,PhenoAge acceleration | 0.016 | 0.224, -0.241 |  |  |  |  |  |
| 10 | GrimAge acceleration,telomere length | 0.012 | 0.096,0.149 |  |  |  |  |  |
| Multiple myeloma and malignant plasma cell neoplasms (SNP=144) | |  |  |  |  |  |  |  |
| 1 | telomere length | 0.473 | 0.329 | GrimAge acceleration | 0.196592943 | 0.026 | 0.5282472 | 0.7923708 |
| 2 | GrimAge acceleration | 0.153 | 0.135 | Hannum age acceleration | 0.159089684 | 0.016 | 0.3612639 | 0.7225277 |
| 3 | Hannum age acceleration | 0.127 | 0.103 | Intrinsic EAA | 0.130148145 | 0.01 | 0.3056694 | 0.7225277 |
| 4 | Intrinsic EAA | 0.105 | 0.081 | DNAm PAI-1 | 0.000142816 | 0 | 0.8963104 | 0.9140086 |
| 5 | PhenoAge acceleration | 0.046 | 0.038 | PhenoAge acceleration | 0.06160903 | 0.002 | 0.9140086 | 0.9140086 |
| 6 | GrimAge acceleration,telomere length | 0.031 | 0.146,0.347 | telomere length | 0.551498479 | 0.182 | 0.1346865 | 0.7225277 |
| 7 | Hannum age acceleration,telomere length | 0.021 | 0.102,0.325 |  |  |  |  |  |
| 8 | Intrinsic EAA,telomere length | 0.015 | 0.073,0.301 |  |  |  |  |  |
| 9 | PhenoAge acceleration,telomere length | 0.009 | 0.049,0.348 |  |  |  |  |  |
| 10 | GrimAge acceleration,Hannum age acceleration | 0.004 | 0.079,0.074 |  |  |  |  |  |
| Acute lymphocytic leukaemia (SNP=141) | |  |  |  |  |  |  |  |
| 1 | telomere length | 0.455 | 0.588 | GrimAge acceleration | 0.077203499 | 0.002 | 0.98710129 | 0.98710129 |
| 2 | Intrinsic EAA | 0.169 | -0.202 | Hannum age acceleration | 0.083337073 | -0.009 | 0.95860414 | 0.98710129 |
| 3 | Intrinsic EAA,telomere length | 0.128 | -0.222,0.65 | Intrinsic EAA | 0.329014205 | -0.069 | 0.072392761 | 0.217178282 |
| 4 | PhenoAge acceleration | 0.059 | -0.143 | DNAm PAI-1 | 0.000137841 | 0 | 0.96080392 | 0.98710129 |
| 5 | Hannum age acceleration | 0.045 | -0.122 | PhenoAge acceleration | 0.09740594 | -0.013 | 0.853414659 | 0.98710129 |
| 6 | GrimAge acceleration | 0.038 | -0.008 | telomere length | 0.663716574 | 0.399 | 0.02489751 | 0.149385061 |
| 7 | PhenoAge acceleration,telomere length | 0.023 | -0.124,0.554 |  |  |  |  |  |
| 8 | Hannum age acceleration,telomere length | 0.022 | -0.125,0.593 |  |  |  |  |  |
| 9 | GrimAge acceleration,telomere length | 0.018 | 0.008,0.589 |  |  |  |  |  |
| 10 | GrimAge acceleration,Intrinsic EAA | 0.007 | 0.062, -0.209 |  |  |  |  |  |
| Essential thrombocythaemia (SNP=139) | |  |  |  |  |  |  |  |
| 1 | telomere length | 0.629 | 0.616 | GrimAge acceleration | 0.077690242 | 0.01 | 0.97330267 | 0.97690231 |
| 2 | Intrinsic EAA | 0.095 | 0.152 | Hannum age acceleration | 0.093057374 | 0.013 | 0.887311269 | 0.97690231 |
| 3 | Intrinsic EAA,telomere length | 0.066 | 0.142,0.586 | Intrinsic EAA | 0.174325546 | 0.026 | 0.188481152 | 0.565443456 |
| 4 | Hannum age acceleration | 0.042 | 0.138 | DNAm PAI-1 | 0.000125633 | 0 | 0.944105589 | 0.97690231 |
| 5 | Hannum age acceleration,telomere length | 0.042 | 0.146,0.63 | PhenoAge acceleration | 0.04657552 | 0.003 | 0.97690231 | 0.97690231 |
| 6 | GrimAge acceleration,telomere length | 0.034 | 0.149,0.636 | telomere length | 0.801196015 | 0.494 | 0.01439856 | 0.086391361 |
| 7 | GrimAge acceleration | 0.033 | 0.128 |  |  |  |  |  |
| 8 | PhenoAge acceleration,telomere length | 0.022 | 0.095,0.662 |  |  |  |  |  |
| 9 | PhenoAge acceleration | 0.018 | 0.066 |  |  |  |  |  |
| 10 | GrimAge acceleration,Intrinsic EAA | 0.003 | 0.087,0.144 |  |  |  |  |  |
| Malignant immunoproliferative diseases (SNP=144) | |  |  |  |  |  |  |  |
| 1 | telomere length | 0.483 | 0.488 | GrimAge acceleration | 0.180803753 | -0.022 | 0.804819518 | 0.900009999 |
| 2 | GrimAge acceleration | 0.133 | -0.118 | Hannum age acceleration | 0.118309797 | -0.003 | 0.900009999 | 0.900009999 |
| 3 | PhenoAge acceleration | 0.097 | 0.107 | Intrinsic EAA | 0.116154802 | 0.01 | 0.709329067 | 0.900009999 |
| 4 | Intrinsic EAA | 0.086 | 0.091 | DNAm PAI-1 | 0.000266574 | 0 | 0.832916708 | 0.900009999 |
| 5 | Hannum age acceleration | 0.084 | -0.021 | PhenoAge acceleration | 0.137984703 | 0.016 | 0.561543846 | 0.900009999 |
| 6 | GrimAge acceleration,telomere length | 0.026 | -0.105,0.48 | telomere length | 0.569870848 | 0.278 | 0.03269673 | 0.196180382 |
| 7 | PhenoAge acceleration,telomere length | 0.022 | 0.123,0.516 |  |  |  |  |  |
| 8 | Hannum age acceleration,telomere length | 0.017 | -0.022,0.488 |  |  |  |  |  |
| 9 | Intrinsic EAA,telomere length | 0.016 | 0.079,0.471 |  |  |  |  |  |
| 10 | GrimAge acceleration,PhenoAge acceleration | 0.007 | -0.198,0.146 |  |  |  |  |  |
| Hodgkin lymphoma (SNP=142) | |  |  |  |  |  |  |  |
| 1 | telomere length | 0.356 | 0.457 | GrimAge acceleration | 0.290659186 | 0.069 | 0.203779622 | 0.407559244 |
| 2 | GrimAge acceleration | 0.169 | 0.24 | Hannum age acceleration | 0.079973587 | 0.008 | 0.930206979 | 0.930206979 |
| 3 | PhenoAge acceleration | 0.097 | 0.14 | Intrinsic EAA | 0.132063203 | 0.016 | 0.364363564 | 0.546545345 |
| 4 | GrimAge acceleration,telomere length | 0.088 | 0.249,0.473 | DNAm PAI-1 | 0.000171525 | 0 | 0.868813119 | 0.930206979 |
| 5 | Intrinsic EAA | 0.085 | 0.128 | PhenoAge acceleration | 0.187642726 | 0.027 | 0.201279872 | 0.407559244 |
| 6 | PhenoAge acceleration,telomere length | 0.067 | 0.156,0.505 | telomere length | 0.572430988 | 0.265 | 0.088091191 | 0.407559244 |
| 7 | Hannum age acceleration | 0.047 | 0.118 |  |  |  |  |  |
| 8 | Intrinsic EAA,telomere length | 0.026 | 0.115,0.416 |  |  |  |  |  |
| 9 | Hannum age acceleration,telomere length | 0.019 | 0.114,0.449 |  |  |  |  |  |
| 10 | GrimAge acceleration,Intrinsic EAA | 0.009 | 0.199,0.105 |  |  |  |  |  |
| Non-follicular lymphoma (SNP=143) | |  |  |  |  |  |  |  |
| 1 | telomere length | 0.89 | 0.345 | GrimAge acceleration | 0.048846753 | 0.003 | 0.98030197 | 0.99550045 |
| 2 | GrimAge acceleration，telomere length | 0.026 | 0.069, 0.355 | Hannum age acceleration | 0.028793647 | 0.001 | 0.9910009 | 0.99550045 |
| 3 | GrimAge acceleration | 0.022 | 0.057 | Intrinsic EAA | 0.01671743 | 0 | 0.99330067 | 0.99550045 |
| 4 | Hannum age acceleration | 0.014 | 0.035 | DNAm PAI-1 | 6.33E-05 | 0 | 0.97370263 | 0.99550045 |
| 5 | Hannum age acceleration，telomere length | 0.014 | 0.034，0.344 | PhenoAge acceleration | 0.017604334 | 0 | 0.99550045 | 0.99550045 |
| 6 | PhenoAge acceleration | 0.008 | -0.006 | telomere length | 0.946617401 | 0.327 | 0.00549945 | 0.0329967 |
| 7 | PhenoAge acceleration，telomere length | 0.008 | 0.005，0.348 |  |  |  |  |  |
| 8 | Intrinsic EAA | 0.008 | 0.012 |  |  |  |  |  |
| 9 | Intrinsic EAA，telomere length | 0.008 | 0.003，0.344 |  |  |  |  |  |
| 10 | GrimAge acceleration，Hannum age acceleration，telomere length | 0 | 0.06，0.011，0.353 |  |  |  |  |  |
| Other and unspecified types of non-Hodgkin lymphoma (SNP=143) | |  |  |  |  |  |  |  |
| 1 | telomere length | 0.869 | 0.592 | GrimAge acceleration | 0.061990492 | -0.007 | 0.9830017 | 0.99820018 |
| 2 | GrimAge acceleration,telomere length | 0.033 | -0.107,0.582 | Hannum age acceleration | 0.028178624 | -0.001 | 0.99820018 | 0.99820018 |
| 3 | GrimAge acceleration | 0.025 | -0.122 | Intrinsic EAA | 0.021635149 | 0 | 0.99670033 | 0.99820018 |
| 4 | Hannum age acceleration,telomere length | 0.016 | -0.024,0.593 | DNAm PAI-1 | 6.19E-05 | 0 | 0.98950105 | 0.99820018 |
| 5 | PhenoAge acceleration,telomere length | 0.013 | 0.023,0.601 | PhenoAge acceleration | 0.023285164 | 0 | 0.99820018 | 0.99820018 |
| 6 | Intrinsic EAA,telomere length | 0.012 | 0.01,0.588 | telomere length | 0.945621655 | 0.56 | 0.00189981 | 0.01139886 |
| 7 | Hannum age acceleration | 0.01 | -0.02 |  |  |  |  |  |
| 8 | Intrinsic EAA | 0.008 | 0.026 |  |  |  |  |  |
| 9 | PhenoAge acceleration | 0.008 | 0.003 |  |  |  |  |  |
| 10 | GrimAge acceleration,Hannum age acceleration,telomere length | 0.001 | -0.122,0.02,0.579 |  |  |  |  |  |
| Non-Hodgkin lymphoma (SNP=142) | |  |  |  |  |  |  |  |
| 1 | telomere length | 0.831 | 0.403 | GrimAge acceleration | 0.060600222 | -0.001 | 0.9750025 | 0.97770223 |
| 2 | GrimAge acceleration | 0.043 | -0.026 | Hannum age acceleration | 0.041774059 | 0 | 0.97770223 | 0.97770223 |
| 3 | Hannum age acceleration | 0.029 | -0.009 | Intrinsic EAA | 0.034845972 | 0.001 | 0.96350365 | 0.97770223 |
| 4 | Intrinsic EAA | 0.025 | 0.024 | DNAm PAI-1 | 0.000100987 | 0 | 0.946405359 | 0.97770223 |
| 5 | PhenoAge acceleration | 0.025 | -0.015 | PhenoAge acceleration | 0.035378092 | 0 | 0.9750025 | 0.97770223 |
| 6 | GrimAge acceleration,telomere length | 0.016 | -0.014,0.401 | telomere length | 0.876344013 | 0.353 | 0.01249875 | 0.074992501 |
| 7 | Hannum age acceleration,telomere length | 0.011 | -0.011,0.404 |  |  |  |  |  |
| 8 | PhenoAge acceleration,telomere length | 0.009 | -0.002,0.402 |  |  |  |  |  |
| 9 | Intrinsic EAA,telomere length | 0.009 | 0.012,0.398 |  |  |  |  |  |
| 10 | GrimAge acceleration,Hannum age acceleration | 0.001 | -0.026,0.001 |  |  |  |  |  |
| Other and unspecified iron deficiency (SNP=143) | |  |  |  |  |  |  |  |
| 1 | telomere length | 0.786 | 0.164 | GrimAge acceleration | 0.054801515 | 0 | 0.96390361 | 0.96580342 |
| 2 | PhenoAge acceleration | 0.083 | -0.034 | Hannum age acceleration | 0.038738652 | 0 | 0.96580342 | 0.96580342 |
| 3 | GrimAge acceleration | 0.046 | 0.006 | Intrinsic EAA | 0.028775226 | 0 | 0.96090391 | 0.96580342 |
| 4 | Hannum age acceleration | 0.032 | 0.006 | DNAm PAI-1 | 7.95E-05 | 0 | 0.941305869 | 0.96580342 |
| 5 | Intrinsic EAA | 0.024 | -0.002 | PhenoAge acceleration | 0.095475179 | -0.003 | 0.631836816 | 0.96580342 |
| 6 | PhenoAge acceleration,telomere length | 0.009 | -0.028,0.151 | telomere length | 0.812026128 | 0.133 | 0.03079692 | 0.184781522 |
| 7 | GrimAge acceleration,telomere length | 0.007 | 0.011,0.166 |  |  |  |  |  |
| 8 | Hannum age acceleration,telomere length | 0.005 | 0.005,0.164 |  |  |  |  |  |
| 9 | Intrinsic EAA,telomere length | 0.004 | -0.007,0.167 |  |  |  |  |  |
| 10 | Hannum age acceleration,PhenoAge acceleration | 0.001 | 0.037, -0.05 |  |  |  |  |  |
| Other disorders of white blood cells (SNP=143) | |  |  |  |  |  |  |  |
| 1 | telomere length | 0.527 | -0.235 | GrimAge acceleration | 0.135876294 | 0.005 | 0.823317668 | 0.823317668 |
| 2 | Hannum age acceleration | 0.119 | 0.06 | Hannum age acceleration | 0.143609373 | 0.009 | 0.456554345 | 0.823317668 |
| 3 | GrimAge acceleration | 0.115 | 0.039 | Intrinsic EAA | 0.095814638 | -0.004 | 0.613438656 | 0.823317668 |
| 4 | PhenoAge acceleration | 0.099 | 0.05 | DNAm PAI-1 | 0.000201124 | 0 | 0.763223678 | 0.823317668 |
| 5 | Intrinsic EAA | 0.075 | -0.036 | PhenoAge acceleration | 0.1194201 | 0.006 | 0.491650835 | 0.823317668 |
| 6 | Hannum age acceleration,telomere length | 0.014 | 0.061, -0.237 | telomere length | 0.572450298 | -0.134 | 0.119288071 | 0.715728427 |
| 7 | GrimAge acceleration,telomere length | 0.013 | 0.032, -0.231 |  |  |  |  |  |
| 8 | PhenoAge acceleration,telomere length | 0.01 | 0.042, -0.218 |  |  |  |  |  |
| 9 | Intrinsic EAA,telomere length | 0.008 | -0.03, -0.223 |  |  |  |  |  |
| 10 | Intrinsic EAA,PhenoAge acceleration | 0.005 | -0.089,0.106 |  |  |  |  |  |
| Other and unspecified coagulation defects (SNP=143) | |  |  |  |  |  |  |  |
| 1 | telomere length | 0.505 | 0.324 | GrimAge acceleration | 0.113300037 | 0.004 | 0.899210079 | 0.900309969 |
| 2 | PhenoAge acceleration | 0.197 | -0.105 | Hannum age acceleration | 0.081067823 | 0.002 | 0.900309969 | 0.900309969 |
| 3 | GrimAge acceleration | 0.087 | 0.026 | Intrinsic EAA | 0.087154008 | -0.005 | 0.713428657 | 0.900309969 |
| 4 | Intrinsic EAA | 0.068 | -0.053 | DNAm PAI-1 | 0.000173887 | 0 | 0.837916208 | 0.900309969 |
| 5 | Hannum age acceleration | 0.06 | 0.014 | PhenoAge acceleration | 0.240713854 | -0.026 | 0.111688831 | 0.357864214 |
| 6 | PhenoAge acceleration,telomere length | 0.022 | -0.096,0.286 | telomere length | 0.563645441 | 0.182 | 0.119288071 | 0.357864214 |
| 7 | GrimAge acceleration,telomere length | 0.013 | 0.036,0.328 |  |  |  |  |  |
| 8 | Intrinsic EAA,telomere length | 0.012 | -0.063,0.348 |  |  |  |  |  |
| 9 | Hannum age acceleration,telomere length | 0.009 | 0.012,0.324 |  |  |  |  |  |
| 10 | GrimAge acceleration,PhenoAge acceleration | 0.008 | 0.11, -0.128 |  |  |  |  |  |
| **Validation cohort** | |  |  |  |  |  |  |  |
| Leukaemia(SNP=143) | |  |  |  |  |  |  |  |
| 1 | telomere length | 0.996 | 0.002 | GrimAge acceleration | 0.000244 | 0 | 0.99990001 | 0.99990001 |
| 2 | Hannum age acceleration | 0.002 | 0 | Hannum age acceleration | 0.003183068 | 0 | 0.99790021 | 0.99990001 |
| 3 | Hannum age acceleration,telomere length | 0.001 | 0,0.002 | Intrinsic EAA | 0.000159206 | 0 | 0.99970003 | 0.99990001 |
| 4 | GrimAge acceleration | 0 | 0 | DNAm PAI-1 | 2.88E-07 | 0 | 0.99970003 | 0.99990001 |
| 5 | Intrinsic EAA | 0 | 0 | PhenoAge acceleration | 0.000140532 | 0 | 0.99980002 | 0.99990001 |
| 6 | PhenoAge acceleration | 0 | 0 | telomere length | 0.997788203 | 0.002 | 0.00039996 | 0.00239976 |
| 7 | GrimAge acceleration,telomere length | 0 | 0,0.002 |  |  |  |  |  |
| 8 | Intrinsic EAA,telomere length | 0 | 0,0.002 |  |  |  |  |  |
| 9 | PhenoAge acceleration,telomere length | 0 | 0,0.002 |  |  |  |  |  |
| 10 | DNAm PAI-1 | 0 | 0 |  |  |  |  |  |
| Lymphoid leukaemia (SNP=142) | |  |  |  |  |  |  |  |
| 1 | telomere length | 0.999 | 0.002 | GrimAge acceleration | 5.43E-05 | 0 | 1 | 1 |
| 2 | Hannum age acceleration,telomere length | 0.001 | 0,0.002 | Hannum age acceleration | 0.000601037 | 0 | 0.99960004 | 1 |
| 3 | Intrinsic EAA,telomere length | 0 | 0,0.002 | Intrinsic EAA | 6.38E-05 | 0 | 0.99990001 | 1 |
| 4 | GrimAge acceleration,telomere length | 0 | 0,0.002 | DNAm PAI-1 | 1.03E-07 | 0 | 1 | 1 |
| 5 | PhenoAge acceleration,telomere length | 0 | 0,0.002 | PhenoAge acceleration | 2.73E-05 | 0 | 1 | 1 |
| 6 | Hannum age acceleration | 0 | 0 | telomere length | 0.999993242 | 0.002 | 1.00E-04 | 0.00059994 |
| 7 | Intrinsic EAA | 0 | 0 |  |  |  |  |  |
| 8 | GrimAge acceleration | 0 | 0 |  |  |  |  |  |
| 9 | GrimAge acceleration,Hannum age acceleration,telomere length | 0 | -0.001,0.001,0.002 |  |  |  |  |  |
| 10 | PhenoAge acceleration | 0 | 0 |  |  |  |  |  |
| Myeloid leukaemia (SNP=143) | |  |  |  |  |  |  |  |
| 1 | Hannum age acceleration | 0.396 | 0 | GrimAge acceleration | 0.244600518 | 0 | 0.219678032 | 0.659034097 |
| 2 | GrimAge acceleration | 0.245 | 0 | Hannum age acceleration | 0.396527757 | 0 | 0.03909609 | 0.234576542 |
| 3 | telomere length | 0.212 | 0 | Intrinsic EAA | 0.08940226 | 0 | 0.463853615 | 0.893510649 |
| 4 | Intrinsic EAA | 0.089 | 0 | DNAm PAI-1 | 0.000180293 | 0 | 0.734726527 | 0.893510649 |
| 5 | PhenoAge acceleration | 0.057 | 0 | PhenoAge acceleration | 0.057545961 | 0 | 0.822017798 | 0.893510649 |
| 6 | DNAm PAI-1 | 0 | 0 | telomere length | 0.211973011 | 0 | 0.893510649 | 0.893510649 |
| 7 | Hannum age acceleration,Intrinsic EAA | 0 | 0,0 |  |  |  |  |  |
| 8 | Hannum age acceleration,telomere length | 0 | 0,0 |  |  |  |  |  |
| 9 | Hannum age acceleration,PhenoAge acceleration | 0 | 0,0 |  |  |  |  |  |
| 10 | GrimAge acceleration,telomere length | 0 | 0,0 |  |  |  |  |  |
| Multiple myeloma (SNP=140) | |  |  |  |  |  |  |  |
| 1 | telomere length | 0.929 | 0.001 | GrimAge acceleration | 0.01714055 | 0 | 0.98760124 | 0.98760124 |
| 2 | Hannum age acceleration | 0.033 | 0 | Hannum age acceleration | 0.033561446 | 0 | 0.95380462 | 0.98760124 |
| 3 | GrimAge acceleration | 0.017 | 0 | Intrinsic EAA | 0.008841664 | 0 | 0.98580142 | 0.98760124 |
| 4 | PhenoAge acceleration | 0.011 | 0 | DNAm PAI-1 | 4.21E-05 | 0 | 0.96950305 | 0.98760124 |
| 5 | Intrinsic EAA | 0.009 | 0 | PhenoAge acceleration | 0.010938368 | 0 | 0.9860014 | 0.98760124 |
| 6 | Hannum age acceleration,telomere length | 0 | 0,0.001 | telomere length | 0.929631457 | 0.001 | 0.00919908 | 0.055194481 |
| 7 | DNAm PAI-1 | 0 | 0 |  |  |  |  |  |
| 8 | GrimAge acceleration,telomere length | 0 | 0,0.001 |  |  |  |  |  |
| 9 | PhenoAge acceleration,telomere length | 0 | 0,0.001 |  |  |  |  |  |
| 10 | Intrinsic EAA,telomere length | 0 | 0,0.001 |  |  |  |  |  |
| Lymphomas (SNP=146) | |  |  |  |  |  |  |  |
| 1 | telomere length | 0.718 | 0.002 | GrimAge acceleration | 0.043671757 | 0 | 0.96020398 | 0.97380262 |
| 2 | Hannum age acceleration | 0.204 | 0.001 | Hannum age acceleration | 0.204547453 | 0 | 0.158384162 | 0.475152485 |
| 3 | GrimAge acceleration | 0.043 | 0 | Intrinsic EAA | 0.016031486 | 0 | 0.97240276 | 0.97380262 |
| 4 | PhenoAge acceleration | 0.018 | 0 | DNAm PAI-1 | 4.80E-05 | 0 | 0.944705529 | 0.97380262 |
| 5 | Intrinsic EAA | 0.016 | 0 | PhenoAge acceleration | 0.01809215 | 0 | 0.97380262 | 0.97380262 |
| 6 | Hannum age acceleration,telomere length | 0.001 | 0.001,0.002 | telomere length | 0.718551283 | 0.001 | 0.065693431 | 0.394160584 |
| 7 | GrimAge acceleration,telomere length | 0 | 0.001,0.002 |  |  |  |  |  |
| 8 | PhenoAge acceleration,telomere length | 0 | 0 |  |  |  |  |  |
| 9 | DNAm PAI-1 | 0 | 0 |  |  |  |  |  |
| 10 | Intrinsic EAA,telomere length | 0 | 0,0.002 |  |  |  |  |  |
| Top ten models (combination of risk factors) ranked by the model posterior probability and all risk factors ranked by the marginal inclusion probability in the analysis after model diagnostics based on genetic variants. Causal effects are log odds ratios for haematologic diseases. Empirical P-values are computed using 200 permutations and adjustment for multiple testing via the Benjamini and Hochberg false discovery rate (FDR) procedure. SNP=single nucleotide polymorphism, PP=posterior probability, MIP =Marginal inclusion probability, DNAm PAI-1= DNA methylation-estimated plasminogen activator inhibitor-1, EAA=epigenetic age acceleration. | | | | | | | | |

**Supplementary Table 15.** The ranking of models for haematologic diseases using all instrumental variables in the multivariable Mendelian randomization analysis.

|  | Model | PP | casual effect | Risk factor | MIP | Model-averaged causal effect | Empirical p-value | FDR |
| --- | --- | --- | --- | --- | --- | --- | --- | --- |
| **Discovery cohort** | |  |  |  |  |  |  |  |
| Lymphoid leukaemia (SNP=144) | |  |  |  |  |  |  |  |
| 1 | telomere length | 0.888 | 0.829 | GrimAge acceleration | 4.88E-02 | 0.007 | 0.99020098 | 0.99990001 |
| 2 | GrimAge acceleration, telomere length | 0.046 | 0.14,0.847 | Hannum age acceleration | 3.90E-02 | 0.004 | 0.98860114 | 0.99990001 |
| 3 | Hannum age acceleration, telomere length | 0.035 | 0.103,0.826 | Intrinsic epigenetic age acceleration | 1.39E-02 | 0 | 0.99940006 | 0.99990001 |
| 4 | PhenoAge acceleration, telomere length | 0.013 | 0.035,0.843 | DNAm PAI-1 | 3.50E-05 | 0 | 0.99990001 | 0.99990001 |
| 5 | Intrinsic epigenetic age acceleration, telomere length | 0.012 | -0.026,0.839 | PhenoAge acceleration | 1.49E-02 | 0 | 0.99990001 | 0.99990001 |
| 6 | GrimAge acceleration, Hannum age acceleration, telomere length | 0.001 | 0.085,0.072,0.837 | telomere length | 9.98E-01 | 0.828 | 0.00009999 | 0.00059994 |
| 7 | Hannum age acceleration, Intrinsic epigenetic age acceleration, telomere length | 0.001 | 0.158, -0.085,0.856 |  |  |  |  |  |
| 8 | Hannum age acceleration | 0.001 | 0.107 |  |  |  |  |  |
| 9 | GrimAge acceleration, Intrinsic epigenetic age acceleration, telomere length | 0.001 | 0.157, -0.043,0.865 |  |  |  |  |  |
| 10 | GrimAge acceleration | 0.001 | 0.113 |  |  |  |  |  |
| Chronic lymphocytic leukaemia (SNP=144) | |  |  |  |  |  |  |  |
| 1 | telomere length | 0.543 | 0.752 | GrimAge acceleration | 8.95E-02 | 0.016 | 0.96670333 | 0.9939006 |
| 2 | Hannum age acceleration,telomere length | 0.112 | 0.248,0.747 | Hannum age acceleration | 2.44E-01 | 0.061 | 0.1539846 | 0.4619538 |
| 3 | Hannum age acceleration | 0.109 | 0.251 | Intrinsic epigenetic age acceleration | 3.28E-02 | -0.001 | 0.99390061 | 0.9939006 |
| 4 | PhenoAge acceleration,telomere length | 0.067 | 0.19,0.815 | DNAm PAI-1 | 7.90E-05 | 0 | 0.99130087 | 0.9939006 |
| 5 | GrimAge acceleration,telomere length | 0.041 | 0.215,0.775 | PhenoAge acceleration | 1.24E-01 | 0.022 | 0.5270473 | 0.9939006 |
| 6 | PhenoAge acceleration | 0.041 | 0.164 | telomere length | 7.92E-01 | 0.601 | 0.01249875 | 0.0749925 |
| 7 | GrimAge acceleration | 0.034 | 0.19 |  |  |  |  |  |
| 8 | Intrinsic epigenetic age acceleration | 0.011 | 0.03 |  |  |  |  |  |
| 9 | Intrinsic epigenetic age acceleration,telomere length | 0.01 | 0.011,0.749 |  |  |  |  |  |
| 10 | GrimAge acceleration,Hannum age acceleration,telomere length | 0.005 | 0.049,0.231,0.752 |  |  |  |  |  |
| Myeloid leukaemia (SNP=144) | |  |  |  |  |  |  |  |
| 1 | telomere length | 0.42 | 0.244 | GrimAge acceleration | 0.208431885 | 0.014 | 0.5234477 | 0.5936406 |
| 2 | GrimAge acceleration | 0.177 | 0.061 | Hannum age acceleration | 0.164411245 | -0.012 | 0.3907609 | 0.5936406 |
| 3 | Hannum age acceleration | 0.138 | -0.068 | Intrinsic epigenetic age acceleration | 0.116602216 | 0.005 | 0.4969503 | 0.5936406 |
| 4 | PhenoAge acceleration | 0.098 | -0.019 | DNAm PAI-1 | 0.000587584 | 0 | 0.1846815 | 0.5717428 |
| 5 | Intrinsic epigenetic age acceleration | 0.098 | 0.039 | PhenoAge acceleration | 0.116695689 | -0.002 | 0.5936406 | 0.5936406 |
| 6 | GrimAge acceleration,telomere length | 0.015 | 0.069,0.251 | telomere length | 0.4638648 | 0.113 | 0.1905809 | 0.5717428 |
| 7 | Hannum age acceleration,telomere length | 0.011 | -0.069,0.246 |  |  |  |  |  |
| 8 | PhenoAge acceleration,telomere length | 0.008 | -0.012,0.24 |  |  |  |  |  |
| 9 | Intrinsic epigenetic age acceleration,telomere length | 0.008 | 0.033,0.234 |  |  |  |  |  |
| 10 | GrimAge acceleration,Hannum age acceleration | 0.007 | 0.148, -0.121 |  |  |  |  |  |
| Chronic myeloid leukaemia (SNP=144) | |  |  |  |  |  |  |  |
| 1 | telomere length | 0.23 | 0.142 | GrimAge acceleration | 0.201249879 | 0.026 | 0.70242976 | 0.7147285 |
| 2 | PhenoAge acceleration | 0.208 | -0.197 | Hannum age acceleration | 0.263863346 | -0.057 | 0.15308469 | 0.3061694 |
| 3 | Hannum age acceleration | 0.203 | -0.21 | Intrinsic epigenetic age acceleration | 0.116575415 | -0.008 | 0.71472853 | 0.7147285 |
| 4 | GrimAge acceleration | 0.141 | 0.092 | DNAm PAI-1 | 0.001476633 | 0 | 0.07179282 | 0.3061694 |
| 5 | Intrinsic epigenetic age acceleration | 0.088 | -0.078 | PhenoAge acceleration | 0.264217474 | -0.052 | 0.11978802 | 0.3061694 |
| 6 | GrimAge acceleration,Hannum age acceleration | 0.02 | 0.284, -0.304 | telomere length | 0.290417999 | 0.041 | 0.71342866 | 0.7147285 |
| 7 | Hannum age acceleration,telomere length | 0.018 | -0.21,0.146 |  |  |  |  |  |
| 8 | PhenoAge acceleration,telomere length | 0.017 | -0.194,0.098 |  |  |  |  |  |
| 9 | GrimAge acceleration,PhenoAge acceleration | 0.016 | 0.224, -0.241 |  |  |  |  |  |
| 10 | GrimAge acceleration,telomere length | 0.012 | 0.096,0.149 |  |  |  |  |  |
| Multiple myeloma and malignant plasma cell neoplasms (SNP=144) | |  |  |  |  |  |  |  |
| 1 | telomere length | 0.473 | 0.329 | GrimAge acceleration | 0.196592943 | 0.026 | 0.5282472 | 0.7923708 |
| 2 | GrimAge acceleration | 0.153 | 0.135 | Hannum age acceleration | 0.159089684 | 0.016 | 0.3612639 | 0.7225277 |
| 3 | Hannum age acceleration | 0.127 | 0.103 | Intrinsic epigenetic age acceleration | 0.130148145 | 0.01 | 0.3056694 | 0.7225277 |
| 4 | Intrinsic epigenetic age acceleration | 0.105 | 0.081 | DNAm PAI-1 | 0.000142816 | 0 | 0.8963104 | 0.9140086 |
| 5 | PhenoAge acceleration | 0.046 | 0.038 | PhenoAge acceleration | 0.06160903 | 0.002 | 0.9140086 | 0.9140086 |
| 6 | GrimAge acceleration,telomere length | 0.031 | 0.146,0.347 | telomere length | 0.551498479 | 0.182 | 0.1346865 | 0.7225277 |
| 7 | Hannum age acceleration,telomere length | 0.021 | 0.102,0.325 |  |  |  |  |  |
| 8 | Intrinsic epigenetic age acceleration,telomere length | 0.015 | 0.073,0.301 |  |  |  |  |  |
| 9 | PhenoAge acceleration,telomere length | 0.009 | 0.049,0.348 |  |  |  |  |  |
| 10 | GrimAge acceleration,Hannum age acceleration | 0.004 | 0.079,0.074 |  |  |  |  |  |
| Acute lymphocytic leukaemia (SNP=142) | |  |  |  |  |  |  |  |
| 1 | telomere length | 0.616 | 0.589 | GrimAge acceleration | 0.089399298 | 0 | 0.97970203 | 0.97970203 |
| 2 | Intrinsic epigenetic age acceleration | 0.078 | -0.134 | Hannum age acceleration | 0.098818365 | -0.011 | 0.918708129 | 0.97970203 |
| 3 | Hannum age acceleration | 0.059 | -0.117 | Intrinsic epigenetic age acceleration | 0.141903499 | -0.02 | 0.358264174 | 0.97970203 |
| 4 | GrimAge acceleration | 0.052 | -0.012 | DNAm PAI-1 | 0.000165196 | 0 | 0.935606439 | 0.97970203 |
| 5 | Intrinsic epigenetic age acceleration,telomere length | 0.05 | -0.152,0.632 | PhenoAge acceleration | 0.07720873 | -0.007 | 0.935406459 | 0.97970203 |
| 6 | PhenoAge acceleration | 0.048 | -0.096 | telomere length | 0.747958317 | 0.443 | 0.01079892 | 0.064793521 |
| 7 | Hannum age acceleration,telomere length | 0.029 | -0.12,0.594 |  |  |  |  |  |
| 8 | GrimAge acceleration,telomere length | 0.025 | 0.004,0.59 |  |  |  |  |  |
| 9 | PhenoAge acceleration,telomere length | 0.02 | -0.078,0.568 |  |  |  |  |  |
| 10 | GrimAge acceleration,Intrinsic epigenetic age acceleration | 0.003 | 0.033, -0.137 |  |  |  |  |  |
| Essential thrombocythaemia (SNP=144) | |  |  |  |  |  |  |  |
| 1 | telomere length | 0.67 | 0.703 | GrimAge acceleration | 0.068293967 | 0.008 | 0.9810019 | 0.98390161 |
| 2 | Intrinsic epigenetic age acceleration | 0.08 | 0.16 | Hannum age acceleration | 0.106685173 | 0.018 | 0.818418158 | 0.98390161 |
| 3 | Hannum age acceleration | 0.057 | 0.169 | Intrinsic epigenetic age acceleration | 0.132712693 | 0.02 | 0.318968103 | 0.95690431 |
| 4 | Intrinsic epigenetic age acceleration,telomere length | 0.042 | 0.143,0.654 | DNAm PAI-1 | 0.000115504 | 0 | 0.949705029 | 0.98390161 |
| 5 | Hannum age acceleration,telomere length | 0.041 | 0.167,0.699 | PhenoAge acceleration | 0.03848273 | 0.002 | 0.98390161 | 0.98390161 |
| 6 | GrimAge acceleration | 0.033 | 0.112 | telomere length | 0.802415141 | 0.563 | 0.01889811 | 0.113388661 |
| 7 | GrimAge acceleration,telomere length | 0.027 | 0.134,0.719 |  |  |  |  |  |
| 8 | PhenoAge acceleration | 0.017 | 0.051 |  |  |  |  |  |
| 9 | PhenoAge acceleration,telomere length | 0.015 | 0.075,0.731 |  |  |  |  |  |
| 10 | GrimAge acceleration,Intrinsic epigenetic age acceleration | 0.002 | 0.055,0.155 |  |  |  |  |  |
| Malignant immunoproliferative diseases (SNP=144) | |  |  |  |  |  |  |  |
| 1 | telomere length | 0.483 | 0.488 | GrimAge acceleration | 0.180803753 | -0.022 | 0.804819518 | 0.900009999 |
| 2 | GrimAge acceleration | 0.133 | -0.118 | Hannum age acceleration | 0.118309797 | -0.003 | 0.900009999 | 0.900009999 |
| 3 | PhenoAge acceleration | 0.097 | 0.107 | Intrinsic epigenetic age acceleration | 0.116154802 | 0.01 | 0.709329067 | 0.900009999 |
| 4 | Intrinsic epigenetic age acceleration | 0.086 | 0.091 | DNAm PAI-1 | 0.000266574 | 0 | 0.832916708 | 0.900009999 |
| 5 | Hannum age acceleration | 0.084 | -0.021 | PhenoAge acceleration | 0.137984703 | 0.016 | 0.561543846 | 0.900009999 |
| 6 | GrimAge acceleration,telomere length | 0.026 | -0.105,0.48 | telomere length | 0.569870848 | 0.278 | 0.03269673 | 0.196180382 |
| 7 | PhenoAge acceleration,telomere length | 0.022 | 0.123,0.516 |  |  |  |  |  |
| 8 | Hannum age acceleration,telomere length | 0.017 | -0.022,0.488 |  |  |  |  |  |
| 9 | Intrinsic epigenetic age acceleration,telomere length | 0.016 | 0.079,0.471 |  |  |  |  |  |
| 10 | GrimAge acceleration,PhenoAge acceleration | 0.007 | -0.198,0.146 |  |  |  |  |  |
| Hodgkin lymphoma (SNP=144) | |  |  |  |  |  |  |  |
| 1 | telomere length | 0.694 | 0.494 | GrimAge acceleration | 0.131960843 | 0.02 | 0.874812519 | 0.96350365 |
| 2 | GrimAge acceleration | 0.073 | 0.144 | Hannum age acceleration | 0.064047563 | 0.005 | 0.96350365 | 0.96350365 |
| 3 | GrimAge acceleration,telomere length | 0.05 | 0.159,0.512 | Intrinsic epigenetic age acceleration | 0.06132637 | 0.004 | 0.915108489 | 0.96350365 |
| 4 | Intrinsic epigenetic age acceleration | 0.038 | 0.075 | DNAm PAI-1 | 0.000220146 | 0 | 0.775422458 | 0.96350365 |
| 5 | Hannum age acceleration | 0.037 | 0.075 | PhenoAge acceleration | 0.061278696 | 0.004 | 0.939206079 | 0.96350365 |
| 6 | PhenoAge acceleration | 0.032 | 0.065 | telomere length | 0.810976886 | 0.402 | 0.01689831 | 0.101389861 |
| 7 | PhenoAge acceleration,telomere length | 0.024 | 0.082,0.523 |  |  |  |  |  |
| 8 | Hannum age acceleration,telomere length | 0.021 | 0.073,0.492 |  |  |  |  |  |
| 9 | Intrinsic epigenetic age acceleration,telomere length | 0.018 | 0.063,0.473 |  |  |  |  |  |
| 10 | GrimAge acceleration,Intrinsic epigenetic age acceleration | 0.002 | 0.121,0.063 |  |  |  |  |  |
| Non-follicular lymphoma (SNP=144) | |  |  |  |  |  |  |  |
| 1 | telomere length | 0.893 | 0.345 | GrimAge acceleration | 0.044855477 | 0.002 | 0.9850015 | 0.99350065 |
| 2 | GrimAge acceleration | 0.023 | 0.042 | Hannum age acceleration | 0.025161583 | 0 | 0.99350065 | 0.99350065 |
| 3 | GrimAge acceleration,telomere length | 0.02 | 0.053,0.353 | Intrinsic epigenetic age acceleration | 0.019097371 | 0 | 0.9930007 | 0.99350065 |
| 4 | Hannum age acceleration | 0.014 | -0.01 | DNAm PAI-1 | 7.16E-05 | 0 | 0.96760324 | 0.99350065 |
| 5 | PhenoAge acceleration | 0.011 | -0.003 | PhenoAge acceleration | 0.02028864 | 0 | 0.99270073 | 0.99350065 |
| 6 | Intrinsic epigenetic age acceleration | 0.011 | 0.011 | telomere length | 0.940209687 | 0.325 | 0.00559944 | 0.03359664 |
| 7 | Hannum age acceleration,telomere length | 0.01 | -0.011,0.346 |  |  |  |  |  |
| 8 | PhenoAge acceleration,telomere length | 0.008 | 0.008,0.349 |  |  |  |  |  |
| 9 | Intrinsic epigenetic age acceleration,telomere length | 0.008 | 0.002,0.345 |  |  |  |  |  |
| 10 | GrimAge acceleration,Hannum age acceleration | 0 | 0.069, -0.035 |  |  |  |  |  |
| Other and unspecified types of non-Hodgkin lymphoma (SNP=144) | |  |  |  |  |  |  |  |
| 1 | telomere length | 0.829 | 0.519 | GrimAge acceleration | 0.066263903 | -0.004 | 0.97890211 | 0.98710129 |
| 2 | GrimAge acceleration | 0.041 | -0.063 | Hannum age acceleration | 0.041619611 | 0 | 0.98690131 | 0.98710129 |
| 3 | Hannum age acceleration | 0.025 | -0.004 | Intrinsic epigenetic age acceleration | 0.033841508 | 0.001 | 0.98320168 | 0.98710129 |
| 4 | GrimAge acceleration,telomere length | 0.022 | -0.047,0.513 | DNAm PAI-1 | 9.50E-05 | 0 | 0.96490351 | 0.98710129 |
| 5 | Intrinsic epigenetic age acceleration | 0.021 | 0.027 | PhenoAge acceleration | 0.034761347 | 0 | 0.98710129 | 0.98710129 |
| 6 | PhenoAge acceleration | 0.02 | 0.003 | telomere length | 0.889904428 | 0.462 | 0.00779922 | 0.04679532 |
| 7 | Hannum age acceleration,telomere length | 0.014 | -0.006,0.519 |  |  |  |  |  |
| 8 | PhenoAge acceleration,telomere length | 0.012 | 0.02,0.527 |  |  |  |  |  |
| 9 | Intrinsic epigenetic age acceleration,telomere length | 0.011 | 0.013,0.514 |  |  |  |  |  |
| 10 | GrimAge acceleration,Hannum age acceleration | 0.001 | -0.083,0.026 |  |  |  |  |  |
| Non-Hodgkin lymphoma (SNP=142) | |  |  |  |  |  |  |  |
| 1 | telomere length | 0.831 | 0.403 | GrimAge acceleration | 0.060600222 | -0.001 | 0.9750025 | 0.97770223 |
| 2 | GrimAge acceleration | 0.043 | -0.026 | Hannum age acceleration | 0.041774059 | 0 | 0.97770223 | 0.97770223 |
| 3 | Hannum age acceleration | 0.029 | -0.009 | Intrinsic epigenetic age acceleration | 0.034845972 | 0.001 | 0.96350365 | 0.97770223 |
| 4 | Intrinsic epigenetic age acceleration | 0.025 | 0.024 | DNAm PAI-1 | 0.000100987 | 0 | 0.946405359 | 0.97770223 |
| 5 | PhenoAge acceleration | 0.025 | -0.015 | PhenoAge acceleration | 0.035378092 | 0 | 0.9750025 | 0.97770223 |
| 6 | GrimAge acceleration,telomere length | 0.016 | -0.014,0.401 | telomere length | 0.876344013 | 0.353 | 0.01249875 | 0.074992501 |
| 7 | Hannum age acceleration,telomere length | 0.011 | -0.011,0.404 |  |  |  |  |  |
| 8 | PhenoAge acceleration,telomere length | 0.009 | -0.002,0.402 |  |  |  |  |  |
| 9 | Intrinsic epigenetic age acceleration,telomere length | 0.009 | 0.012,0.398 |  |  |  |  |  |
| 10 | GrimAge acceleration,Hannum age acceleration | 0.001 | -0.026,0.001 |  |  |  |  |  |
| Other and unspecified iron deficiency (SNP=144) | |  |  |  |  |  |  |  |
| 1 | telomere length | 0.56 | 0.162 | GrimAge acceleration | 0.050260874 | 0.001 | 0.96460354 | 0.96460354 |
| 2 | PhenoAge acceleration | 0.3 | -0.05 | Hannum age acceleration | 0.038037389 | 0 | 0.96350365 | 0.96460354 |
| 3 | GrimAge acceleration | 0.039 | 0.005 | Intrinsic epigenetic age acceleration | 0.0278951 | 0 | 0.95580442 | 0.96460354 |
| 4 | Hannum age acceleration | 0.027 | 0.003 | DNAm PAI-1 | 6.97E-05 | 0 | 0.948705129 | 0.96460354 |
| 5 | PhenoAge acceleration,telomere length | 0.025 | -0.046,0.141 | PhenoAge acceleration | 0.342379906 | -0.017 | 0.04389561 | 0.263373663 |
| 6 | Intrinsic epigenetic age acceleration | 0.02 | -0.004 | telomere length | 0.59730683 | 0.096 | 0.131686831 | 0.395060494 |
| 7 | Hannum age acceleration,PhenoAge acceleration | 0.007 | 0.047, -0.07 |  |  |  |  |  |
| 8 | GrimAge acceleration,PhenoAge acceleration | 0.005 | 0.045, -0.06 |  |  |  |  |  |
| 9 | GrimAge acceleration,telomere length | 0.005 | 0.01,0.164 |  |  |  |  |  |
| 10 | Intrinsic epigenetic age acceleration,PhenoAge acceleration | 0.004 | 0.031, -0.069 |  |  |  |  |  |
| Other disorders of white blood cells (SNP=144) | |  |  |  |  |  |  |  |
| 1 | telomere length | 0.557 | -0.238 | GrimAge acceleration | 0.142082259 | 0.005 | 0.804719528 | 0.835816418 |
| 2 | GrimAge acceleration | 0.121 | 0.038 | Hannum age acceleration | 0.138577344 | 0.008 | 0.514448555 | 0.835816418 |
| 3 | Hannum age acceleration | 0.115 | 0.055 | Intrinsic epigenetic age acceleration | 0.101223582 | -0.004 | 0.569043096 | 0.835816418 |
| 4 | Intrinsic epigenetic age acceleration | 0.083 | -0.039 | DNAm PAI-1 | 0.000211528 | 0 | 0.745725427 | 0.835816418 |
| 5 | PhenoAge acceleration | 0.065 | 0.012 | PhenoAge acceleration | 0.077324307 | 0.001 | 0.835816418 | 0.835816418 |
| 6 | GrimAge acceleration,telomere length | 0.013 | 0.031, -0.234 | telomere length | 0.600533535 | -0.143 | 0.098790121 | 0.592740726 |
| 7 | Hannum age acceleration,telomere length | 0.013 | 0.056, -0.24 |  |  |  |  |  |
| 8 | Intrinsic epigenetic age acceleration,telomere length | 0.009 | -0.033, -0.225 |  |  |  |  |  |
| 9 | PhenoAge acceleration,telomere length | 0.007 | 0.004, -0.236 |  |  |  |  |  |
| 10 | Hannum age acceleration,Intrinsic epigenetic age acceleration | 0.005 | 0.105, -0.078 |  |  |  |  |  |
| Other and unspecified coagulation defects (SNP=144) | |  |  |  |  |  |  |  |
| 1 | telomere length | 0.532 | 0.354 | GrimAge acceleration | 0.100821859 | -0.001 | 0.924507549 | 0.930706929 |
| 2 | PhenoAge acceleration | 0.189 | -0.11 | Hannum age acceleration | 0.072453981 | 0.001 | 0.930706929 | 0.930706929 |
| 3 | GrimAge acceleration | 0.079 | -0.019 | Intrinsic epigenetic age acceleration | 0.086298649 | -0.005 | 0.723827617 | 0.930706929 |
| 4 | Intrinsic epigenetic age acceleration | 0.067 | -0.059 | DNAm PAI-1 | 0.000161278 | 0 | 0.865713429 | 0.930706929 |
| 5 | Hannum age acceleration | 0.054 | -0.001 | PhenoAge acceleration | 0.228735122 | -0.025 | 0.116088391 | 0.348265173 |
| 6 | PhenoAge acceleration,telomere length | 0.024 | -0.1,0.314 | telomere length | 0.593138861 | 0.209 | 0.102589741 | 0.348265173 |
| 7 | Intrinsic epigenetic age acceleration,telomere length | 0.013 | -0.069,0.38 |  |  |  |  |  |
| 8 | GrimAge acceleration,telomere length | 0.013 | -0.008,0.353 |  |  |  |  |  |
| 9 | Hannum age acceleration,telomere length | 0.009 | -0.002,0.354 |  |  |  |  |  |
| 10 | Hannum age acceleration,PhenoAge acceleration | 0.006 | 0.09, -0.147 |  |  |  |  |  |
| **Validation cohort** | |  |  |  |  |  |  |  |
| Leukaemia (SNP=148) | |  |  |  |  |  |  |  |
| 1 | telomere length | 0.994 | 0.003 | GrimAge acceleration | 7.33E-05 | 0 | 1 | 1 |
| 2 | Hannum age acceleration,telomere length | 0.006 | 0.001,0.003 | Hannum age acceleration | 0.006076573 | 0 | 0.99450055 | 1 |
| 3 | Hannum age acceleration | 0 | 0.001 | Intrinsic epigenetic age acceleration | 5.77E-05 | 0 | 0.99990001 | 1 |
| 4 | GrimAge acceleration,telomere length | 0 | 0,0.003 | DNAm PAI-1 | 7.12E-08 | 0 | 1 | 1 |
| 5 | Intrinsic epigenetic age acceleration,telomere length | 0 | 0,0.003 | PhenoAge acceleration | 4.21E-05 | 0 | 1 | 1 |
| 6 | PhenoAge acceleration,telomere length | 0 | 0,0.003 | telomere length | 0.99966854 | 0.003 | 1.00E-04 | 0.00059994 |
| 7 | Intrinsic epigenetic age acceleration | 0 | 0 |  |  |  |  |  |
| 8 | GrimAge acceleration | 0 | 0 |  |  |  |  |  |
| 9 | PhenoAge acceleration | 0 | 0 |  |  |  |  |  |
| 10 | DNAm PAI-1, telomere length | 0 | 0,0.003 |  |  |  |  |  |
| Lymphoid leukaemia (SNP=142) | |  |  |  |  |  |  |  |
| 1 | telomere length | 0.999 | 0.002 | GrimAge acceleration | 5.43E-05 | 0 | 1 | 1 |
| 2 | Hannum age acceleration,telomere length | 0.001 | 0,0.002 | Hannum age acceleration | 0.000601037 | 0 | 0.99960004 | 1 |
| 3 | Intrinsic epigenetic age acceleration,telomere length | 0 | 0,0.002 | Intrinsic epigenetic age acceleration | 6.38E-05 | 0 | 0.99990001 | 1 |
| 4 | GrimAge acceleration,telomere length | 0 | 0,0.002 | DNAm PAI-1 | 1.03E-07 | 0 | 1 | 1 |
| 5 | PhenoAge acceleration,telomere length | 0 | 0,0.002 | PhenoAge acceleration | 2.73E-05 | 0 | 1 | 1 |
| 6 | Hannum age acceleration | 0 | 0 | telomere length | 0.999993242 | 0.002 | 1.00E-04 | 0.00059994 |
| 7 | Intrinsic epigenetic age acceleration | 0 | 0 |  |  |  |  |  |
| 8 | GrimAge acceleration | 0 | 0 |  |  |  |  |  |
| 9 | GrimAge acceleration,Hannum age acceleration,telomere length | 0 | -0.001,0.001,0.002 |  |  |  |  |  |
| 10 | PhenoAge acceleration | 0 | 0 |  |  |  |  |  |
| Myeloid leukaemia (SNP=136) | |  |  |  |  |  |  |  |
| 1 | Hannum age acceleration | 0.396 | 0 | GrimAge acceleration | 0.244600518 | 0 | 0.212678732 | 0.638036196 |
| 2 | GrimAge acceleration | 0.245 | 0 | Hannum age acceleration | 0.396527757 | 0 | 0.04029597 | 0.241775822 |
| 3 | telomere length | 0.212 | 0 | Intrinsic epigenetic age acceleration | 0.08940226 | 0 | 0.461153885 | 0.885611439 |
| 4 | Intrinsic epigenetic age acceleration | 0.089 | 0 | DNAm PAI-1 | 0.000180293 | 0 | 0.727327267 | 0.885611439 |
| 5 | PhenoAge acceleration | 0.057 | 0 | PhenoAge acceleration | 0.057545961 | 0 | 0.817818218 | 0.885611439 |
| 6 | DNAm PAI-1 | 0 | 0 | telomere length | 0.211973011 | 0 | 0.885611439 | 0.885611439 |
| 7 | Hannum age acceleration,Intrinsic epigenetic age acceleration | 0 | 0,0 |  |  |  |  |  |
| 8 | Hannum age acceleration,telomere length | 0 | 0,0 |  |  |  |  |  |
| 9 | Hannum age acceleration,PhenoAge acceleration | 0 | 0,0 |  |  |  |  |  |
| 10 | GrimAge acceleration,telomere length | 0 | 0,0 |  |  |  |  |  |
| Multiple myeloma (SNP=140) | |  |  |  |  |  |  |  |
| 1 | telomere length | 0.929 | 0.001 | GrimAge acceleration | 0.01714055 | 0 | 0.98760124 | 0.98760124 |
| 2 | Hannum age acceleration | 0.033 | 0 | Hannum age acceleration | 0.033561446 | 0 | 0.95380462 | 0.98760124 |
| 3 | GrimAge acceleration | 0.017 | 0 | Intrinsic epigenetic age acceleration | 0.008841664 | 0 | 0.98580142 | 0.98760124 |
| 4 | PhenoAge acceleration | 0.011 | 0 | DNAm PAI-1 | 4.21E-05 | 0 | 0.96950305 | 0.98760124 |
| 5 | Intrinsic epigenetic age acceleration | 0.009 | 0 | PhenoAge acceleration | 0.010938368 | 0 | 0.9860014 | 0.98760124 |
| 6 | Hannum age acceleration,telomere length | 0 | 0,0.001 | telomere length | 0.929631457 | 0.001 | 0.00919908 | 0.055194481 |
| 7 | DNAm PAI-1 | 0 | 0 |  |  |  |  |  |
| 8 | GrimAge acceleration,telomere length | 0 | 0,0.001 |  |  |  |  |  |
| 9 | PhenoAge acceleration,telomere length | 0 | 0,0.001 |  |  |  |  |  |
| 10 | Intrinsic epigenetic age acceleration,telomere length | 0 | 0,0.001 |  |  |  |  |  |
| Lymphomas (SNP=148) | |  |  |  |  |  |  |  |
| 1 | telomere length | 0.609 | 0.001 | GrimAge acceleration | 0.178661438 | 0 | 0.505249475 | 0.903609639 |
| 2 | GrimAge acceleration | 0.178 | 0.001 | Hannum age acceleration | 0.124802125 | 0 | 0.486551345 | 0.903609639 |
| 3 | Hannum age acceleration | 0.125 | 0 | Intrinsic epigenetic age acceleration | 0.037083847 | 0 | 0.903609639 | 0.903609639 |
| 4 | PhenoAge acceleration | 0.05 | 0 | DNAm PAI-1 | 0.000122268 | 0 | 0.779222078 | 0.903609639 |
| 5 | Intrinsic epigenetic age acceleration | 0.037 | 0 | PhenoAge acceleration | 0.050356577 | 0 | 0.865713429 | 0.903609639 |
| 6 | GrimAge acceleration,telomere length | 0 | 0.001,0.002 | telomere length | 0.609418425 | 0.001 | 0.128887111 | 0.773322668 |
| 7 | DNAm PAI-1 | 0 | 0 |  |  |  |  |  |
| 8 | Hannum age acceleration,telomere length | 0 | 0,0.001 |  |  |  |  |  |
| 9 | PhenoAge acceleration,telomere length | 0 | 0,0.002 |  |  |  |  |  |
| 10 | Intrinsic epigenetic age acceleration,telomere length | 0 | 0,0.001 |  |  |  |  |  |
| Top ten models (combination of risk factors) ranked by the model posterior probability and all risk factors ranked by the marginal inclusion probability based on all genetic variants. Causal effects are log odds ratios for haematologic diseases. Empirical P-values are computed using 200 permutations and adjustment for multiple testing via the Benjamini and Hochberg false discovery rate (FDR) procedure. SNP=single nucleotide polymorphism, PP=posterior probability, MIP =Marginal inclusion probability, DNAm PAI-1= DNA methylation-estimated plasminogen activator inhibitor-1. | | | | | | | | |

**Supplementary Figure 1.** A fishbone diagram to demonstrate which specific haematologic diseases constitute each of the 59 GWAS summary statistics in this study.

**
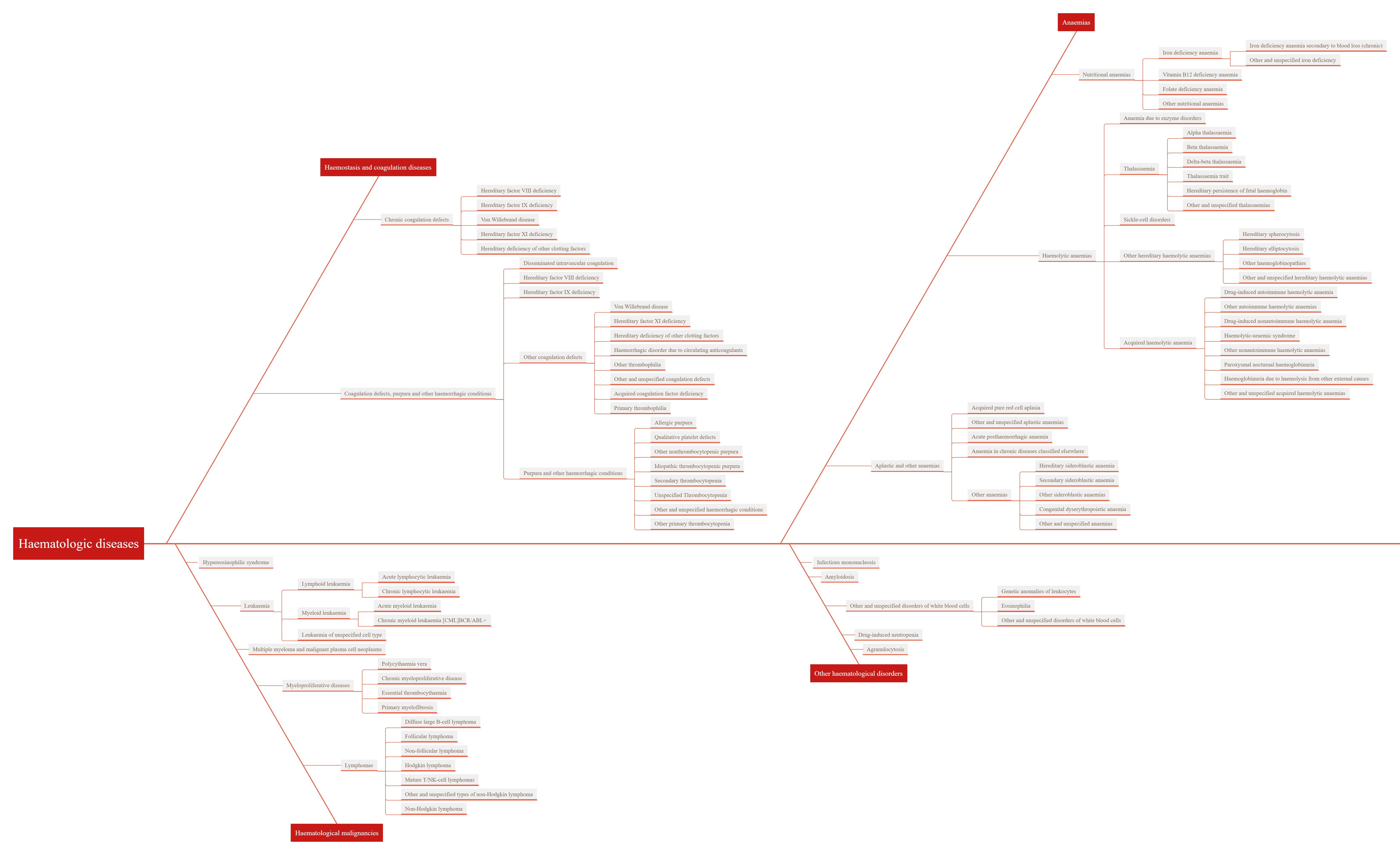
**

Note:

The term “malignant immunoproliferative disease” in FinnGen is defined as “A malignant (clonal) proliferation of B- lymphocytes or T- lymphocytes which involves the lymph nodes, bone marrow and/or extranodal sites”.

**Supplementary Figure 2.** Two-sample single-variable Mendelian randomization results of telomere length and five epigenetic age acceleration on risk of multiple haematologic diseases. (a) The association between genetically increased telomere length and odds of haematologic diseases in the validation cohort. (b) The association between genetically predicted DNA methylation GrimAge acceleration and odds of haematologic diseases in the discovery cohort. (c) The association between genetically predicted DNA methylation Hannum age acceleration and odds of haematologic diseases in the discovery cohort. (d) The association between genetically predicted Intrinsic epigenetic age acceleration and odds of haematologic diseases in the discovery cohort. (e) The association between genetically predicted DNAm PAI-1 levels and odds of haematologic diseases in the discovery cohort. (f) The association between genetically predicted DNA methylation PhenoAge acceleration and odds of haematologic diseases in the discovery cohort. DNAm PAI-1, DNA methylation-estimated plasminogen activator inhibitor-1; nSNP, number of single nucleotide polymorphism; OR, odds ratio; 95%CI, The 95% confidence intervals.

**
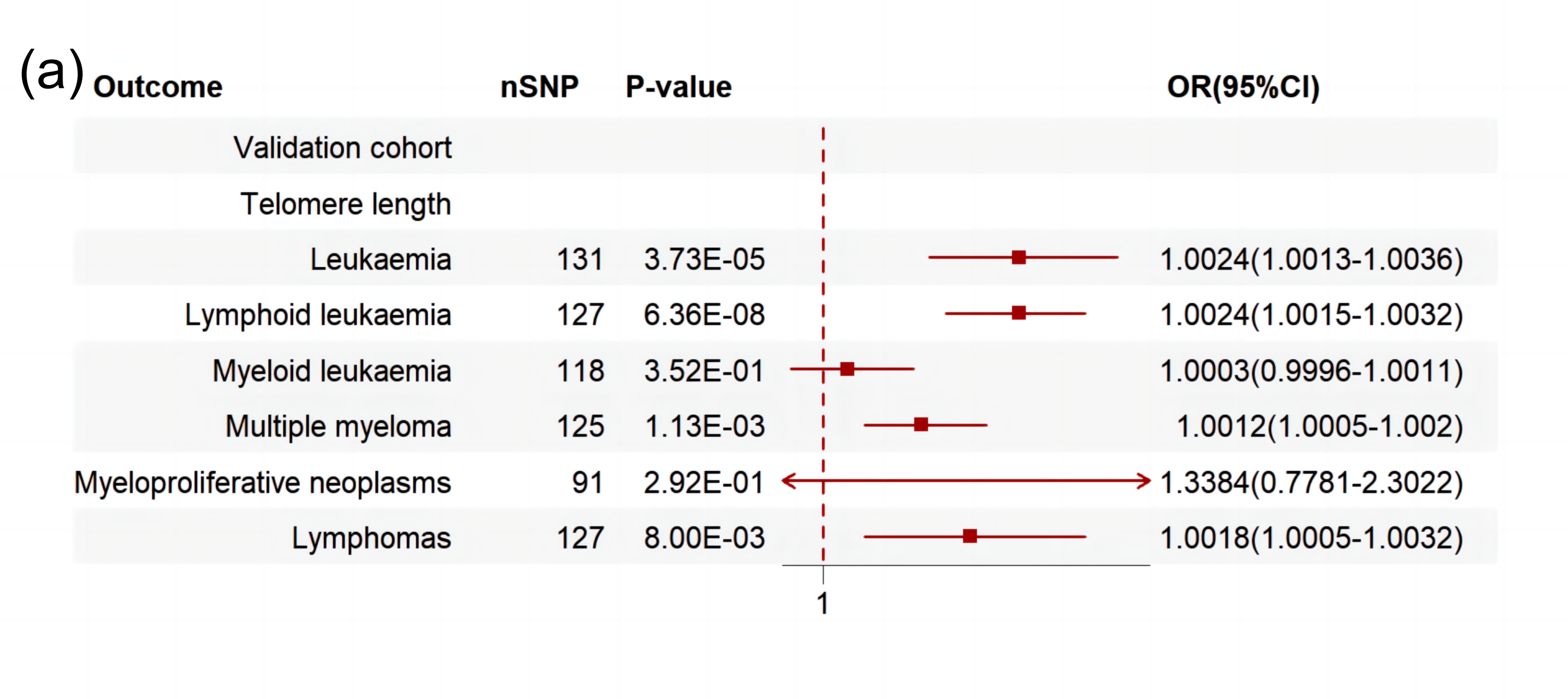

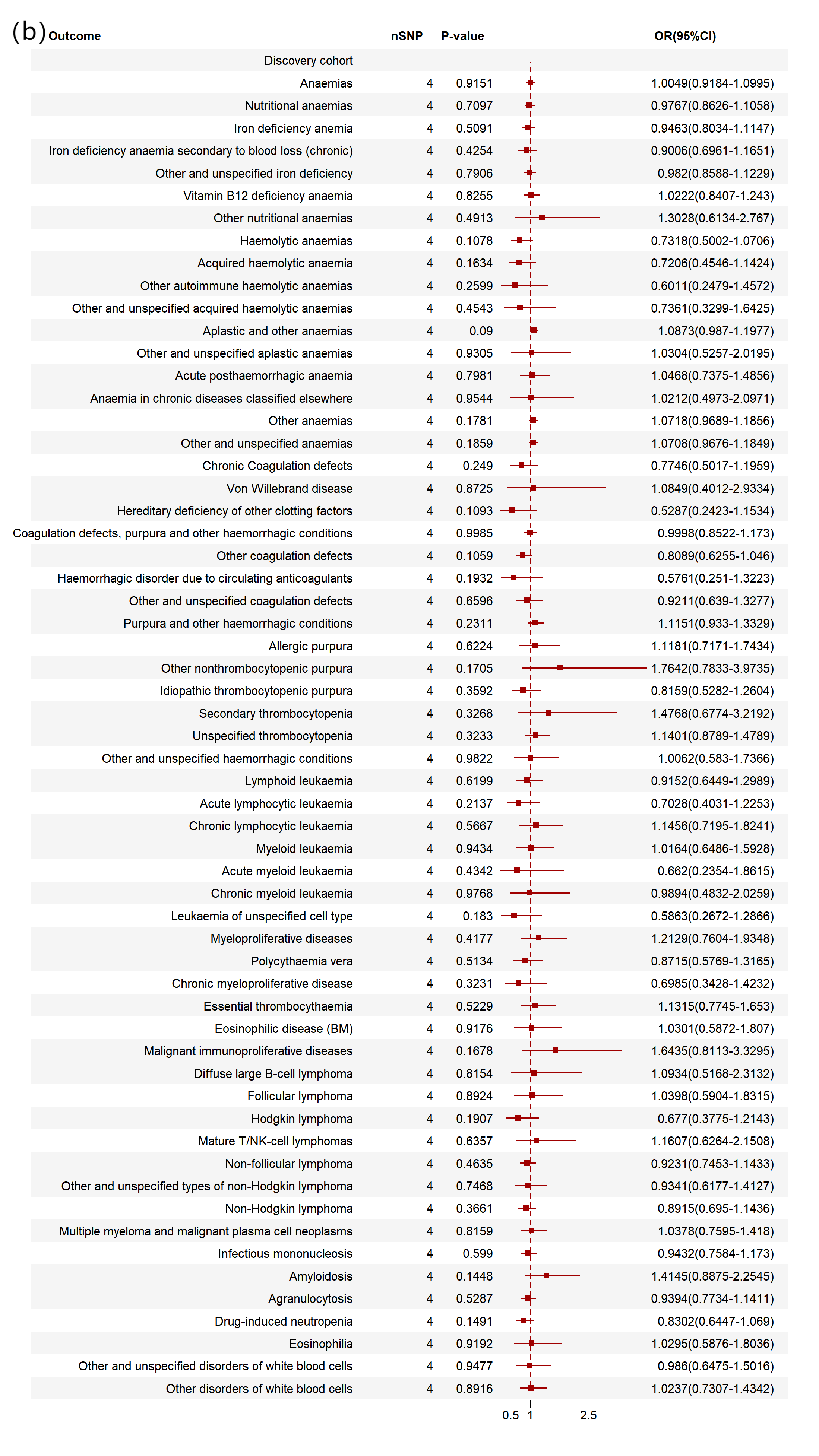

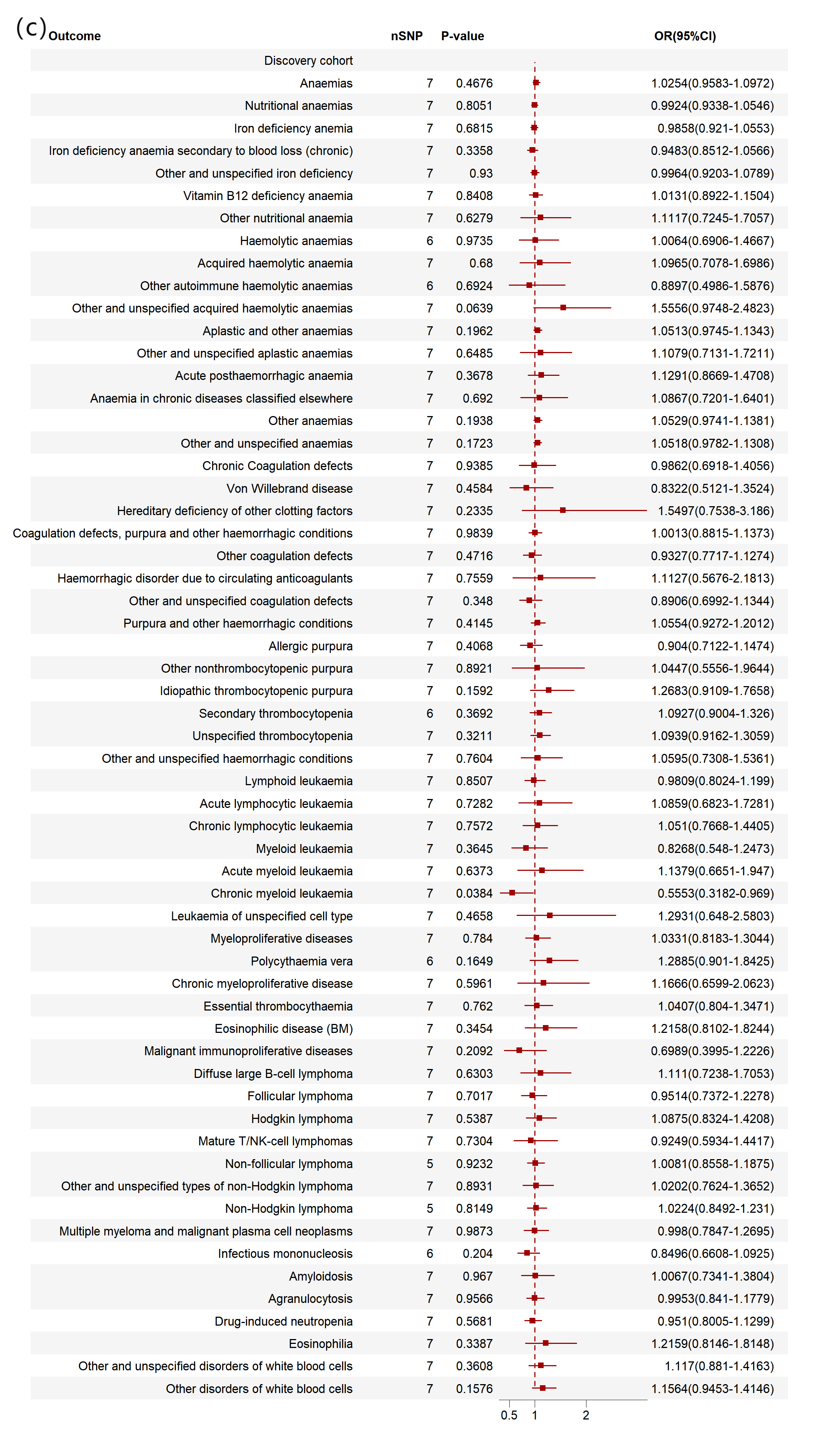
**

**
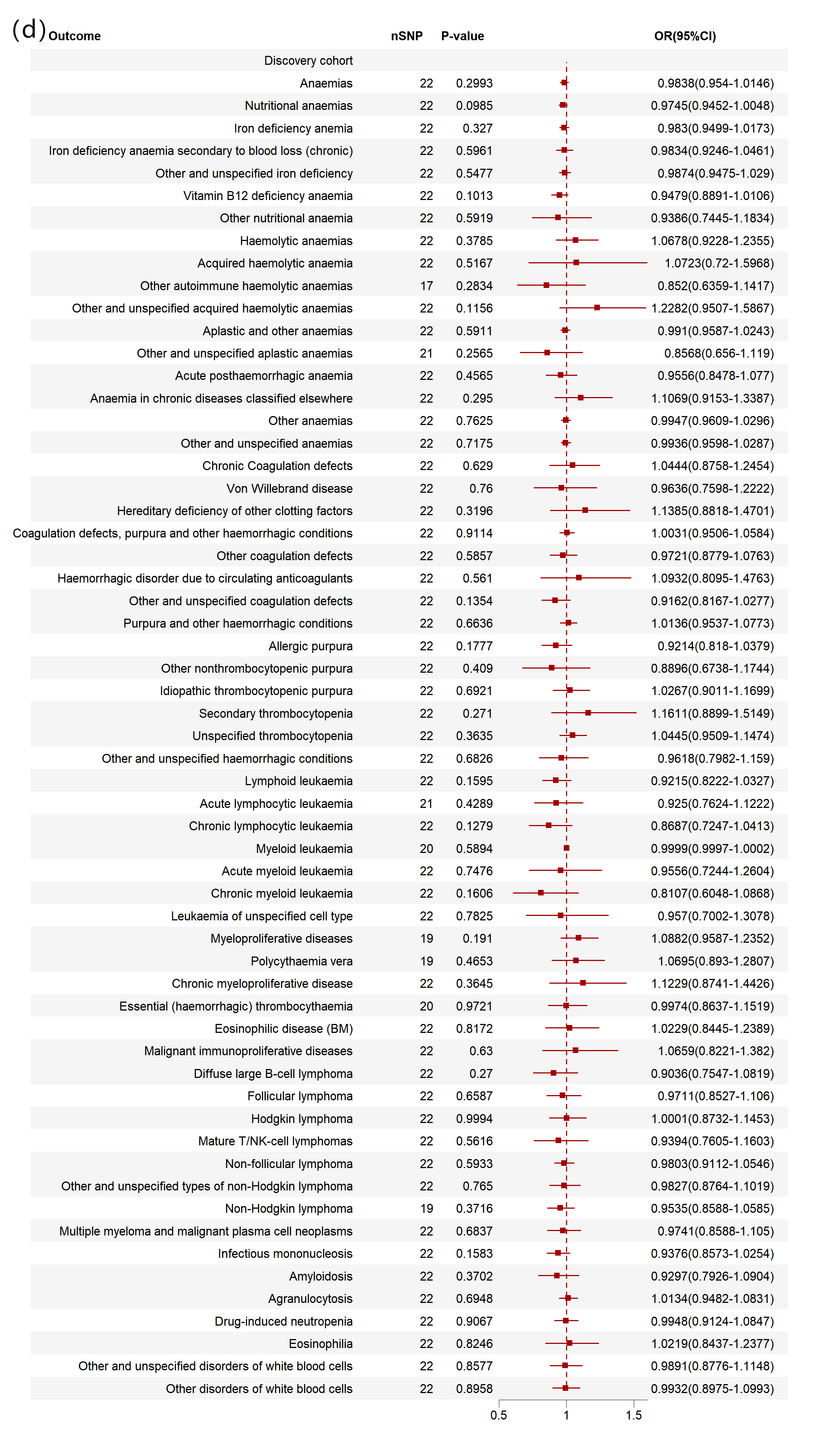

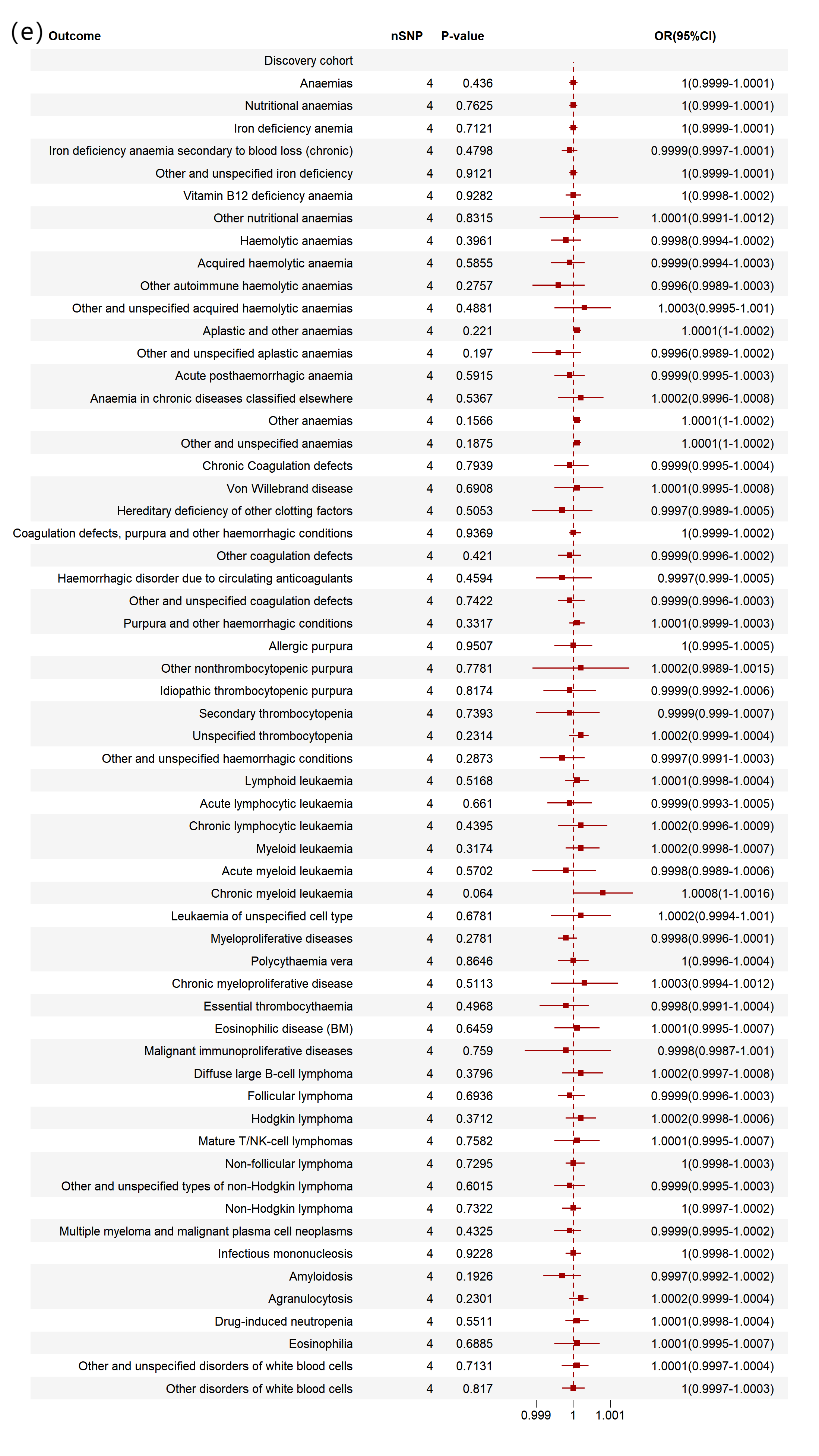

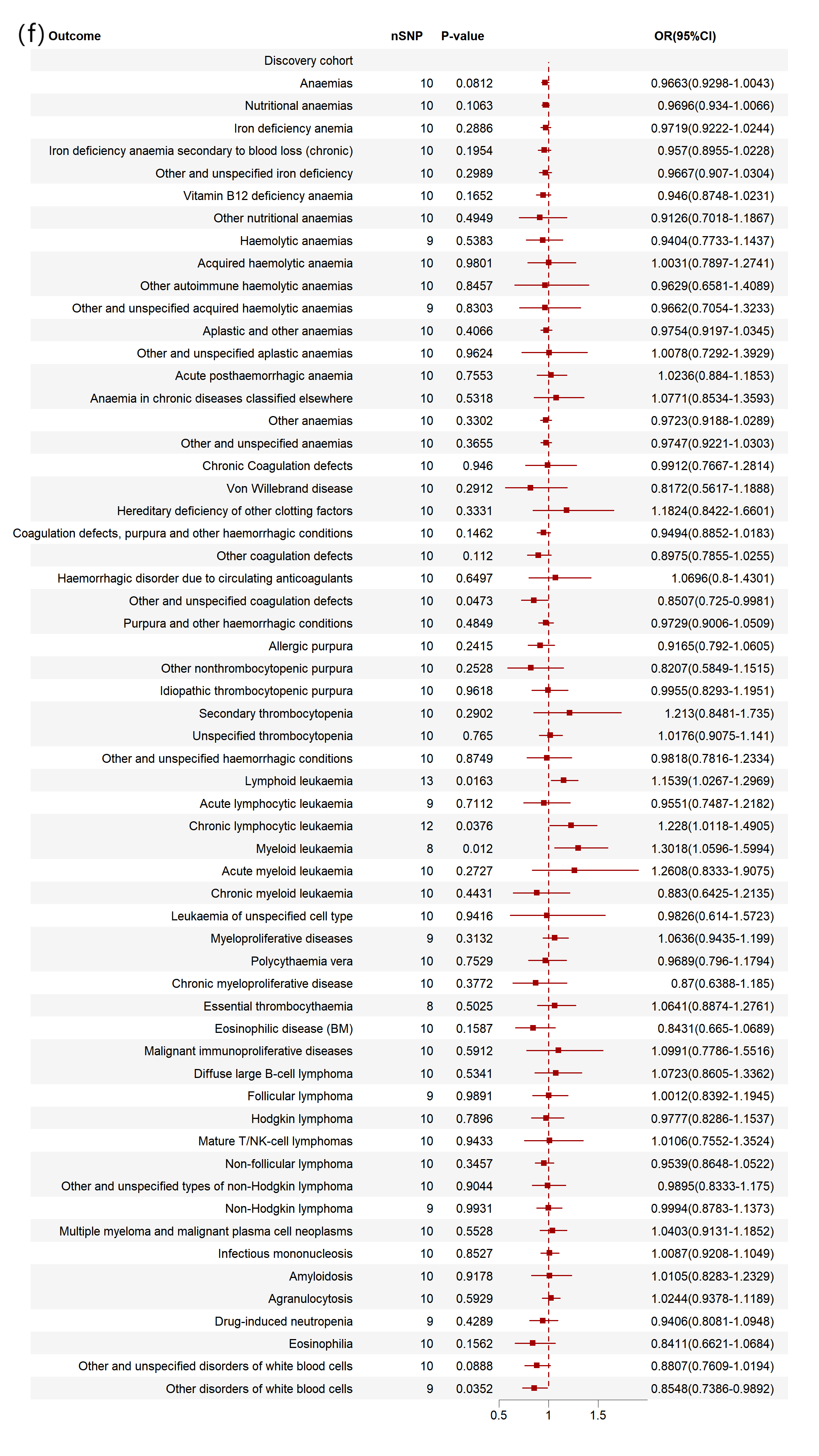
**

**Supplementary Figure 3.** Scatterplot of associations of genetic risk of telomere length on risk of 10 haematological malignancies.

**
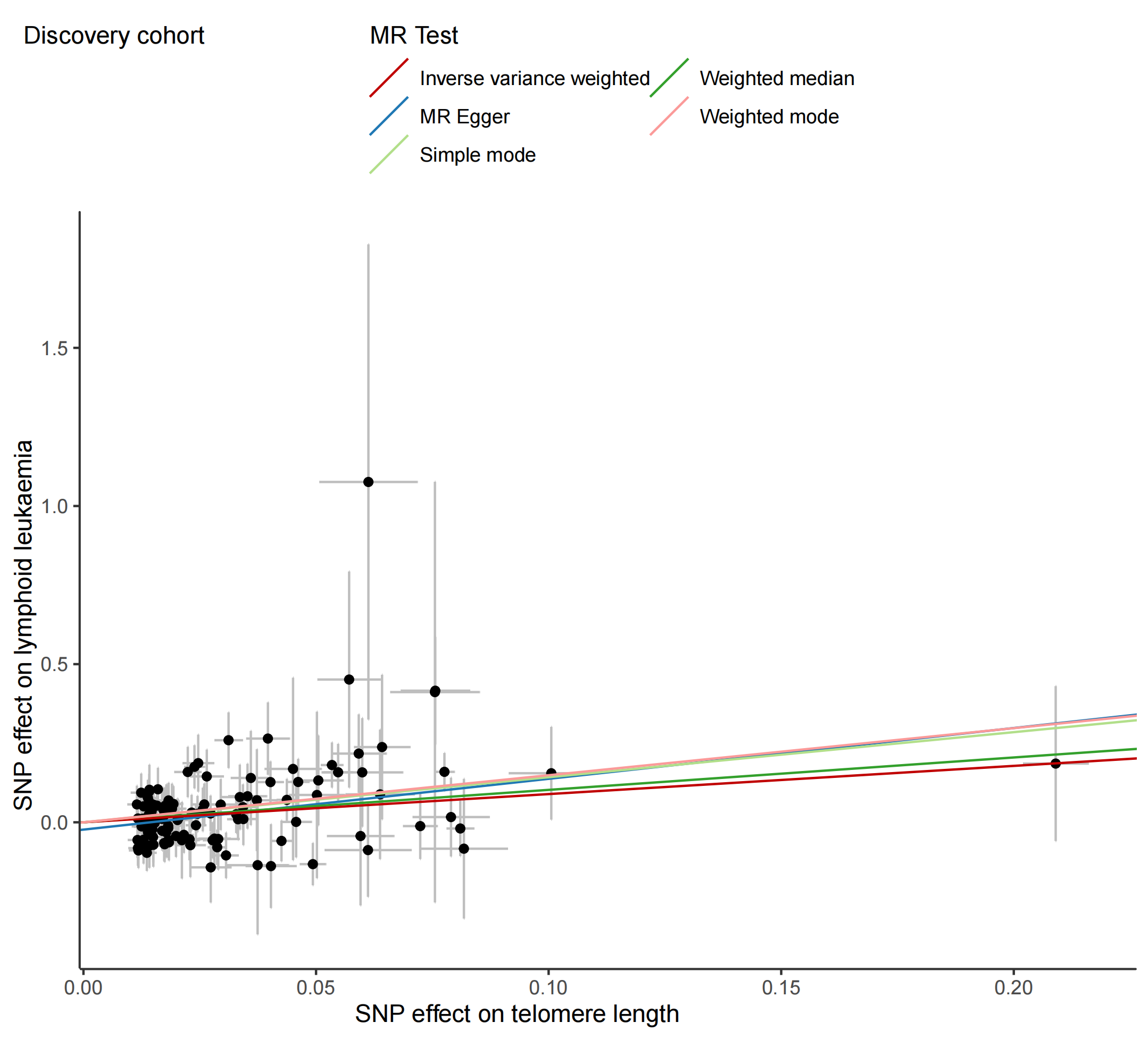

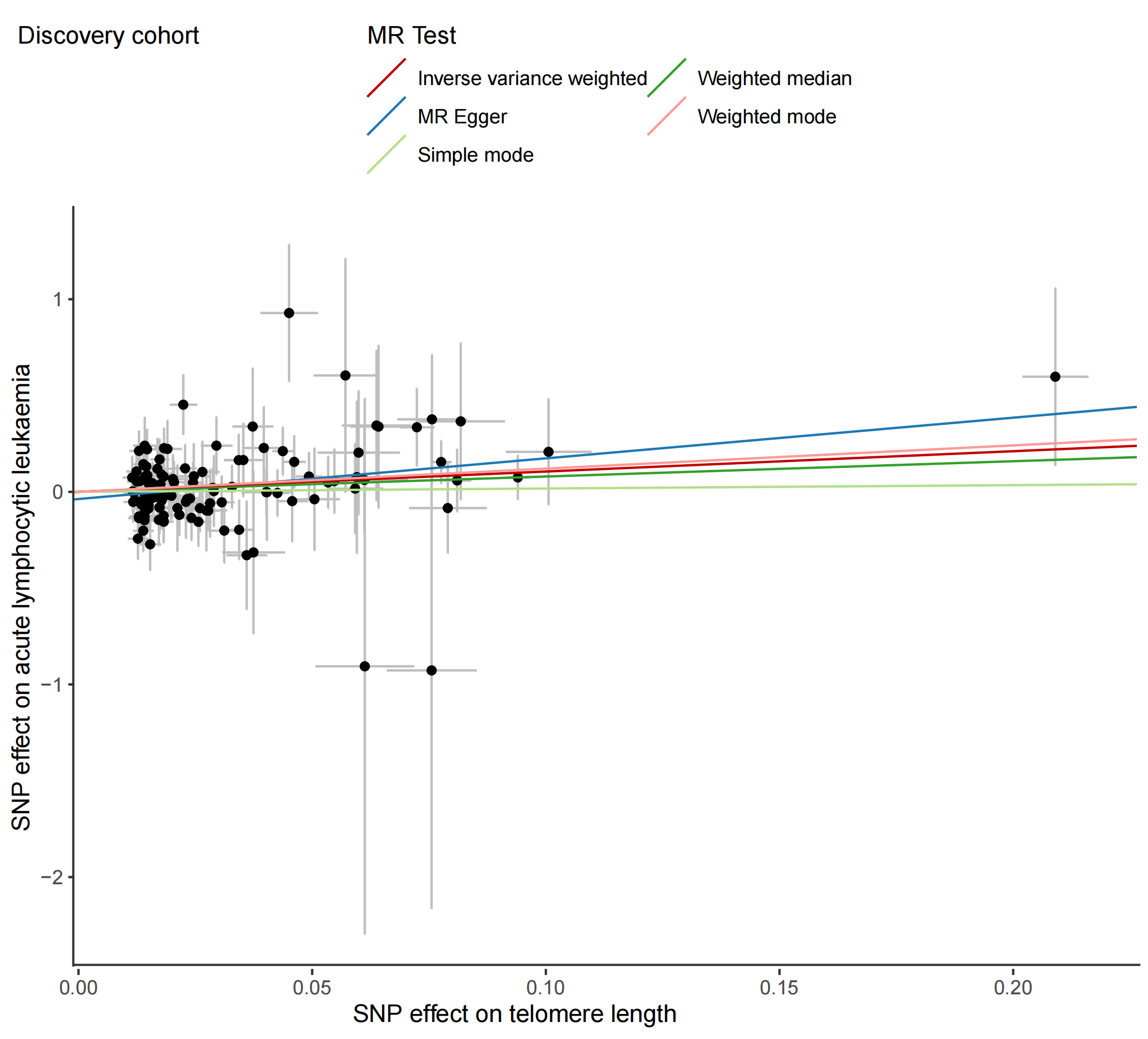
**

**
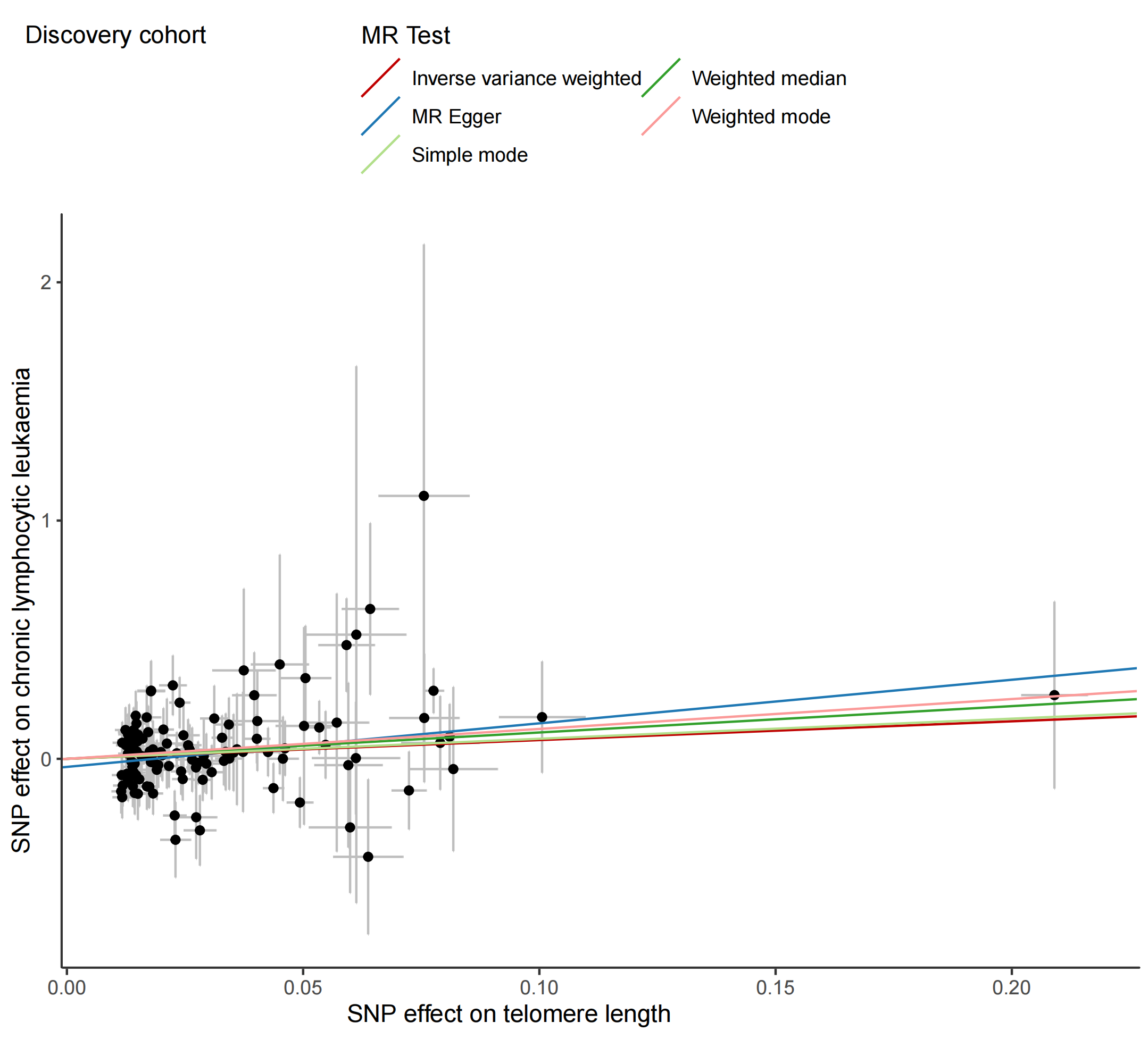

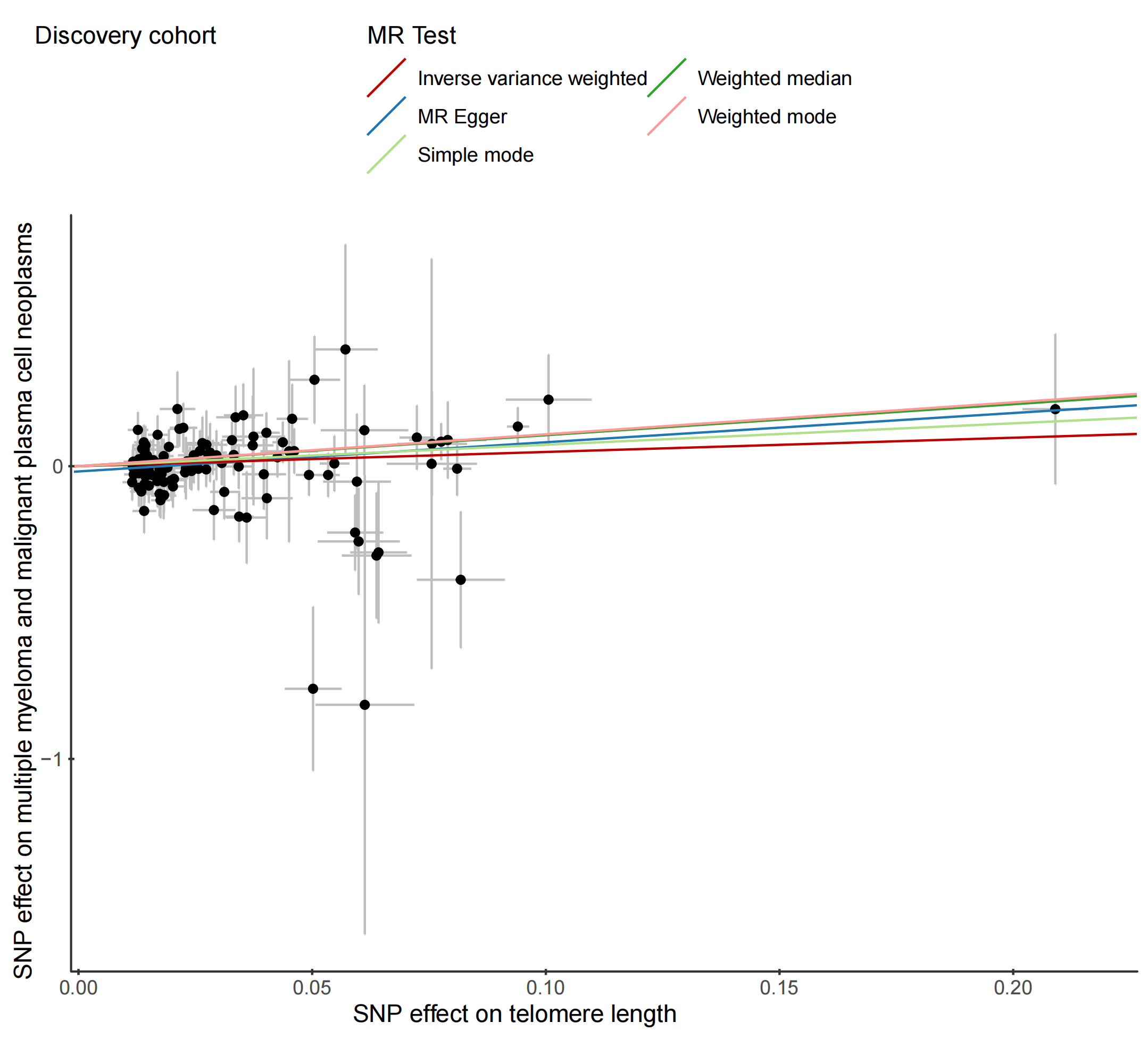
**

**
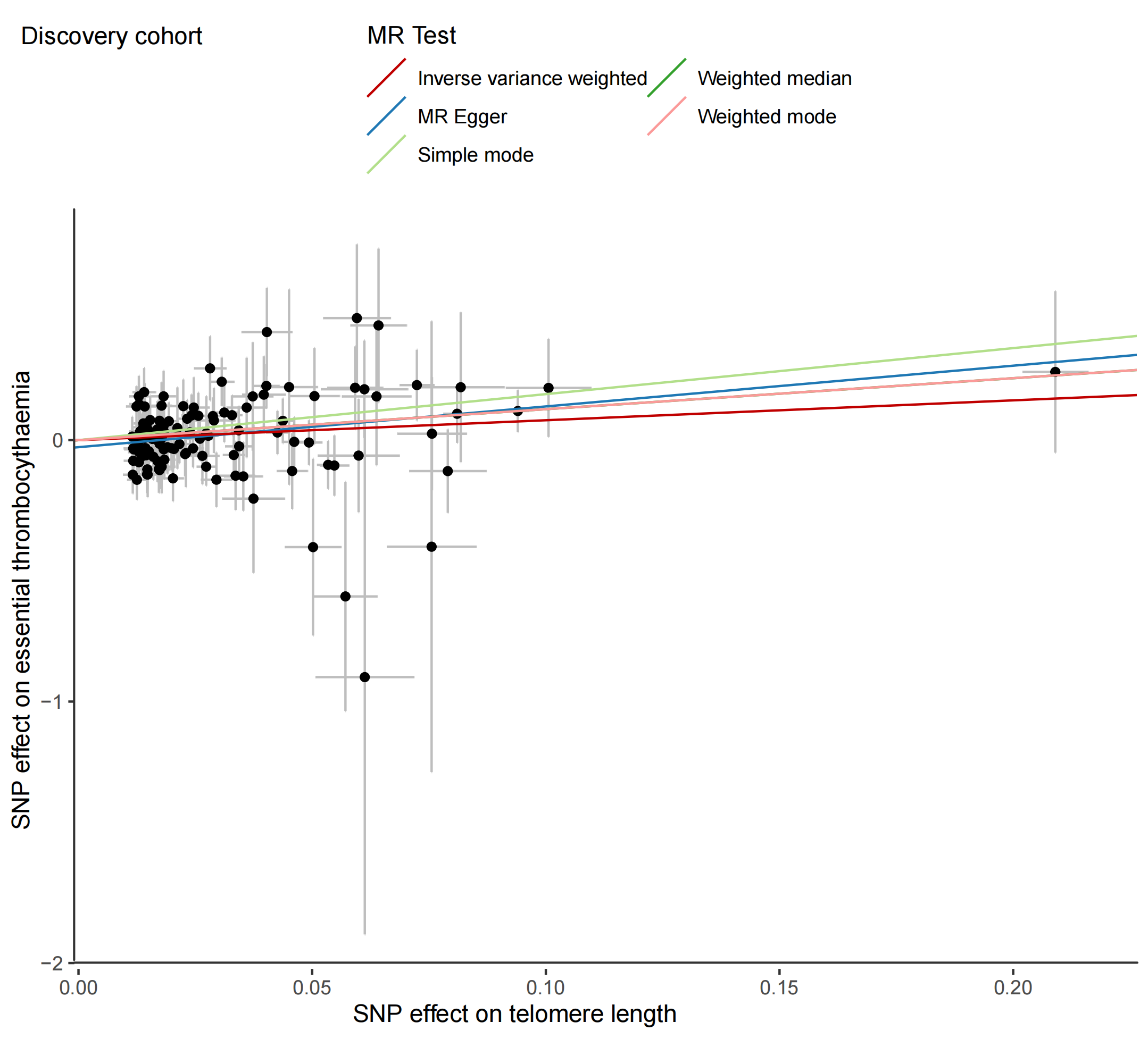

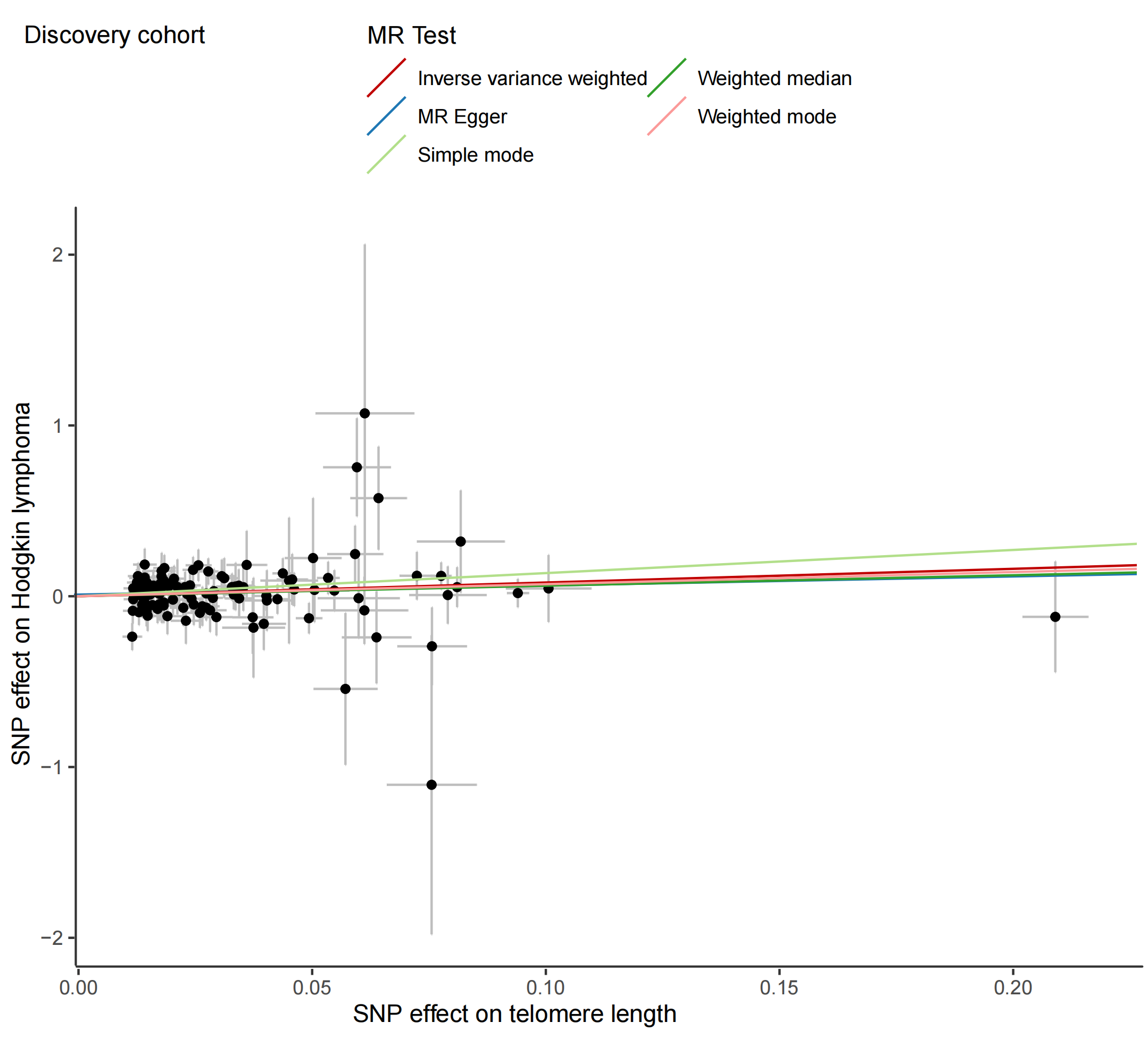
**

**
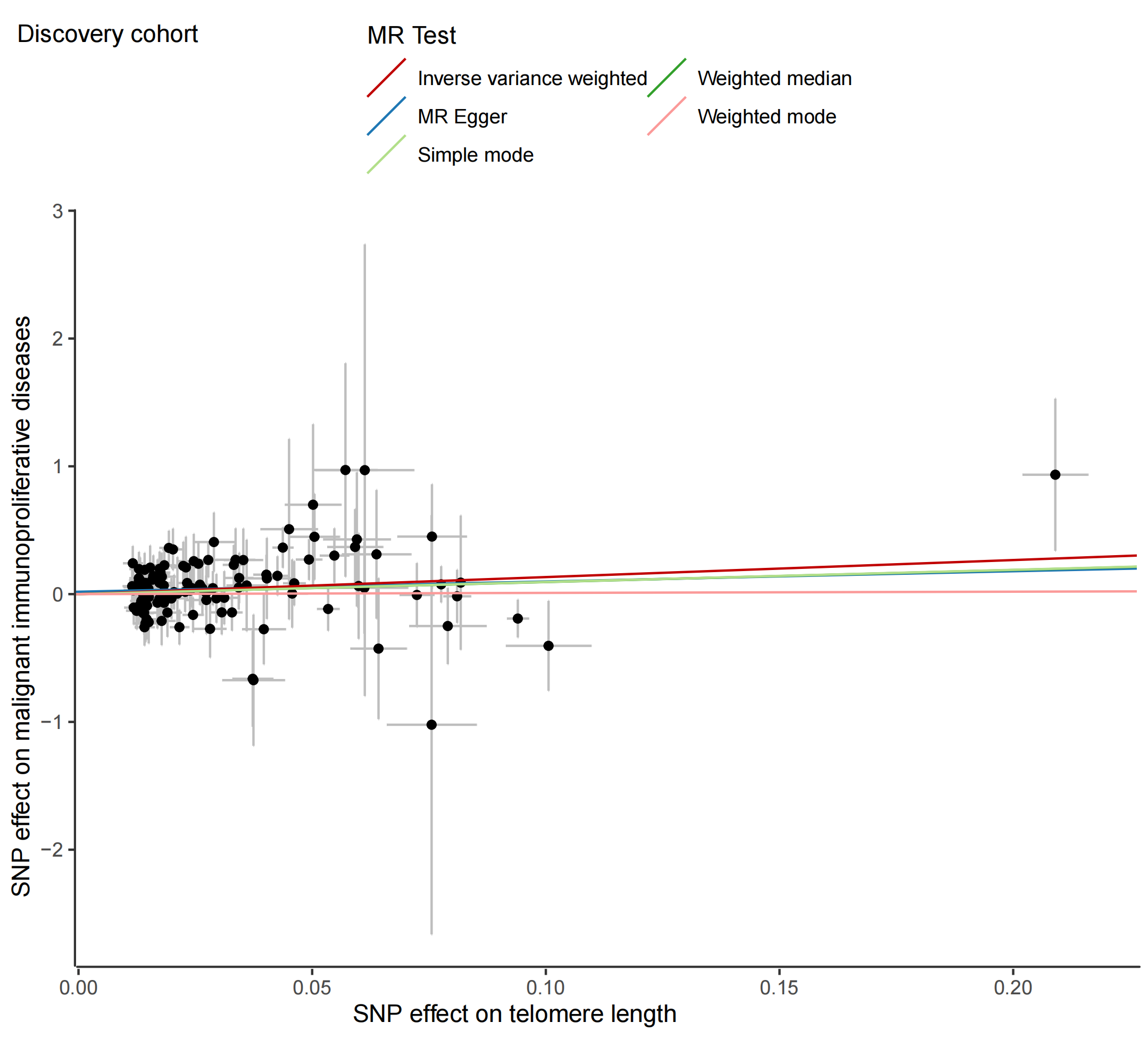

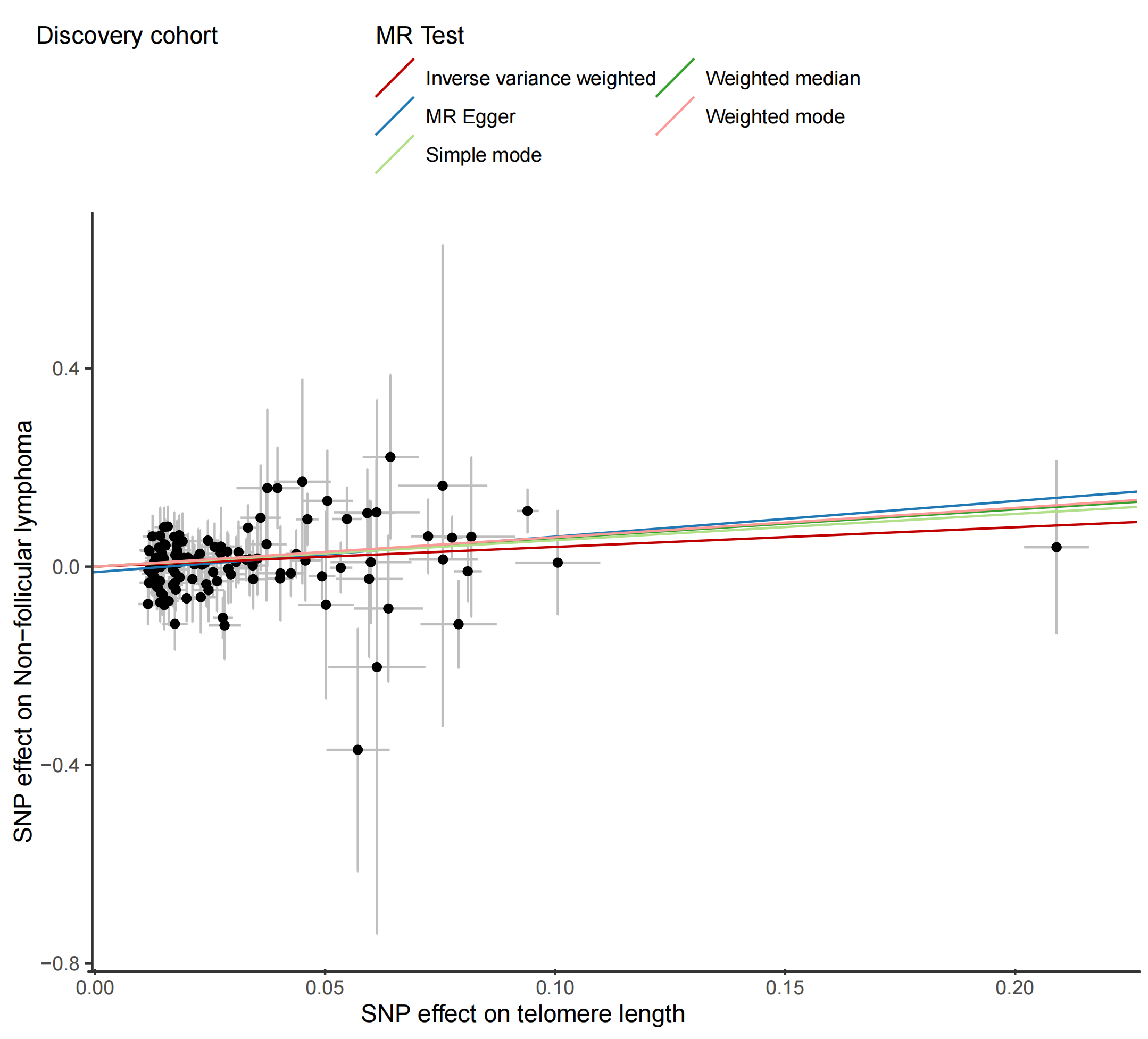
**

**
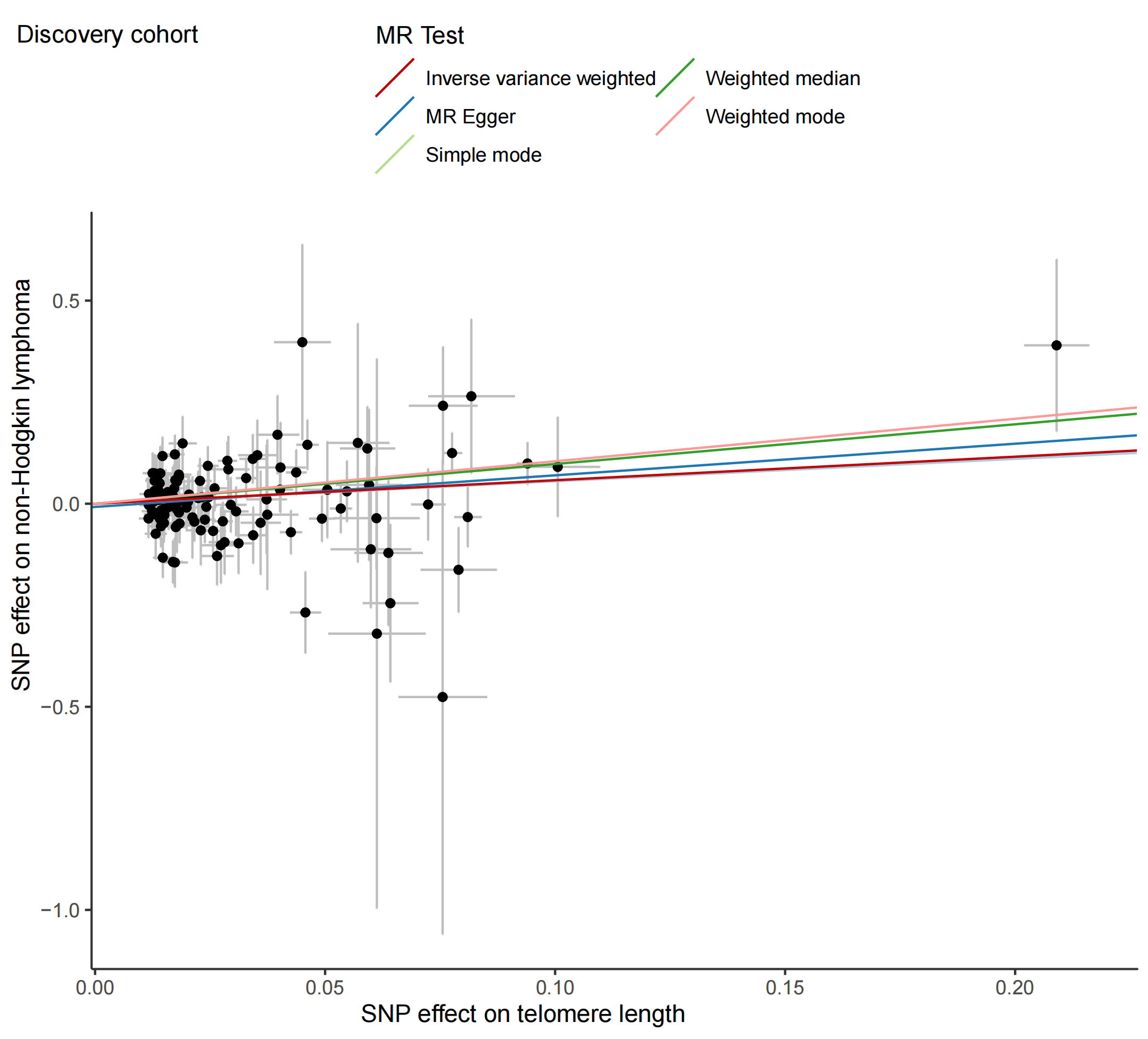

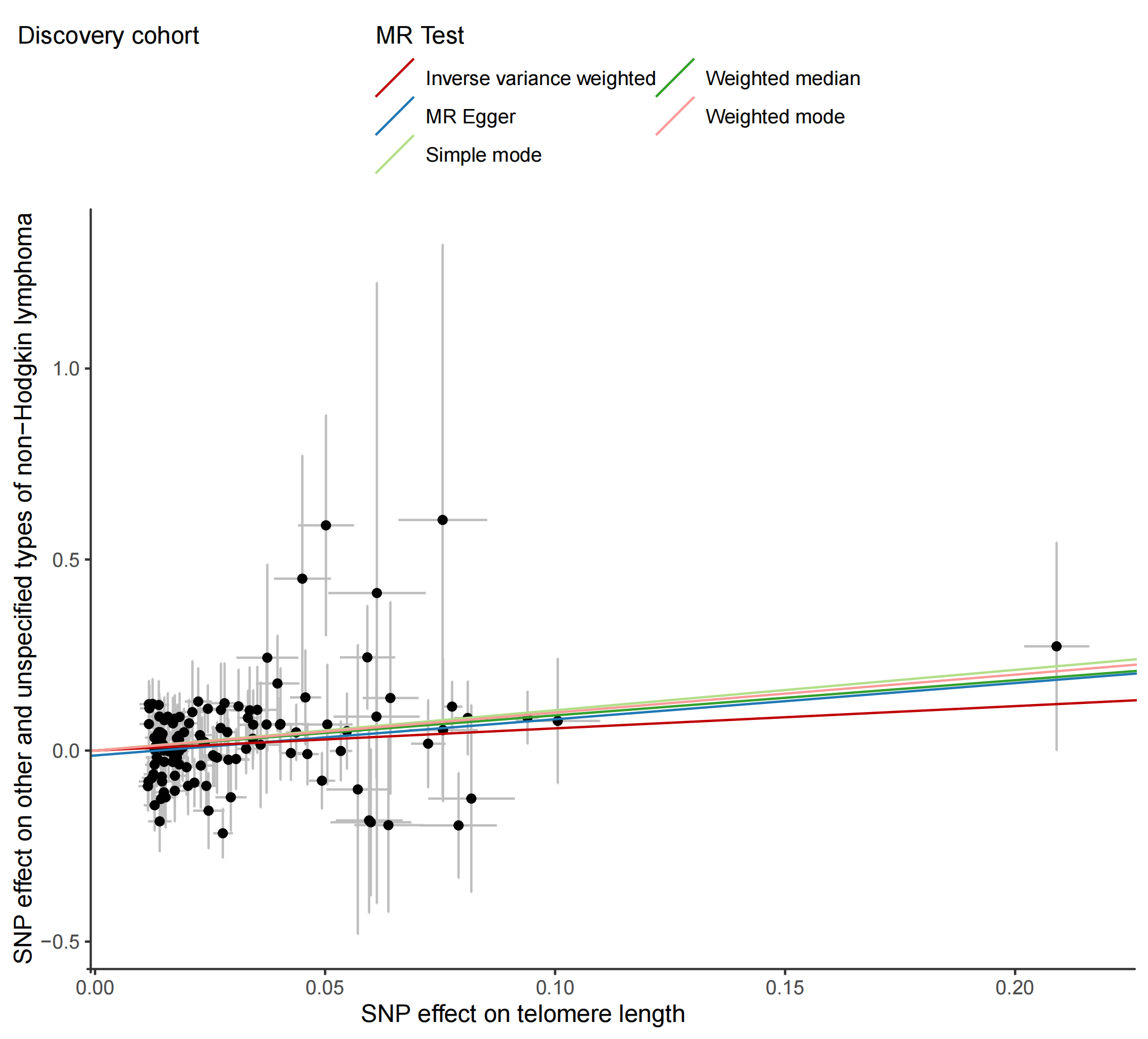
**

**
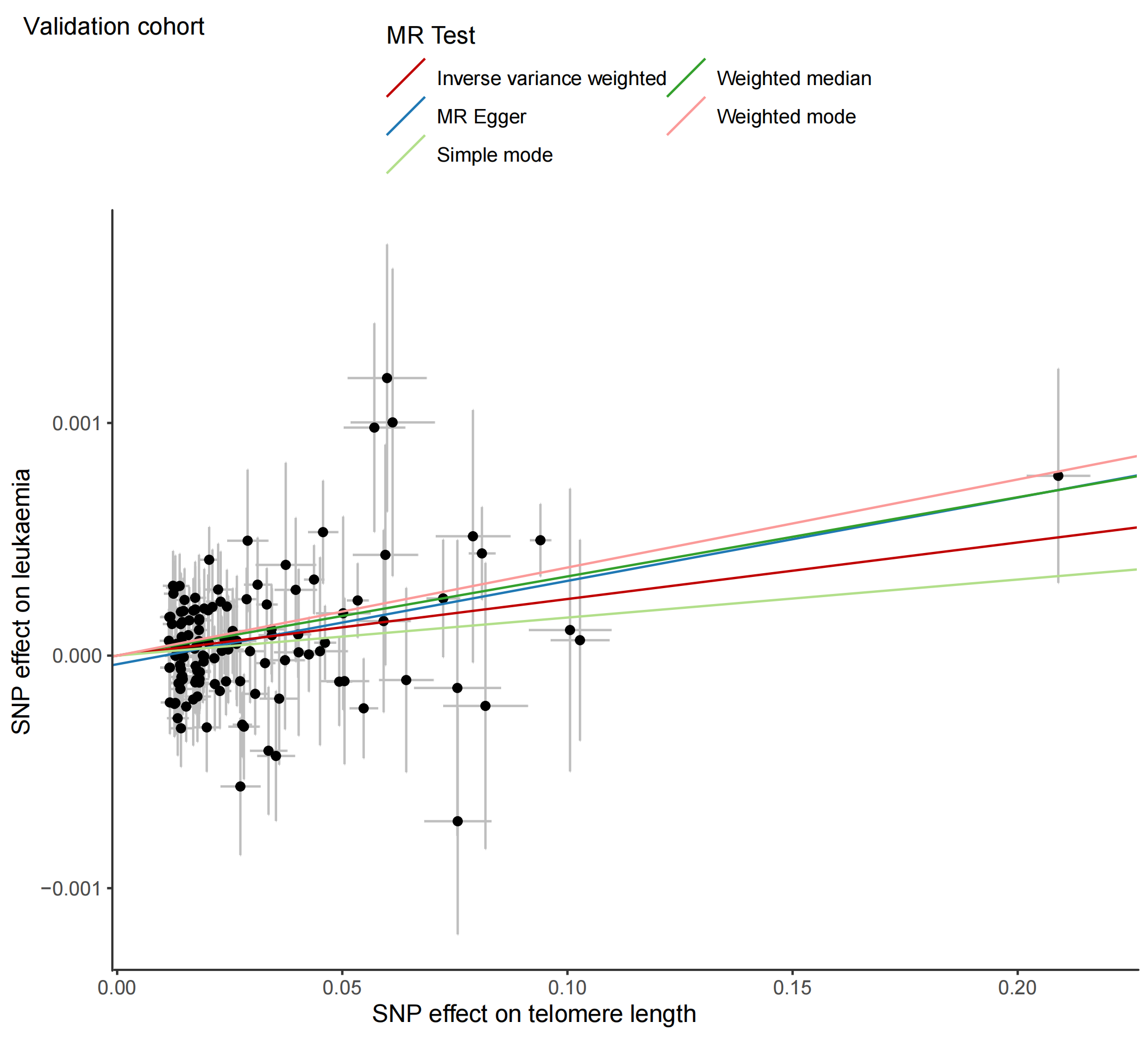

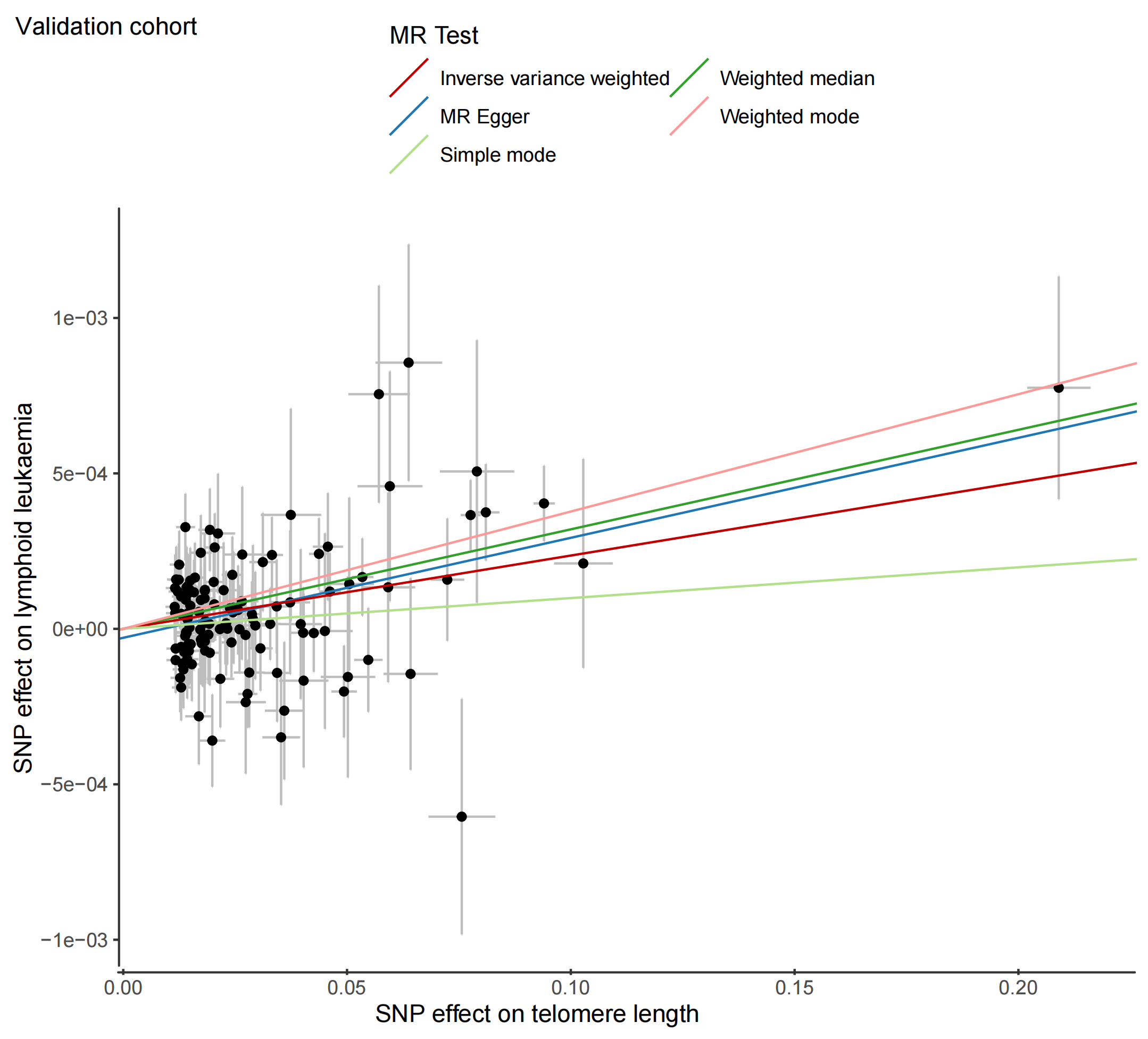
**

**
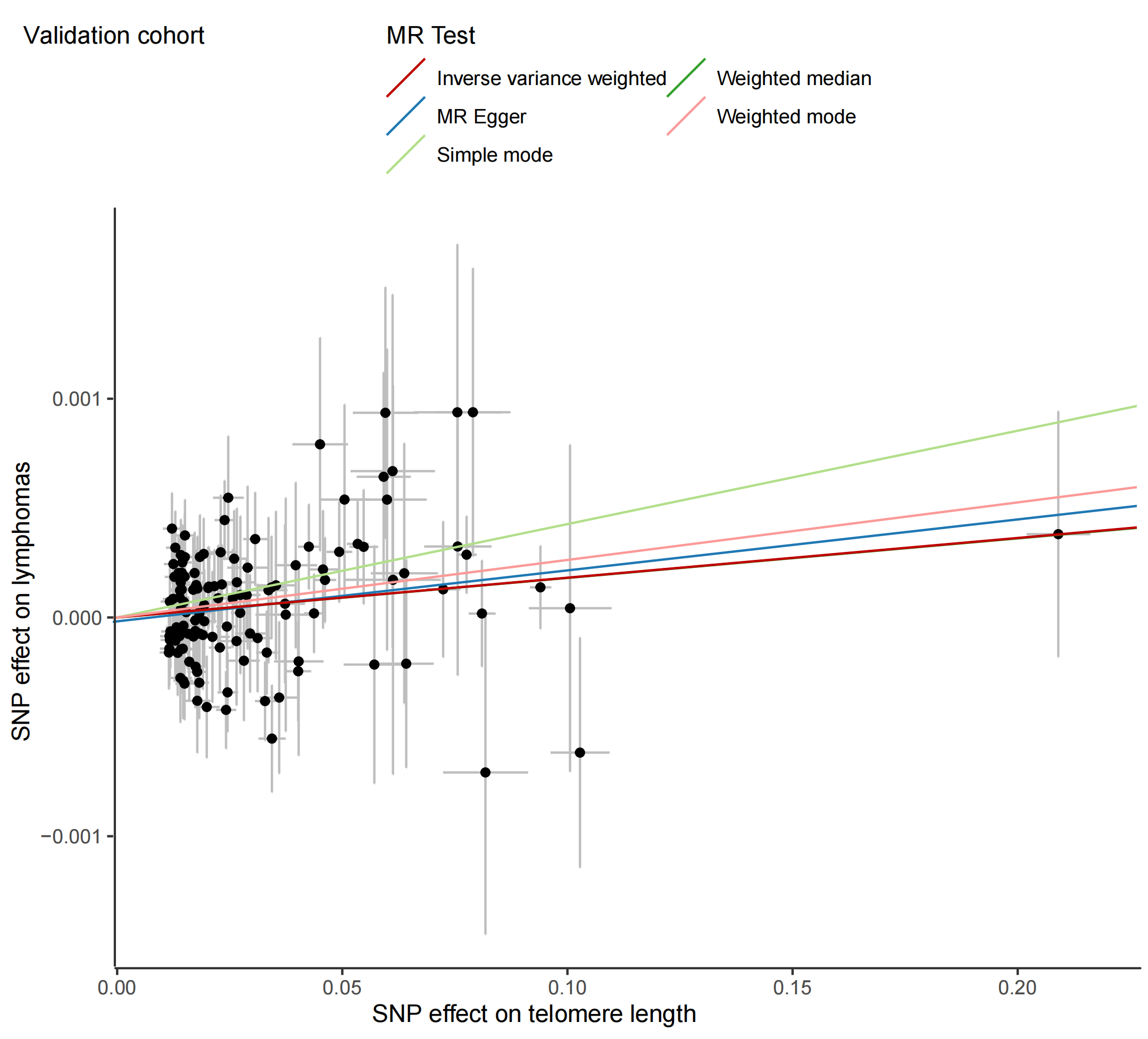

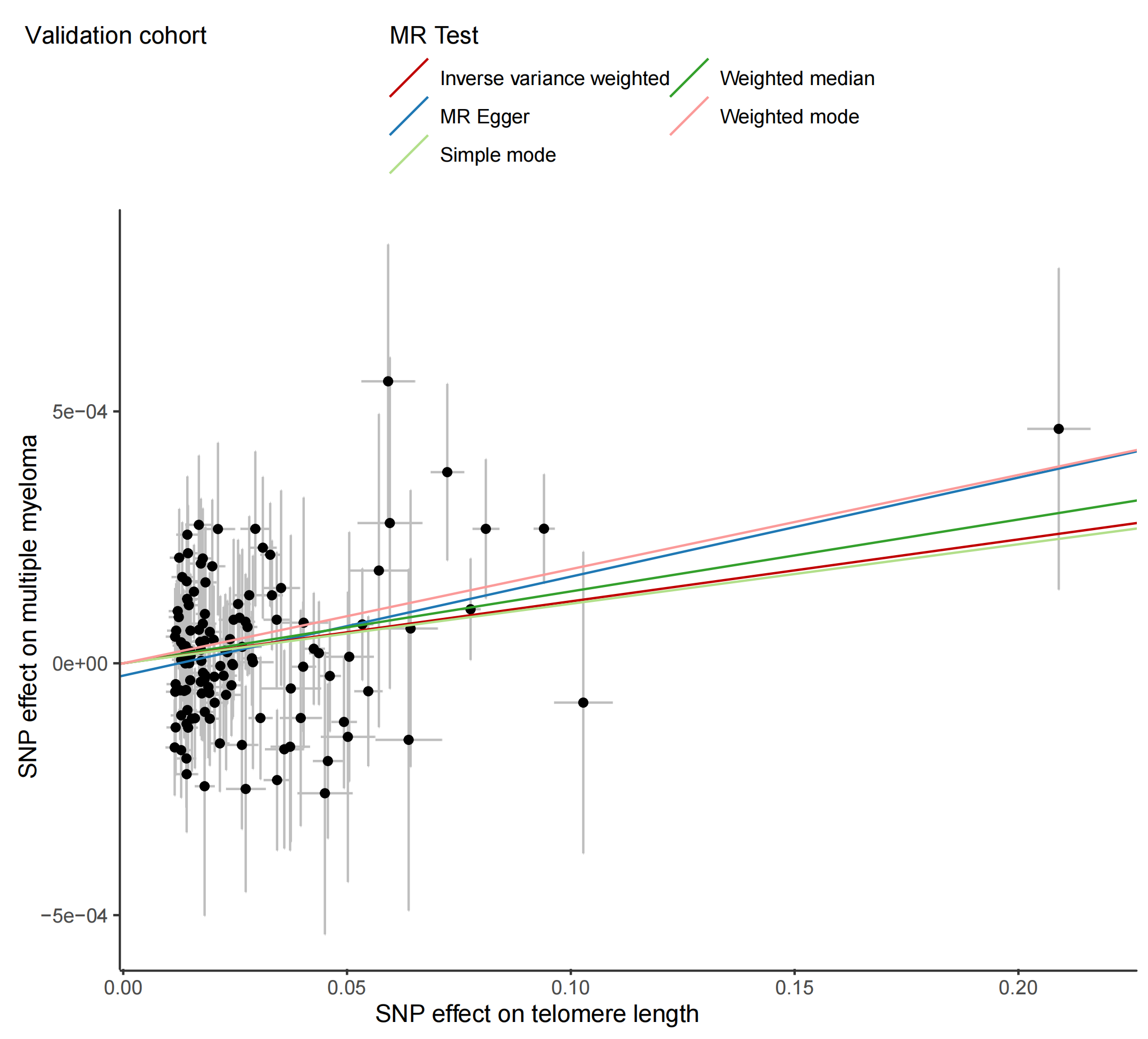
**

**Supplementary Figure 4.** Two-sample single-variable MR results of telomere length and five epigenetic age acceleration on risk of multiple haematologic diseases in the validation cohort.

**
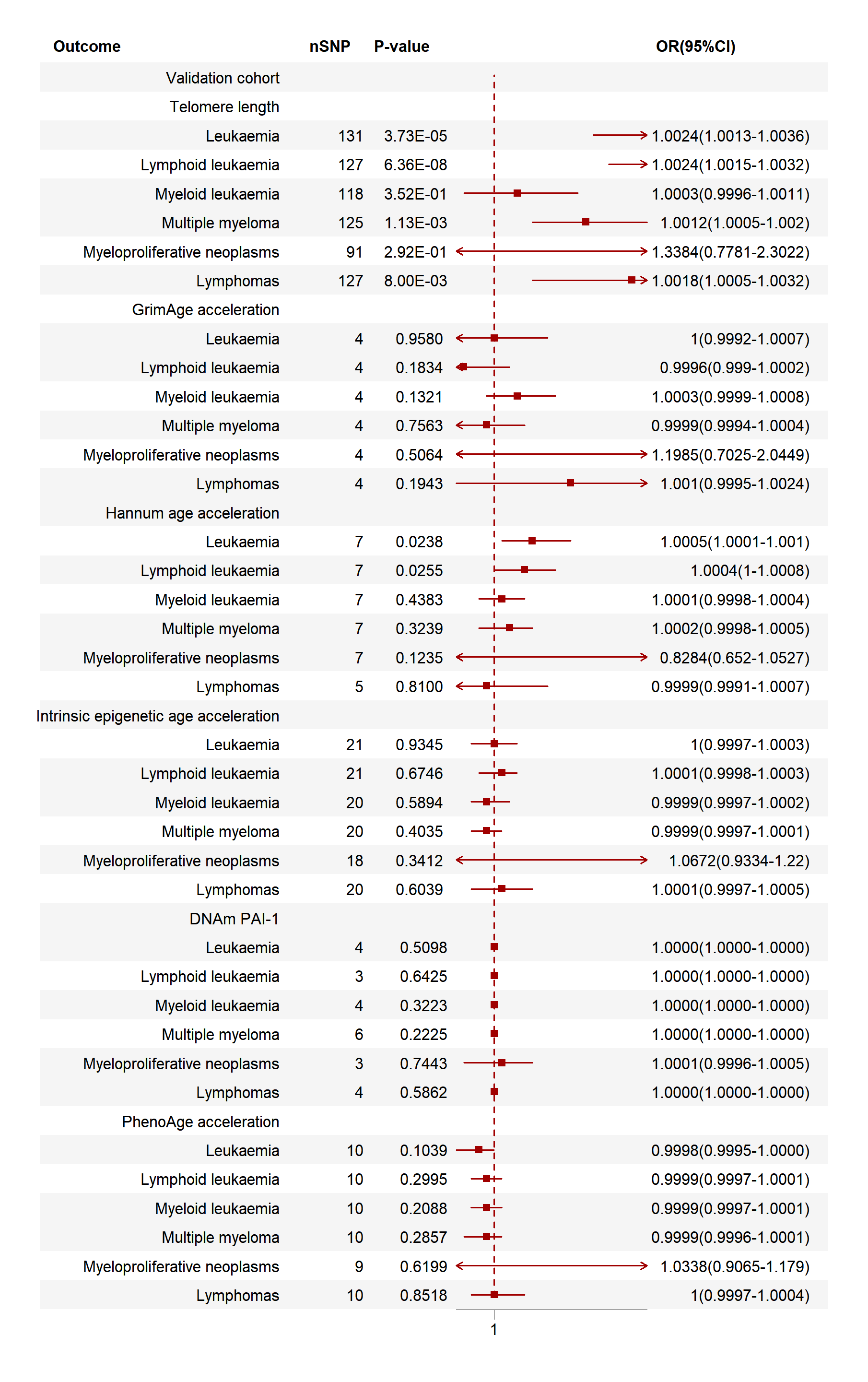
**

**Supplementary Figure 5.** Multivariable Mendelian randomization results of telomere length and five epigenetic age acceleration on risk of multiple haematologic diseases in the validation cohort. DNAm PAI-1, DNA methylation-estimated plasminogen activator inhibitor-1; nSNP, number of single nucleotide polymorphism; OR, odds ratio; 95%CI, The 95% confidence intervals.


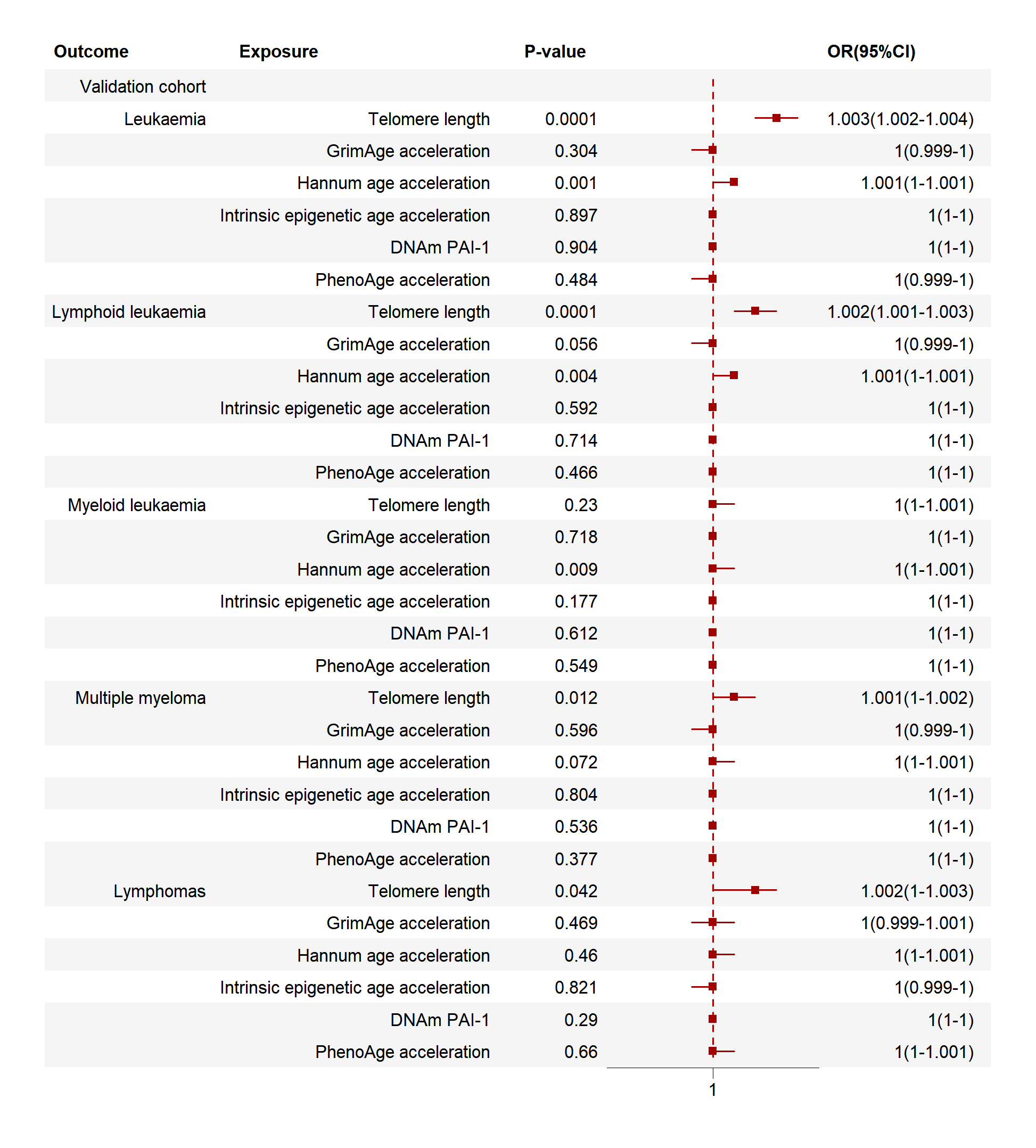


**Supplementary Figure 6 displayed in Supplementary Figure 6.docx**

**Supplementary Method**

1. Genome-wide association studies data sources and instrument selection in MR

Genome-wide association studies data sources and instrument selection in single-variable MR

Telomere length measurements were determined from DNA collected during the baseline assessment, employing a thoroughly validated qPCR assay.(1) These measurements were presented as the ratio of telomere repeat number to a single-copy gene (T/S ratio), and subsequently, log-transformed to approximate a normal distribution.(1) To facilitate comparisons with other datasets, LTL values were z-standardized. A comparable methodology was employed in the original UK Biobank telomere paper.(1) For telomere length analysis, data sources were derived from the UK Biobank, a comprehensive population-based cohort study comprising 472,174 participants from the United Kingdom.(2) To conduct SVMR analyses, we carefully curated the instrumental variables from a pool of 197 independent sentinel variants associated with telomere length, spanning 138 genomic loci.(2) Following a rigorous selection process, we derived a final set of approximately 121 instrumental variables for the SVMR analysis, ensuring the validity of our approach.

For epigenetic age acceleration measures, we acquired summary genetic association estimates from a recent GWAS meta-analysis of biological aging.(3) This particular meta-analysis involved 34,710 participants of European ancestry originating from 28 cohorts.(3) Notably, this analysis identified of 137 loci associated with DNA biomarkers linked to the aging process, including GrimAge(4), HannumAge(5), Intrinsic HorvathAge(6), DNA methylation-estimated plasminogen activator inhibitor-1 (DNAm PAI-1) levels(7), and PhenoAge(7). In certain cases, several SNPs were eliminated to address potential pleiotropic outliers. Specifically, we identified four independent SNPs for GrimAge, seven for HannumAge, 22 for Intrinsic HorvathAge, four for DNAm PAI-1 and 10 for PhenoAge.

Instrument and variable selection in multivariable MR

Multivariable MR serves as an extension of the standard univariable MR, encompassing multiple variables in the analysis.(8) In the MVMR analysis, we incorporated all the risk factors identified from the SVMR analysis, with a particular focus on assessing the significance of telomere length. To satisfy the instrumental SNP independence requirement in the MVMR-BMA, LD clumping was applied to the combination of SNPs of all aging risk factors.(9) Ultimately, the MVMR-BMA model included telomere length, GrimAge, HannumAge, Intrinsic HorvathAge, DNAm PAI-1 levels, and PhenoAge.

2. Statistical analysis

In our analysis, we carefully selected all relevant SNPs identified in each GWAS, ensuring that they met the selection threshold of P＜5×10^-8^ and were uncorrelated (separated by 10,000 kb pairs with R^2^≤0.001).(10) To ensure data consistency, we harmonised the exposure and outcome datasets, while simultaneously excluding palindromic SNPs with intermediate allele frequencies. Additionally, we calculated the F statistics to assess instrument strength.(11)

For the SVMR analysis, we employed the Wald ratio to estimate the effect of exposure on the outcome for each instrumental variable (IV) and subsequently combined their effect sizes using the inverse variance-weighted (IVW) method.(12) However, to ensure the robustness of our estimates, we supplemented the IVW method with MR-Egger, weighted median, simple mode, and weighted mode approaches.(13) The Cochrane's Q value was used to assess heterogeneity, while MR-Egger intercept and MR-PRESSO methods were employed to detect horizontal pleiotropy.(13) To detect potential outliers, we employed leave-one-out cross-validation analysis and MR-PRESSO analysis.(14) Diagnostic plots, including funnel plots, were used to visually illustrate the MR results, enabling the detection of directional pleiotropy through the symmetry of graphical representations.(15)

For MVMR analysis, the IVW model served as the primary method, complemented by the MVMR-Egger and MVMR-LASSO methods.(16) We employed MVMR-BMA, a recently developed method with the unique ability to select the true causal determinants of an outcome from a set of candidate variables, regardless of their correlation.(17) Model posterior probabilities (PP) were calculated for each possible set of variables, quantifying the possibility of specific variables being causal determinants of haematologic disease risk. We also computed the marginal inclusion probability (MIP) for each variable by summing up the models, representing the probability of a causal association between the variable and haematologic diseases.(17) The final step involved obtaining the model-averaged causal effect of each variable on haematologic diseases. Owing to the complexity of obtaining P-values through standard statistical techniques, we alternatively employed permutations (200 times) to derive empirical P-values,(18) and we adjusted for multiple testing using the Benjamini and Hochberg false discovery rate (FDR) procedure.(19) In the MVMR-BMA analysis, Cochran's Q statistic was used to identify outliers, and Cook's distance (Cd) was employed to detect influential observations.(20) A diagnostic evaluation for outliers and influential observations was performed for the top model.

The R packages “TwoSampleMR”, “MRPRESSO”, “MendelianRandomization”, and “mr.raps” were employed for SVMR, MR-PRESSO, and MVMR analyses. All statistical analyses and data visualisation were conducted using R software version 4.0.0.

**CODE AVAILABILITY**

For original data and code, please contact zhanglei1@ihcams.ac.cn.

**References**

1. Codd V, Denniff M, Swinfield C, Warner SC, Papakonstantinou M, Sheth S, et al. Measurement and initial characterization of leukocyte telomere length in 474,074 participants in UK Biobank. Nat Aging. 2022;2(2):170-9.

2. Codd V, Wang Q, Allara E, Musicha C, Kaptoge S, Stoma S, et al. Polygenic basis and biomedical consequences of telomere length variation. Nat Genet. 2021;53(10):1425-33.

3. McCartney DL, Min JL, Richmond RC, Lu AT, Sobczyk MK, Davies G, et al. Genome-wide association studies identify 137 genetic loci for DNA methylation biomarkers of aging. Genome Biol. 2021;22(1):194.

4. Lu AT, Quach A, Wilson JG, Reiner AP, Aviv A, Raj K, et al. DNA methylation GrimAge strongly predicts lifespan and healthspan. Aging (Albany NY). 2019;11(2):303-27.

5. Hannum G, Guinney J, Zhao L, Zhang L, Hughes G, Sadda S, et al. Genome-wide methylation profiles reveal quantitative views of human aging rates. Mol Cell. 2013;49(2):359-67.

6. Horvath S. DNA methylation age of human tissues and cell types. Genome Biol. 2013;14(10):R115.

7. Levine ME, Lu AT, Quach A, Chen BH, Assimes TL, Bandinelli S, et al. An epigenetic biomarker of aging for lifespan and healthspan. Aging (Albany NY). 2018;10(4):573-91.

8. Rosoff DB, Smith GD, Lohoff FW. Prescription Opioid Use and Risk for Major Depressive Disorder and Anxiety and Stress-Related Disorders: A Multivariable Mendelian Randomization Analysis. JAMA Psychiatry. 2021;78(2):151-60.

9. Zhang Y, Zhao M, Guo P, Wang Y, Liu L, Zhao J, et al. Mendelian randomisation highlights hypothyroidism as a causal determinant of idiopathic pulmonary fibrosis. EBioMedicine. 2021;73:103669.

10. Telomeres Mendelian Randomization C, Haycock PC, Burgess S, Nounu A, Zheng J, Okoli GN, et al. Association Between Telomere Length and Risk of Cancer and Non-Neoplastic Diseases: A Mendelian Randomization Study. JAMA Oncol. 2017;3(5):636-51.

11. Burgess S, Small DS, Thompson SG. A review of instrumental variable estimators for Mendelian randomization. Stat Methods Med Res. 2017;26(5):2333-55.

12. Burgess S, Butterworth A, Thompson SG. Mendelian randomization analysis with multiple genetic variants using summarized data. Genet Epidemiol. 2013;37(7):658-65.

13. Verbanck M, Chen CY, Neale B, Do R. Detection of widespread horizontal pleiotropy in causal relationships inferred from Mendelian randomization between complex traits and diseases. Nat Genet. 2018;50(5):693-8.

14. Noyce AJ, Kia DA, Hemani G, Nicolas A, Price TR, De Pablo-Fernandez E, et al. Estimating the causal influence of body mass index on risk of Parkinson disease: A Mendelian randomisation study. PLoS Med. 2017;14(6):e1002314.

15. Chen L, Yang H, Li H, He C, Yang L, Lv G. Insights into modifiable risk factors of cholelithiasis: A Mendelian randomization study. Hepatology. 2022;75(4):785-96.

16. Slob EAW, Burgess S. A comparison of robust Mendelian randomization methods using summary data. Genet Epidemiol. 2020;44(4):313-29.

17. Zuber V, Colijn JM, Klaver C, Burgess S. Selecting likely causal risk factors from high-throughput experiments using multivariable Mendelian randomization. Nat Commun. 2020;11(1):29.

18. Zuber V, Gill D, Ala-Korpela M, Langenberg C, Butterworth A, Bottolo L, et al. High-throughput multivariable Mendelian randomization analysis prioritizes apolipoprotein B as key lipid risk factor for coronary artery disease. Int J Epidemiol. 2021;50(3):893-901.

19. Chen X. False discovery rate control for multiple testing based on discrete p-values. Biom J. 2020;62(4):1060-79.

20. Herrig IM, Boer SI, Brennholt N, Manz W. Development of multiple linear regression models as predictive tools for fecal indicator concentrations in a stretch of the lower Lahn River, Germany. Water Res. 2015;85:148-57.
