# Supplementary material for: Genetically determined telomere length and risk for haematologic diseases: results from large prospective cohorts and Mendelian Randomization analysis
Source: Blood Cancer J. 2024 Mar 18;14(1):48. doi: 10.1038/s41408-024-01035-5 (PMC10948832; doi:10.1038/s41408-024-01035-5)

**Supplementary Figure 6.** Model diagnostics in multivariable MR based on BMA. The predicted associations with haematologic diseases based on the model including telomere length (x-axis) are plotted against the observed associations with haematologic diseases (y-axis). These are the top models when keeping outliers and influential genetic variants in the analysis. (a) Cook's distance for the influential points; (b) the q-statistic for outliers. Any genetic variant with q value larger than threshold or Cook's distance larger than the median of the relevant F-distribution is marked by a label indicating the gene region.

(1)

The predicted associations with leukaemia (validation cohort) based on the model including telomere length (x-axis) are plotted against the observed associations with leukaemia (y-axis). These are the top models when keeping outliers and influential genetic variants in the analysis.

(a) Cook's distance for the influential points;


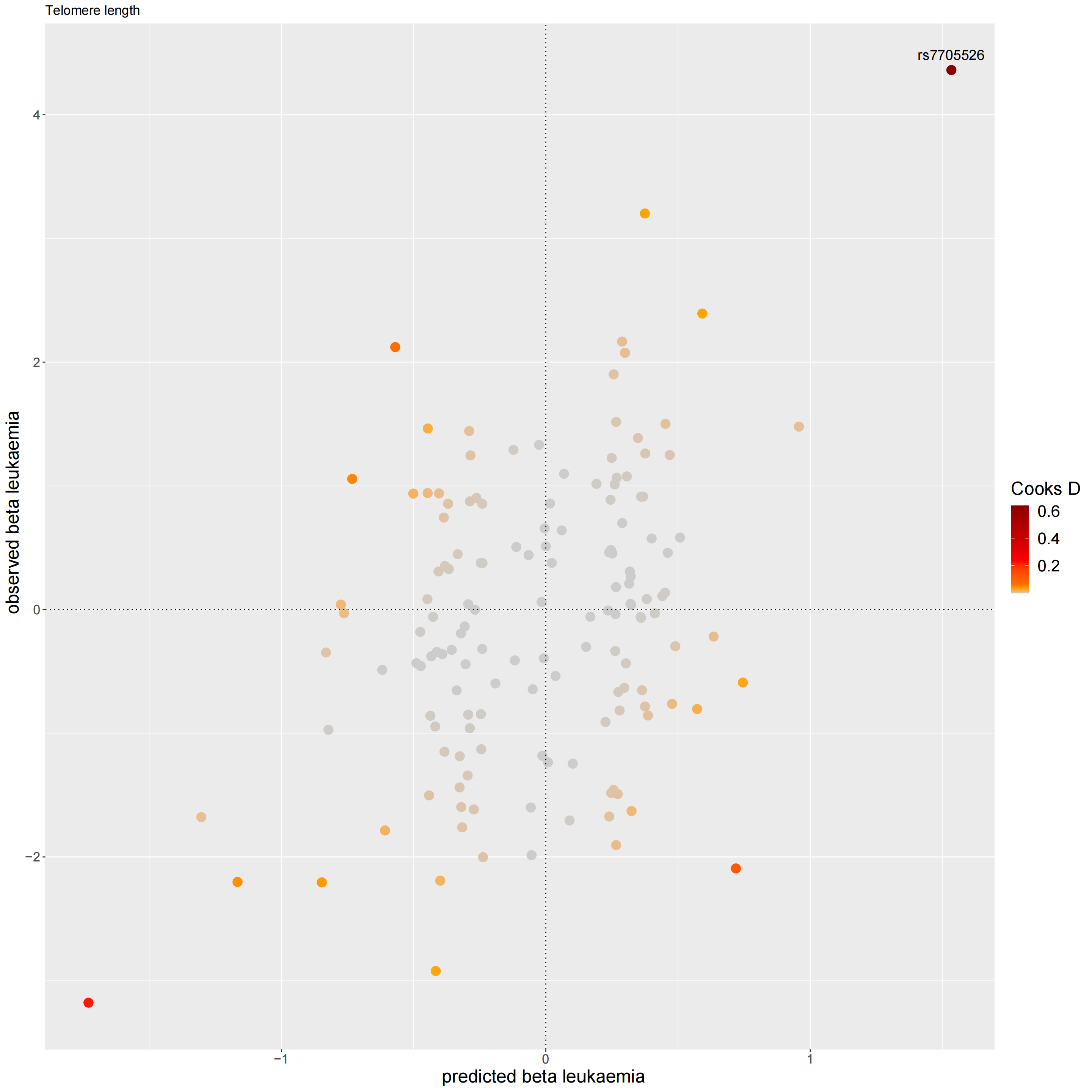


(b) the q-statistic for outliers.


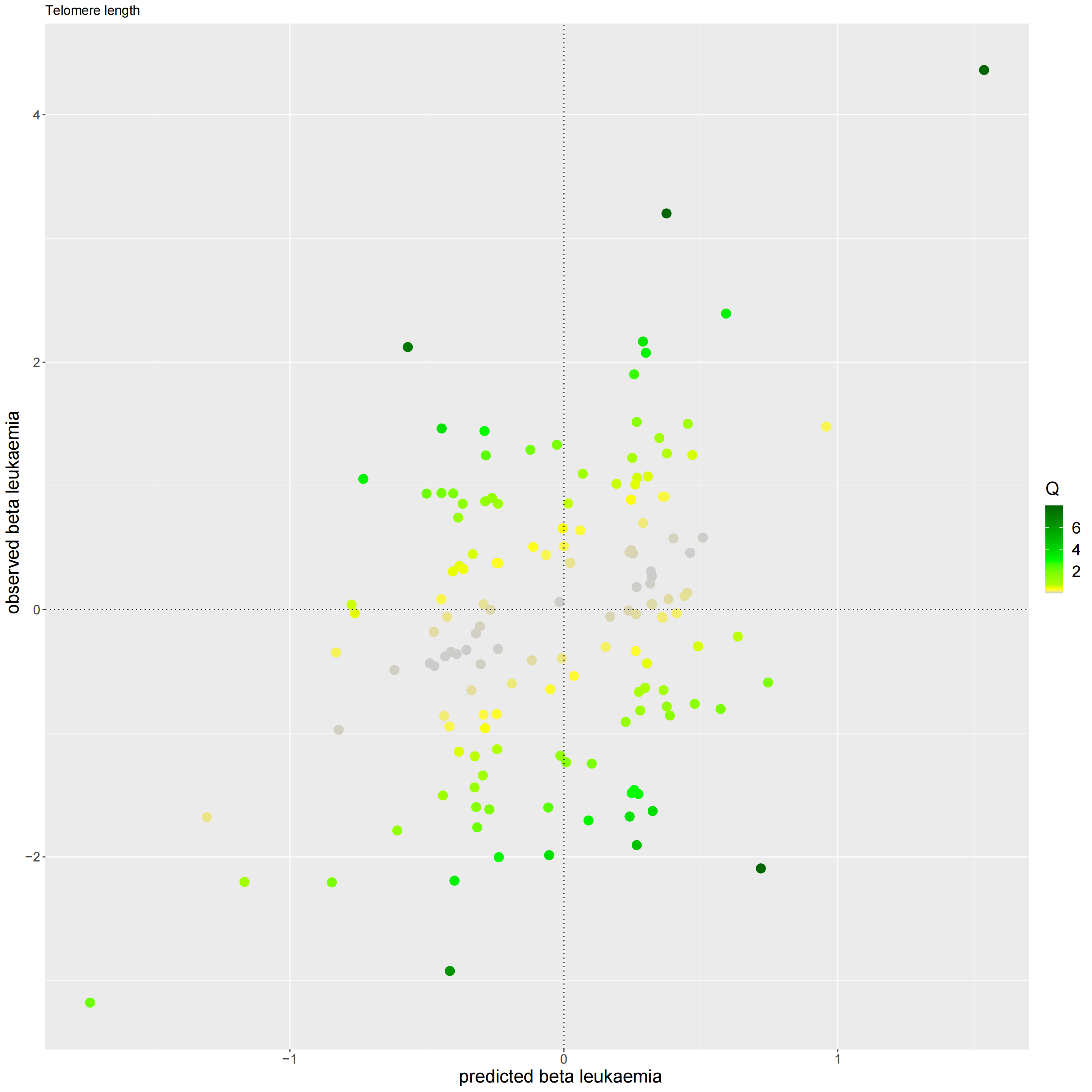


(2)

The predicted associations with acute lymphocytic leukaemia (discovery cohort) based on the model including telomere length (x-axis) are plotted against the observed associations with acute lymphocytic leukaemia (y-axis). These are the top models when keeping outliers and influential genetic variants in the analysis.

(a) Cook's distance for the influential points;





(b) the q-statistic for outliers.





(3)

The predicted associations with chronic lymphocytic leukaemia (discovery cohort) based on the model including telomere length (x-axis) are plotted against the observed associations with chronic lymphocytic leukaemia (y-axis). These are the top models when keeping outliers and influential genetic variants in the analysis.

(a) Cook's distance for the influential points;


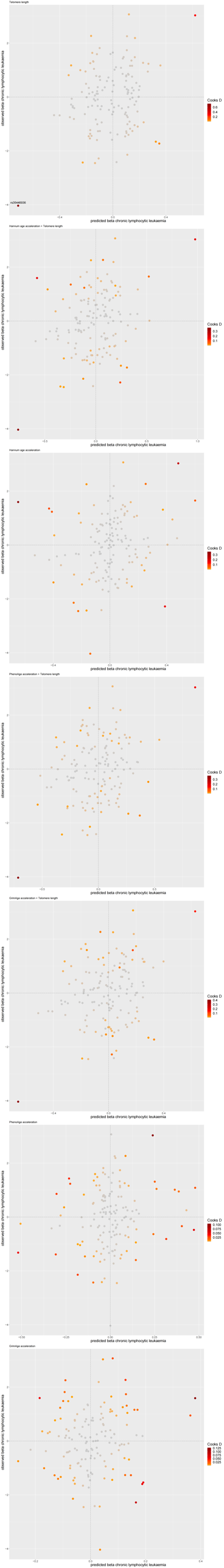


(b) the q-statistic for outliers.


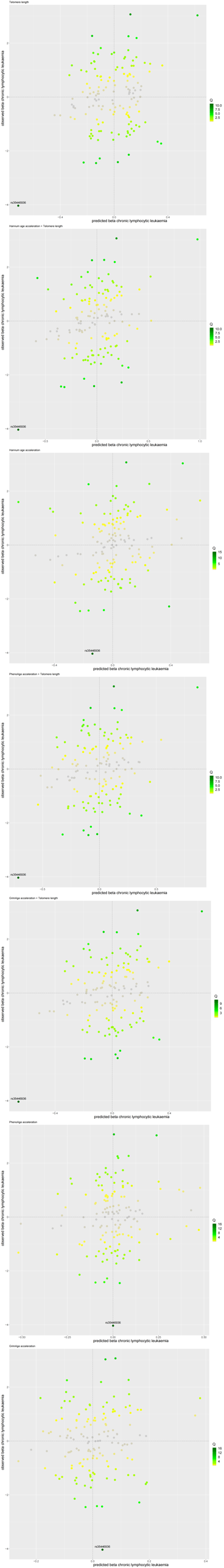


(4)

The predicted associations with chronic myeloid leukaemia (discovery cohort) based on the model including telomere length (x-axis) are plotted against the observed associations with chronic myeloid leukaemia (y-axis). These are the top models when keeping outliers and influential genetic variants in the analysis.

(a) Cook's distance for the influential points;


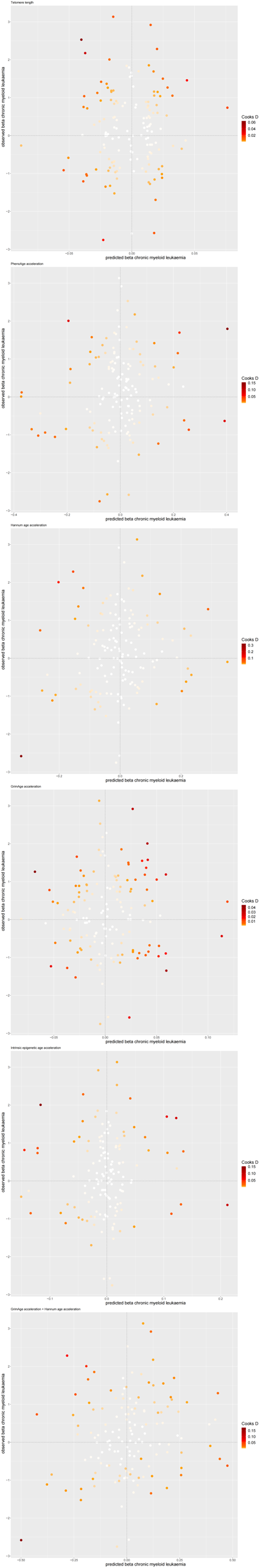


(b) the q-statistic for outliers.


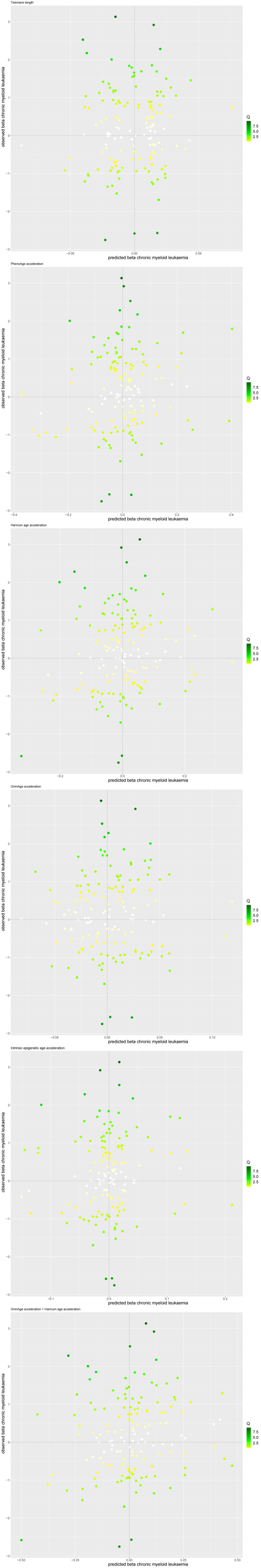


(5)

The predicted associations with essential thrombocythaemia (discovery cohort) based on the model including telomere length (x-axis) are plotted against the observed associations with essential thrombocythaemia (y-axis). These are the top models when keeping outliers and influential genetic variants in the analysis.

(a) Cook's distance for the influential points;


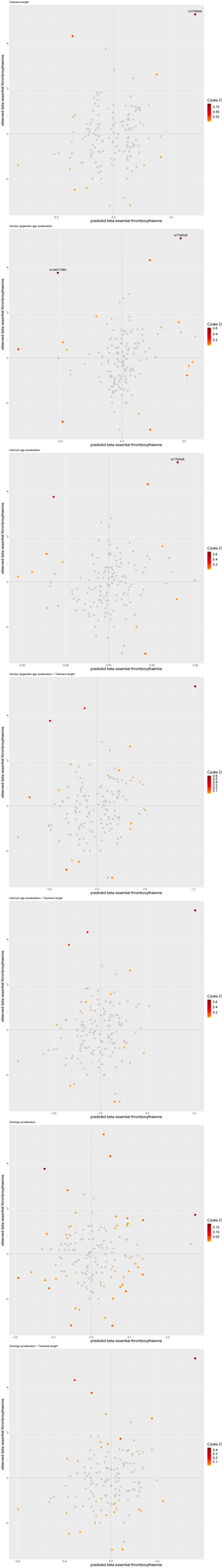


(b) the q-statistic for outliers.


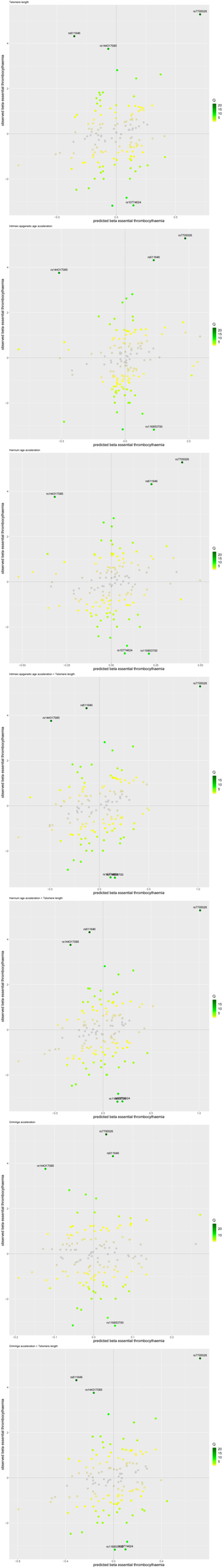


(6)

The predicted associations with hodgkin lymphoma (discovery cohort) based on the model including telomere length (x-axis) are plotted against the observed associations with hodgkin lymphoma (y-axis). These are the top models when keeping outliers and influential genetic variants in the analysis.

(a) Cook's distance for the influential points;





(b) the q-statistic for outliers.





(7)

The predicted associations with lymphoid leukaemia (discovery cohort) based on the model including telomere length (x-axis) are plotted against the observed associations with lymphoid leukaemia (y-axis). These are the top models when keeping outliers and influential genetic variants in the analysis.

(a) Cook's distance for the influential points;


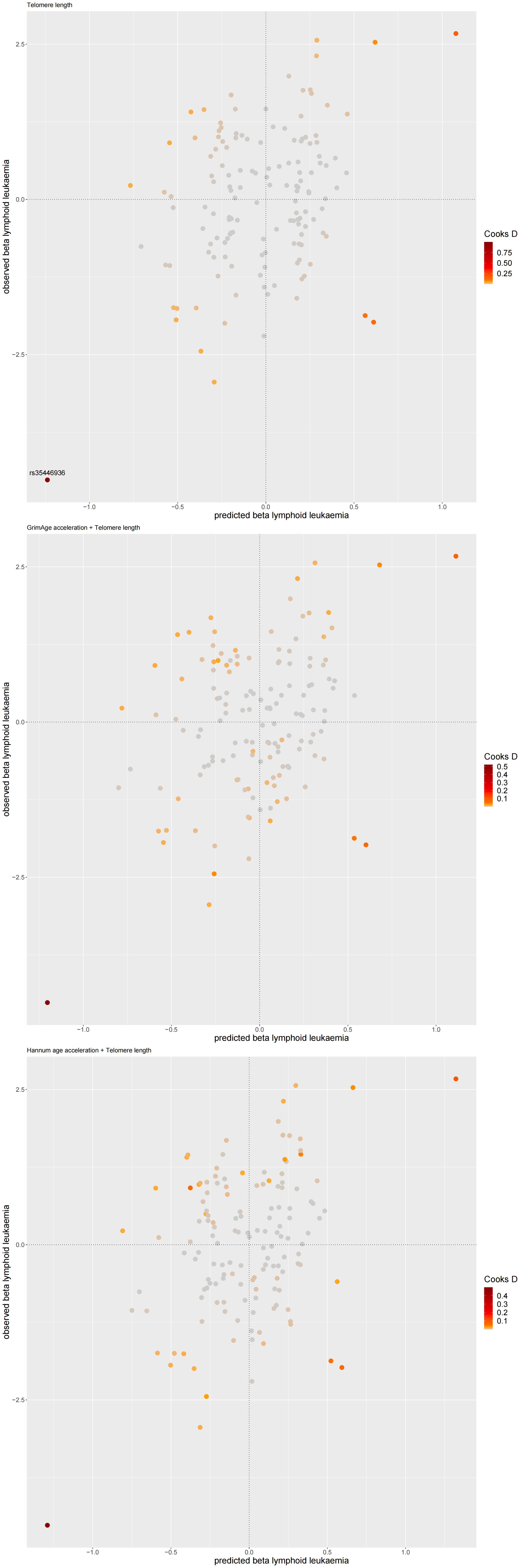


(b) the q-statistic for outliers.


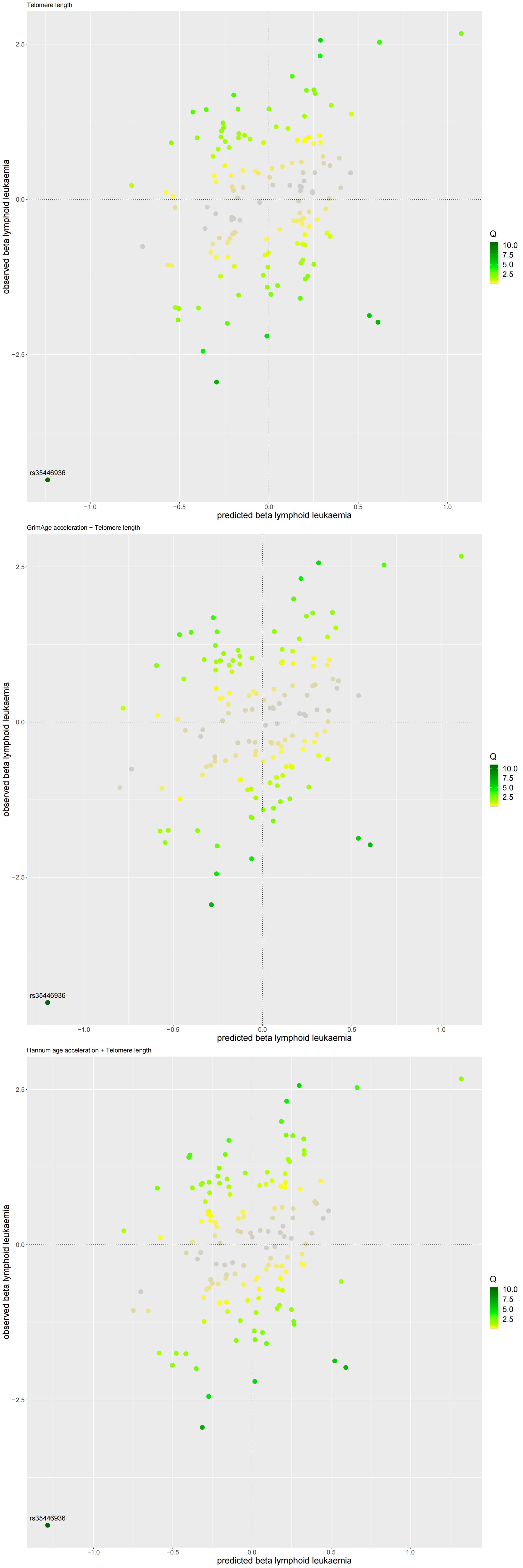


(8)

The predicted associations with lymphoid leukaemia (validation cohort) based on the model including telomere length (x-axis) are plotted against the observed associations with lymphoid leukaemia (y-axis). These are the top models when keeping outliers and influential genetic variants in the analysis.

(a) Cook's distance for the influential points;


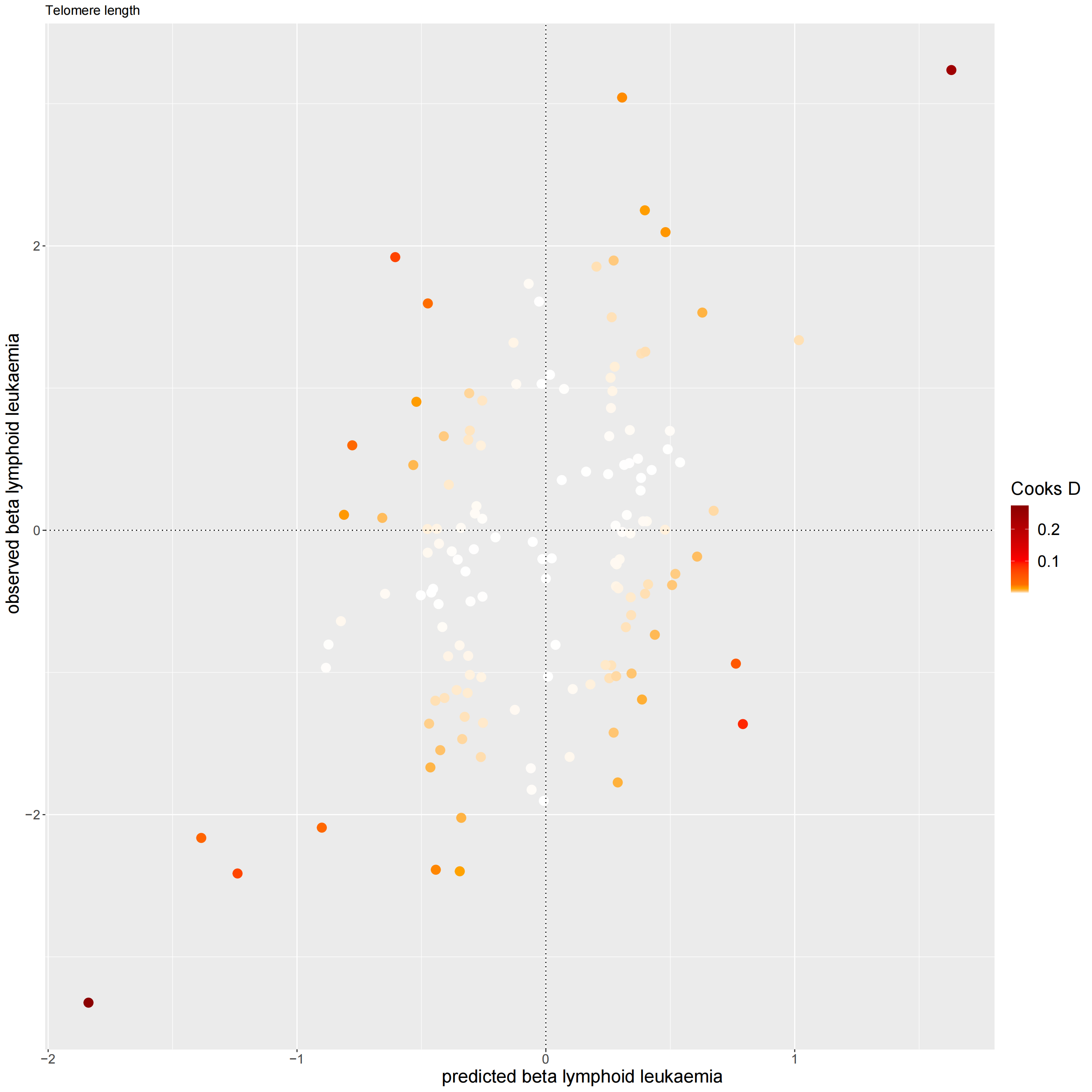


(b) the q-statistic for outliers.


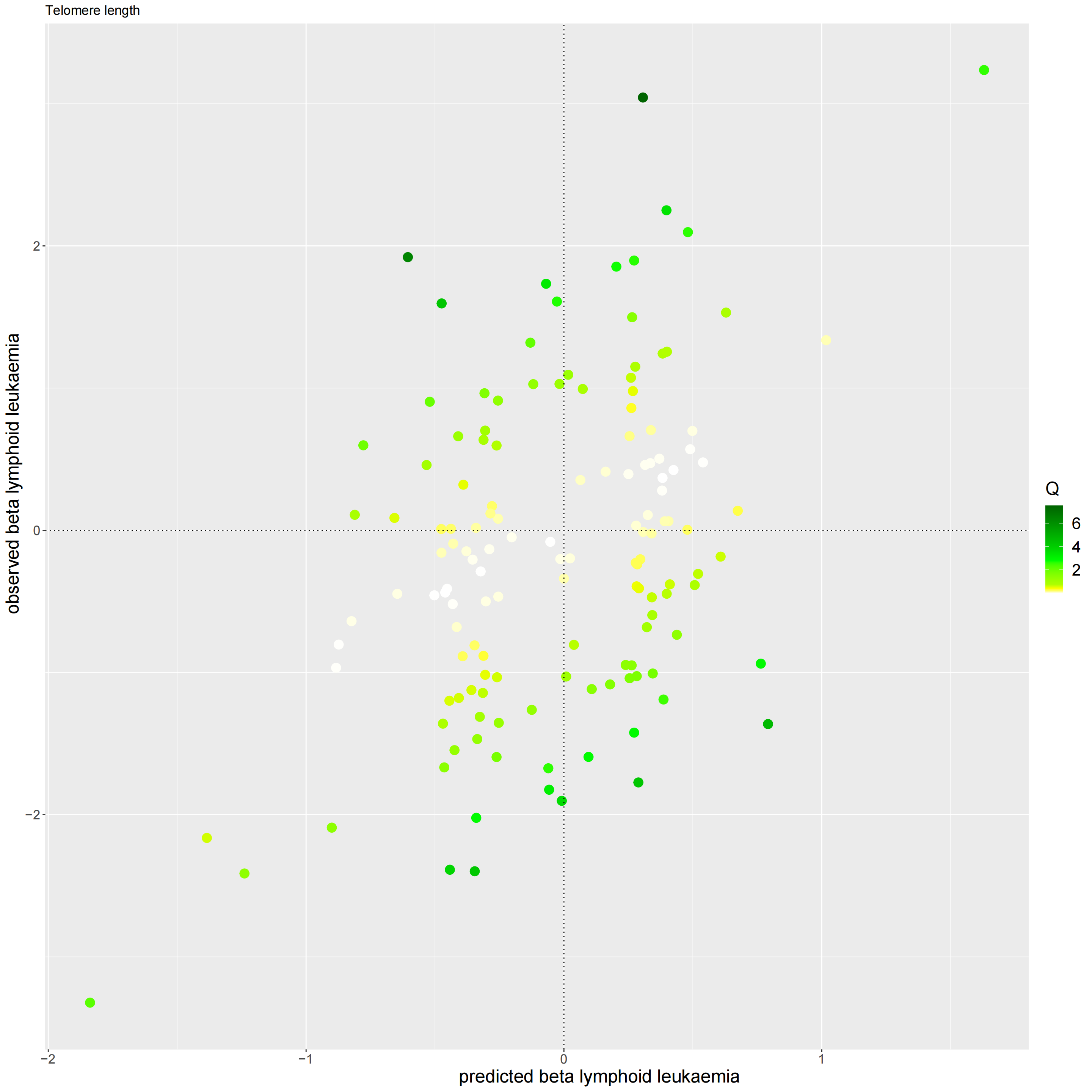


(9)

The predicted associations with lymphomas (validation cohort) based on the model including telomere length (x-axis) are plotted against the observed associations with lymphomas (y-axis). These are the top models when keeping outliers and influential genetic variants in the analysis.

(a) Cook's distance for the influential points;


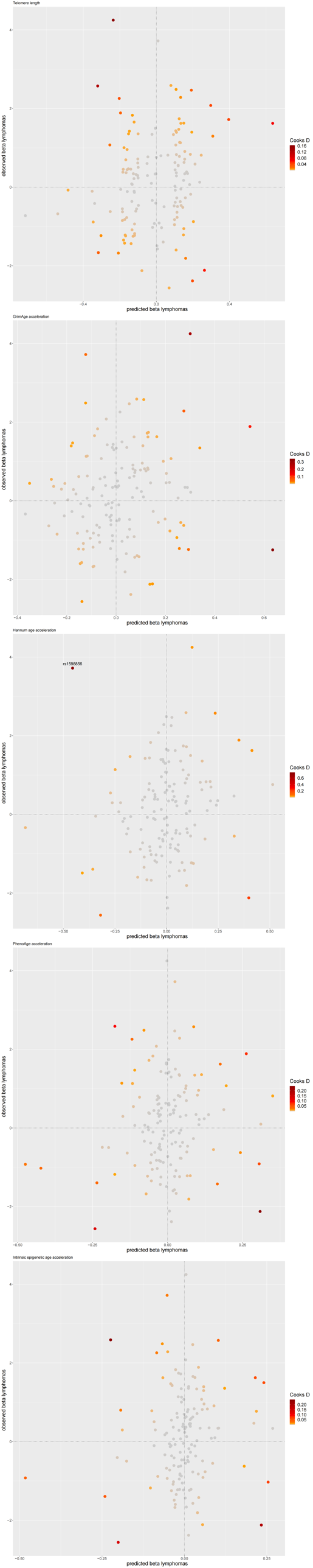


(b) the q-statistic for outliers.


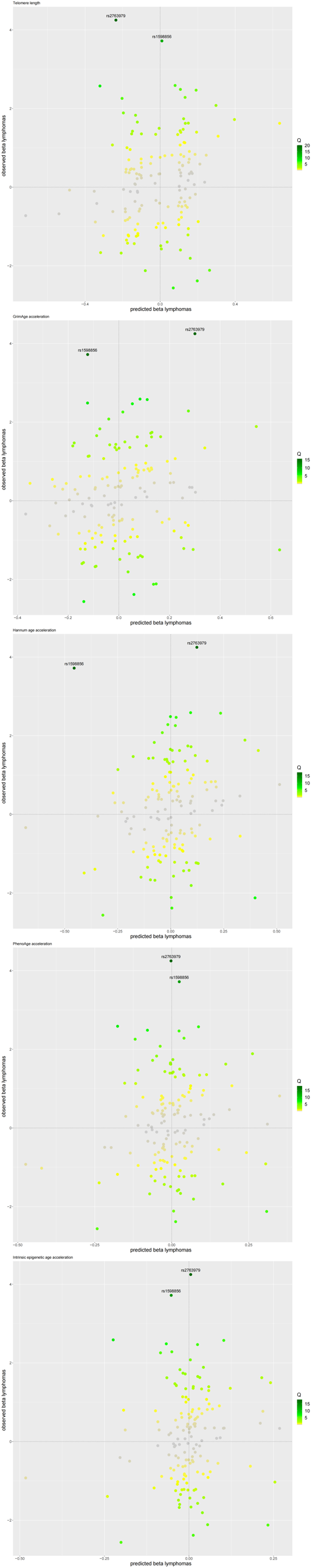


(10)

The predicted associations with malignant immunoproliferative diseases (discovery cohort) based on the model including telomere length (x-axis) are plotted against the observed associations with malignant immunoproliferative diseases (y-axis). These are the top models when keeping outliers and influential genetic variants in the analysis.

(a) Cook's distance for the influential points;


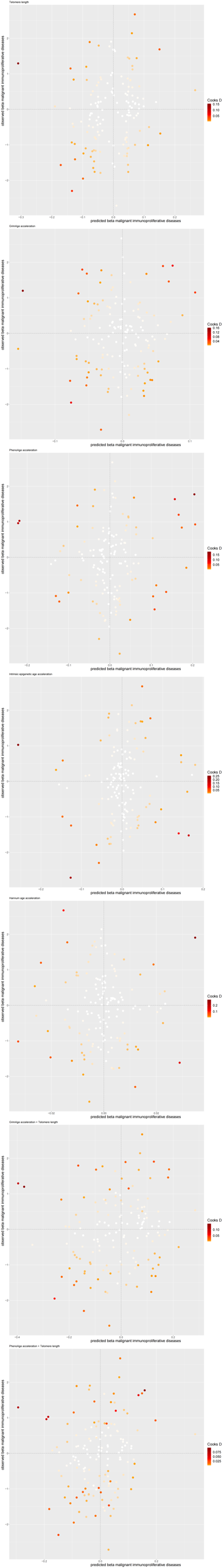


(b) the q-statistic for outliers.


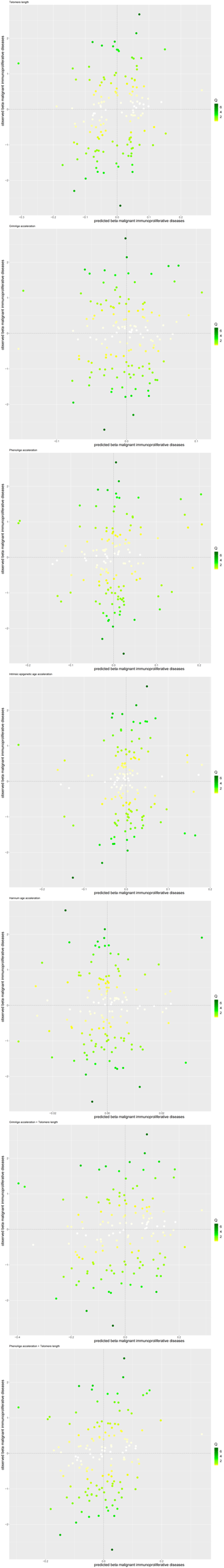


(11)

The predicted associations with multiple myeloma and malignant plasma cell neoplasms (discovery cohort) based on the model including telomere length (x-axis) are plotted against the observed associations with multiple myeloma and malignant plasma cell neoplasms (y-axis). These are the top models when keeping outliers and influential genetic variants in the analysis.

(a) Cook's distance for the influential points;


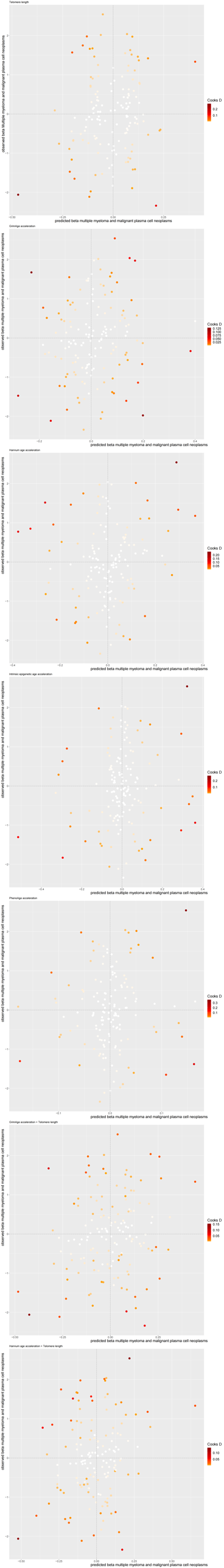


(b) the q-statistic for outliers.


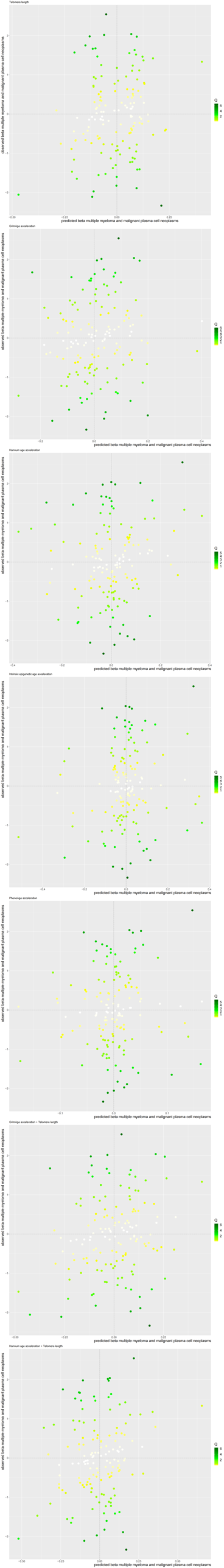


(12)

The predicted associations with multiple myeloma (validation cohort) based on the model including telomere length (x-axis) are plotted against the observed associations with multiple myeloma (y-axis). These are the top models when keeping outliers and influential genetic variants in the analysis.

(a) Cook's distance for the influential points;


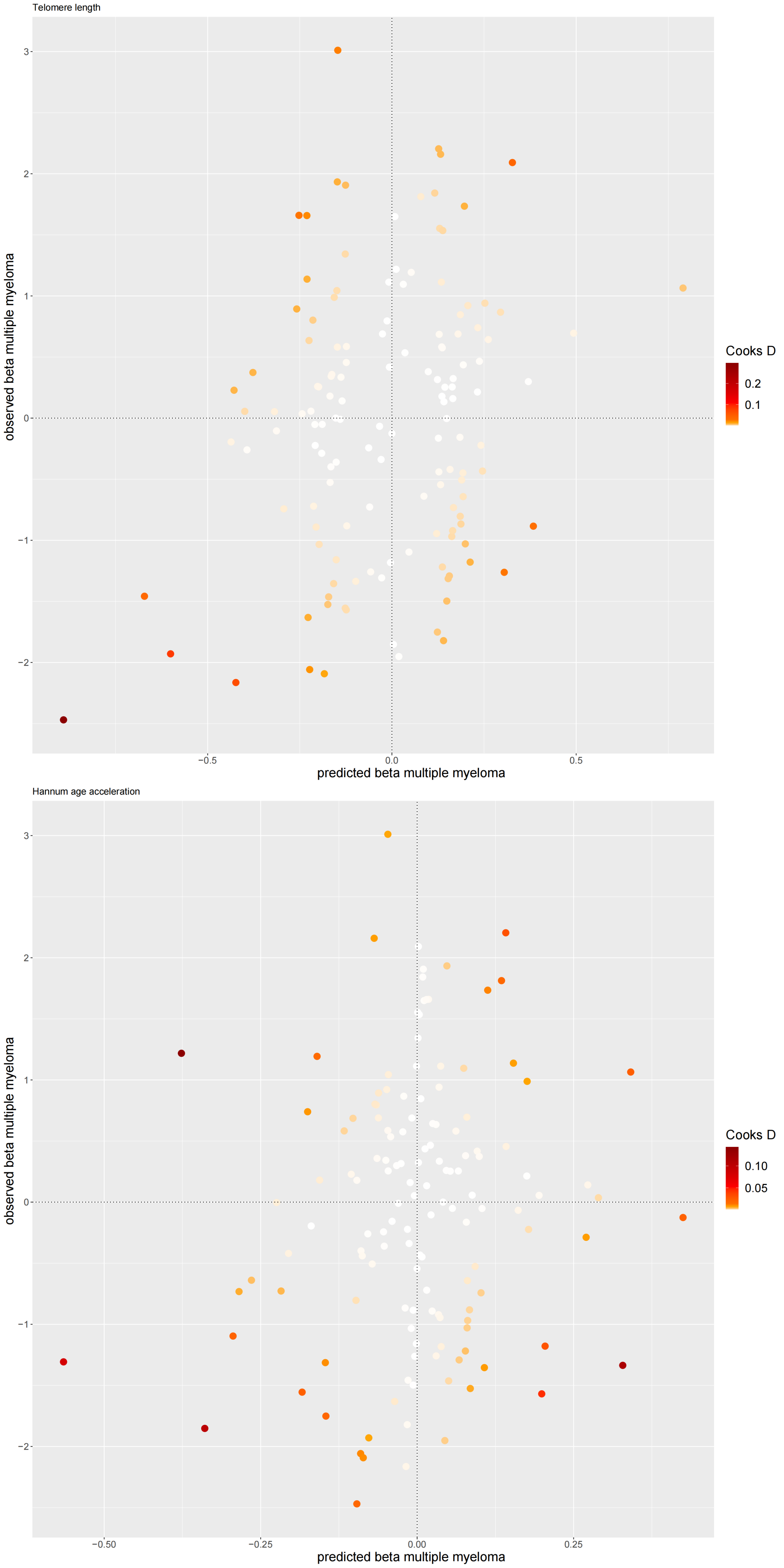


(b) the q-statistic for outliers.


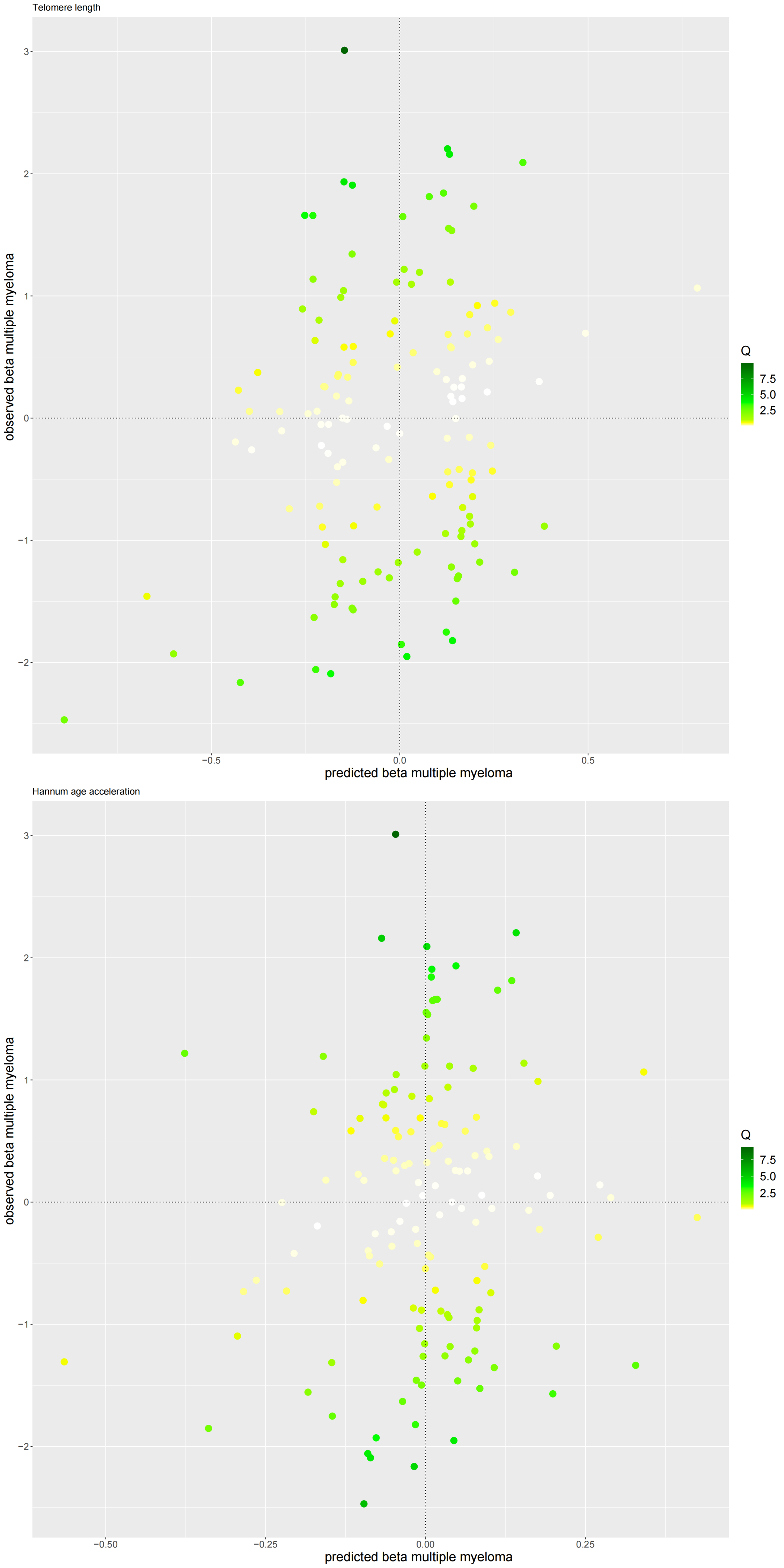


(13)

The predicted associations with myeloid leukaemia (discovery cohort) based on the model including telomere length (x-axis) are plotted against the observed associations with myeloid leukaemia (y-axis). These are the top models when keeping outliers and influential genetic variants in the analysis.

(a) Cook's distance for the influential points;


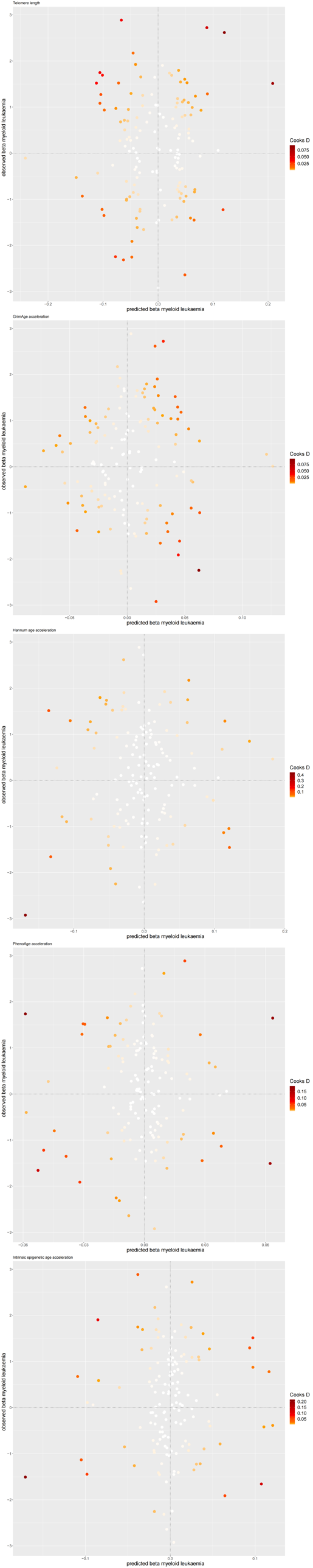


(b) the q-statistic for outliers.


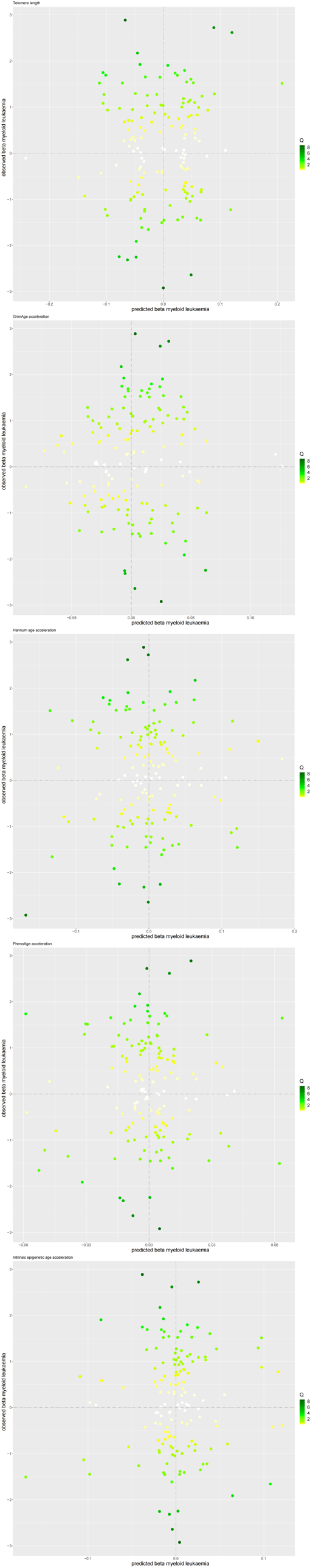


(14)

The predicted associations with myeloid leukaemia (validation cohort) based on the model including telomere length (x-axis) are plotted against the observed associations with myeloid leukaemia (y-axis). These are the top models when keeping outliers and influential genetic variants in the analysis.

(a) Cook's distance for the influential points;


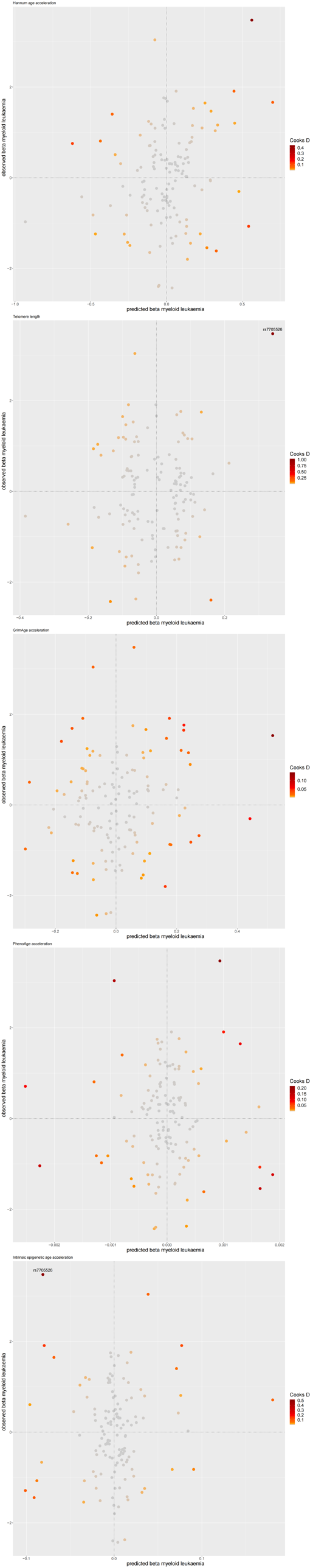


(b) the q-statistic for outliers.


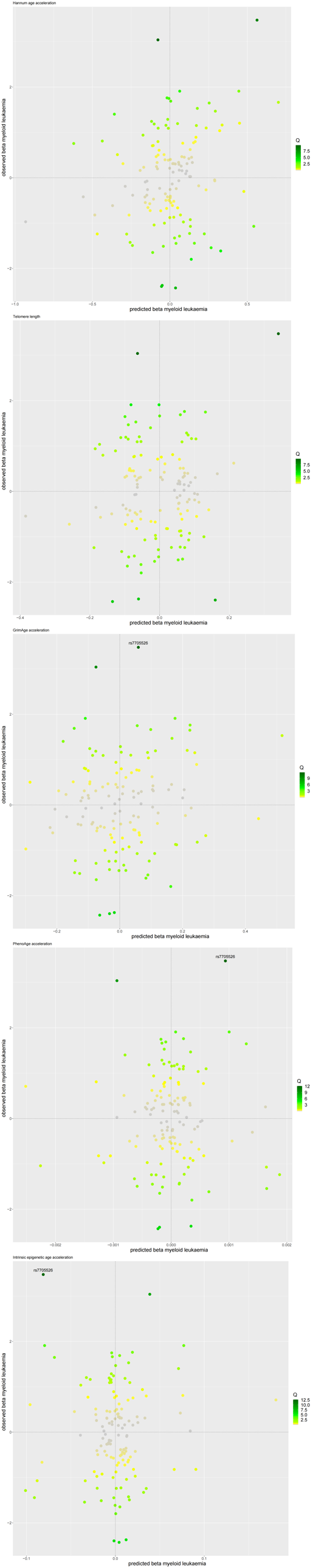


(15)

The predicted associations with non-follicular lymphoma (discovery cohort) based on the model including telomere length (x-axis) are plotted against the observed associations with non-follicular lymphoma (y-axis). These are the top models when keeping outliers and influential genetic variants in the analysis.

(a) Cook's distance for the influential points;


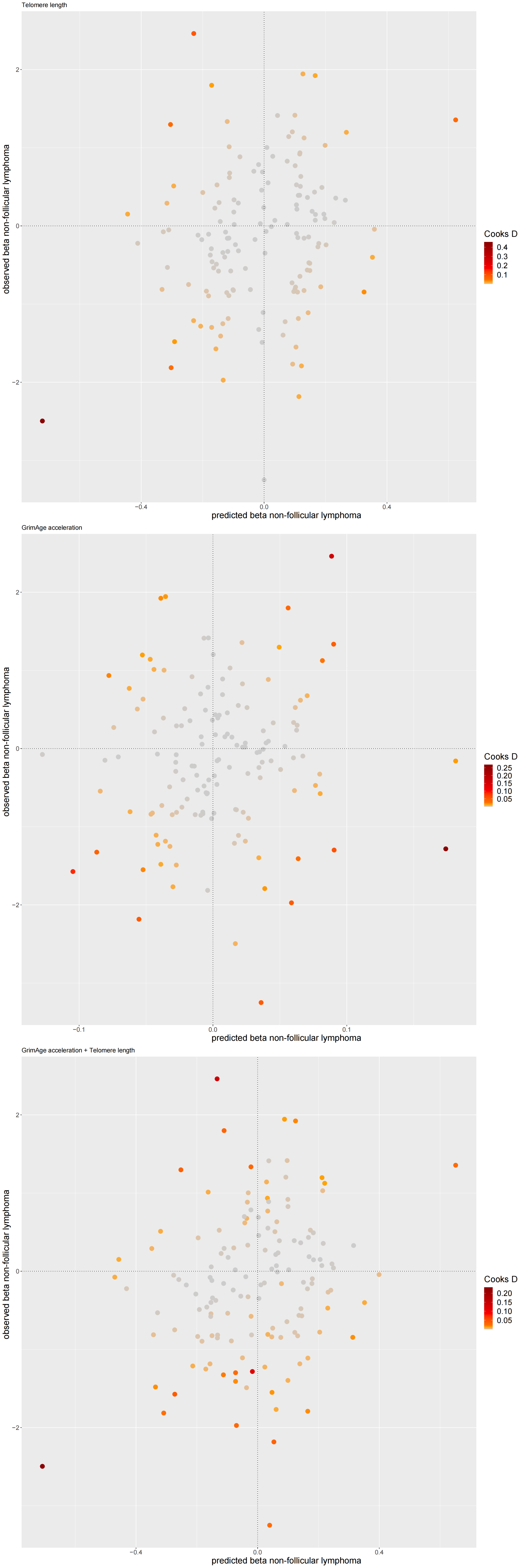


(b) the q-statistic for outliers.


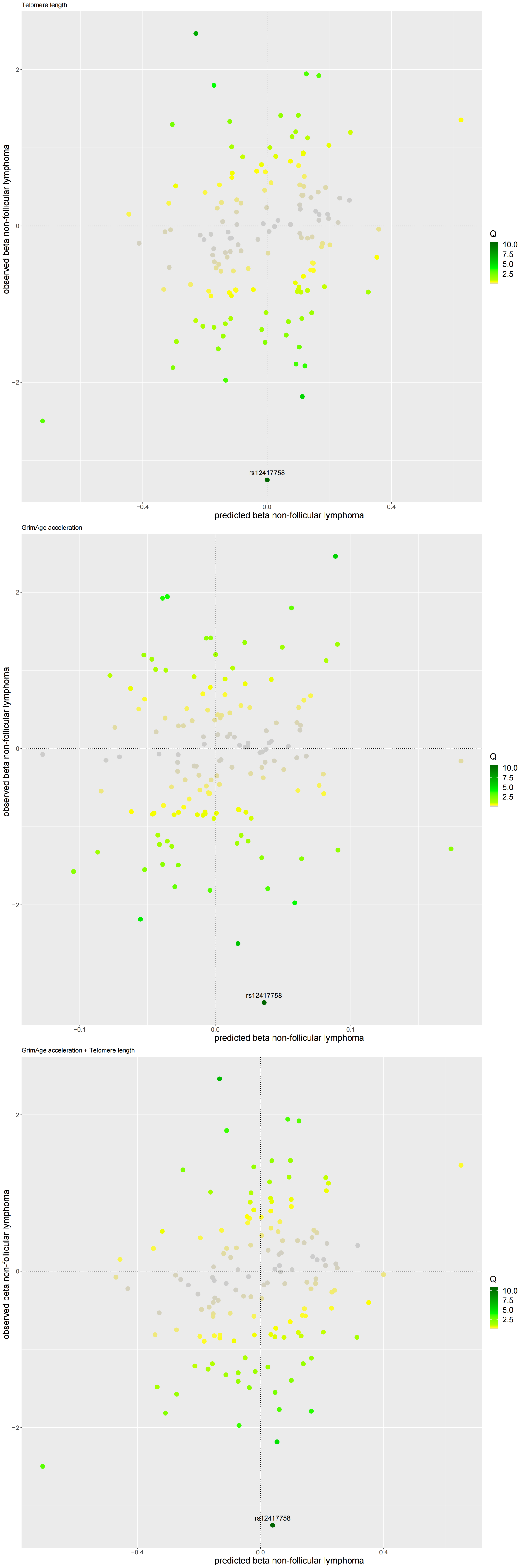


(16)

The predicted associations with non-Hodgkin lymphoma (discovery cohort) based on the model including telomere length (x-axis) are plotted against the observed associations with non-Hodgkin lymphoma (y-axis). These are the top models when keeping outliers and influential genetic variants in the analysis.

(a) Cook's distance for the influential points;


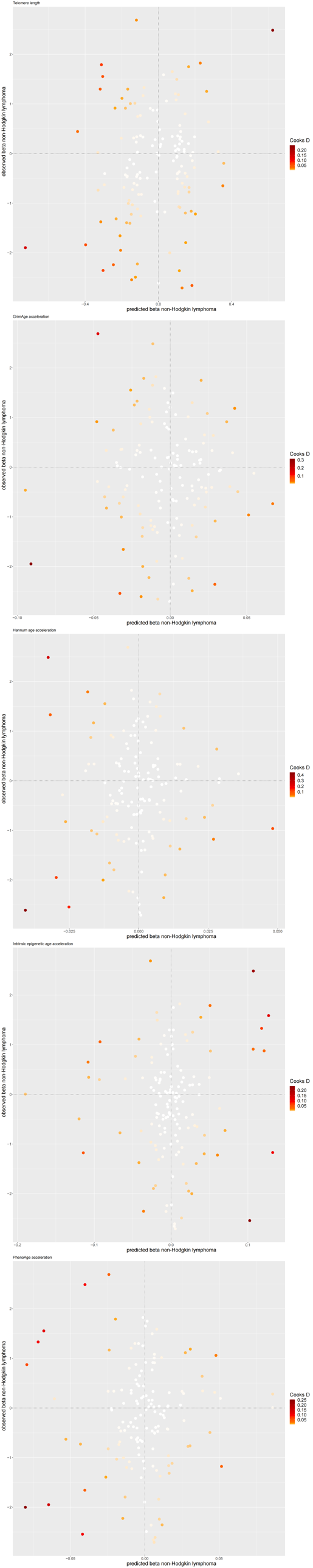


(b) the q-statistic for outliers.


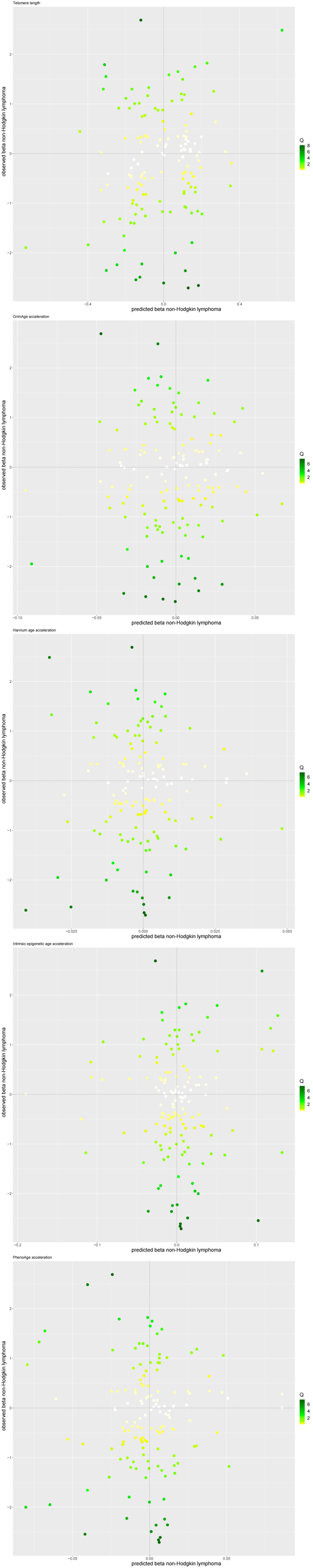


(17)

The predicted associations with other and unspecified iron deficiency (discovery cohort) based on the model including telomere length (x-axis) are plotted against the observed associations with other and unspecified iron deficiency (y-axis). These are the top models when keeping outliers and influential genetic variants in the analysis.

(a) Cook's distance for the influential points;


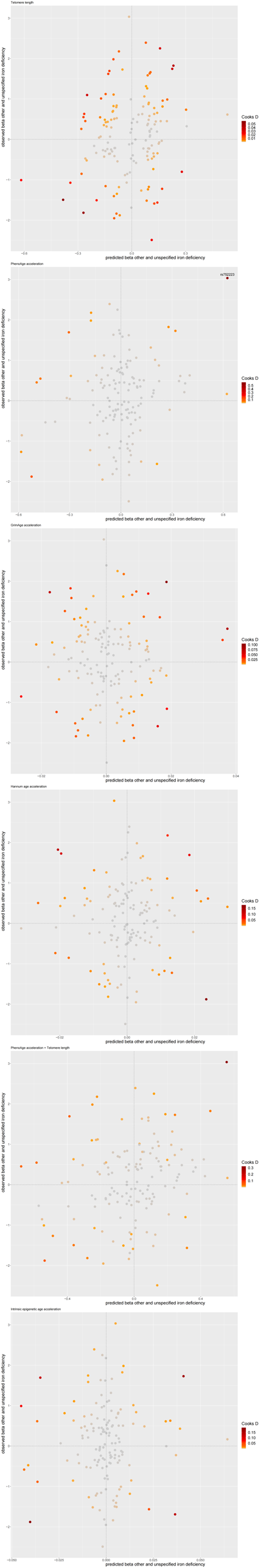


(b) the q-statistic for outliers.


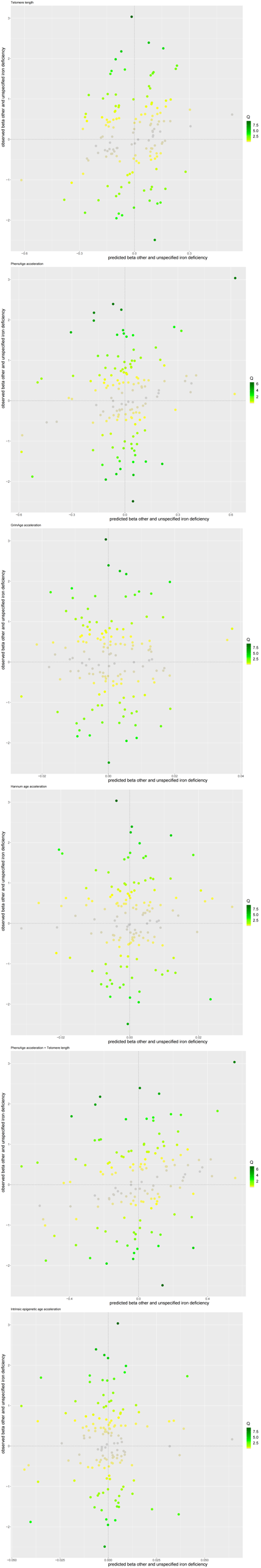


(18)

The predicted associations with other and unspecified coagulation defects (discovery cohort) based on the model including telomere length (x-axis) are plotted against the observed associations with other and unspecified coagulation defects (y-axis). These are the top models when keeping outliers and influential genetic variants in the analysis.

(a) Cook's distance for the influential points;


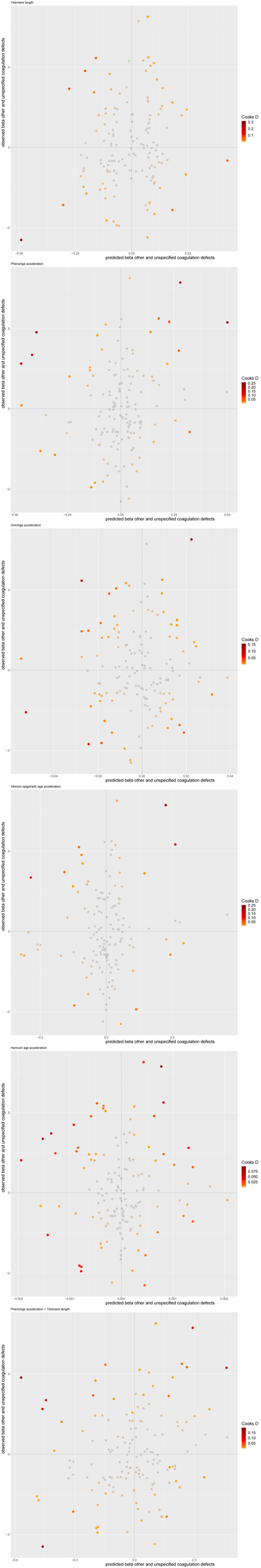


(b) the q-statistic for outliers.


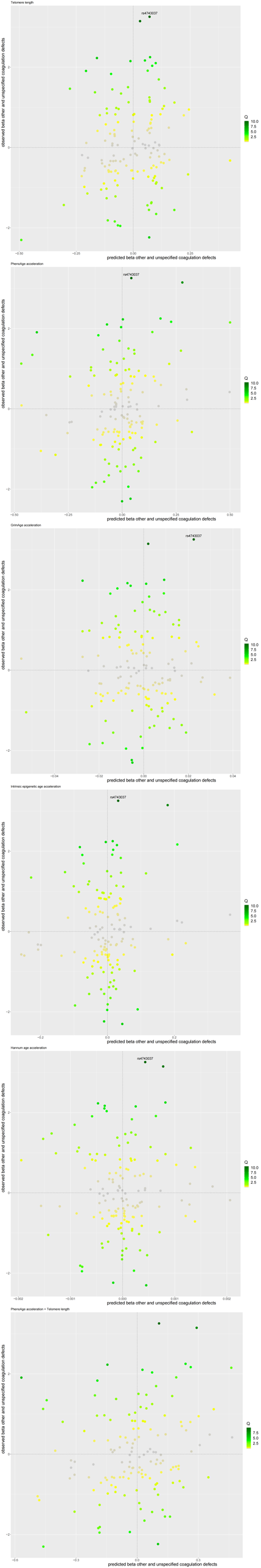


(19)

The predicted associations with other and unspecified types of non-Hodgkin lymphoma (discovery cohort) based on the model including telomere length (x-axis) are plotted against the observed associations with other and unspecified types of non-Hodgkin lymphoma (y-axis). These are the top models when keeping outliers and influential genetic variants in the analysis.

(a) Cook's distance for the influential points;


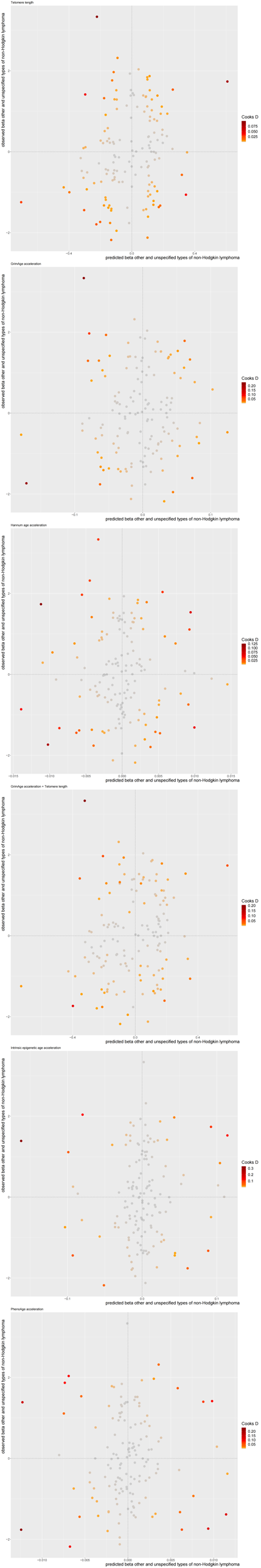


(b) the q-statistic for outliers.


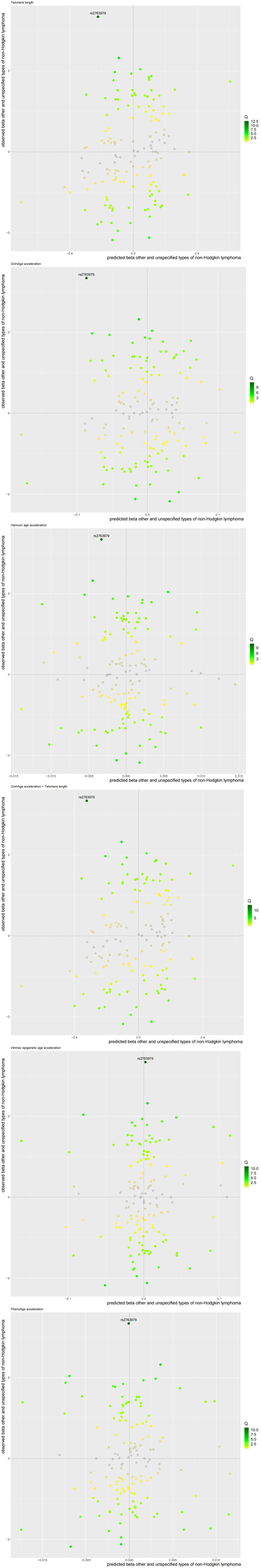


(20)

The predicted associations with other disorders of white blood cells (discovery cohort) based on the model including telomere length (x-axis) are plotted against the observed associations with other disorders of white blood cells (y-axis). These are the top models when keeping outliers and influential genetic variants in the analysis.

(a) Cook's distance for the influential points;


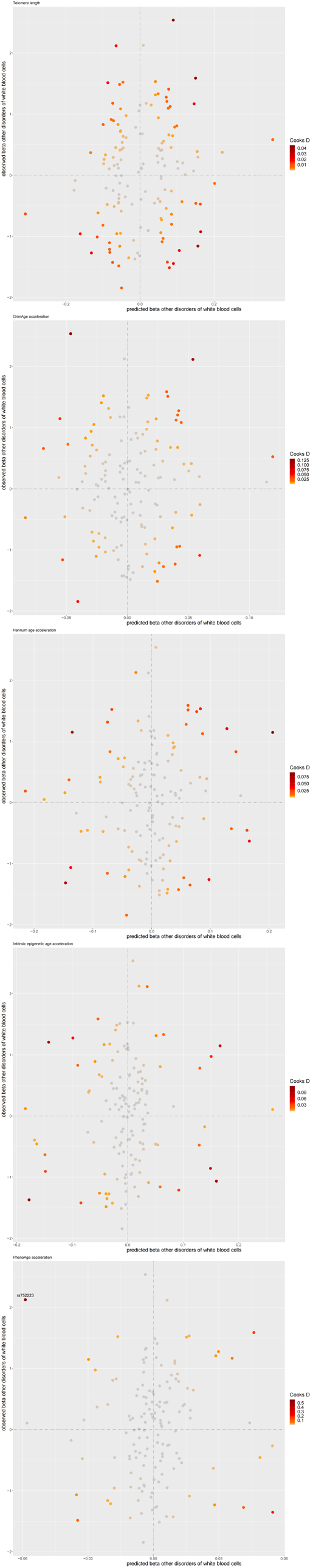


(b) the q-statistic for outliers.


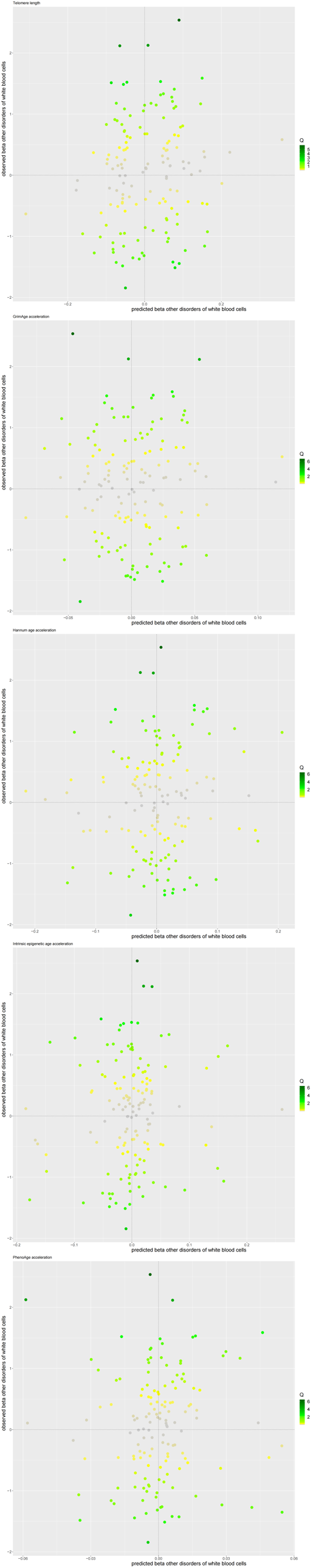

Supplement: Supplementary file 3 — Supplementary Figure 6 [file 41408_2024_1035_MOESM3_ESM.docx]
